# An Ontology for the Adoption of Medical Devices in Health Care Organizations: Design and Development Study

**DOI:** 10.2196/88366

**Published:** 2026-07-10

**Authors:** Sara Vannelli, Daniele Spoladore, Filippo Visintin

**Affiliations:** 1 Department of Industrial Engineering, University of Florence, Viale Morgagni 40-44, Firenze, Tuscany, 50134, Italy, 39 3669739204; 2 Institute of Intelligent Industrial Technologies and Systems for Advanced Manufacturing, National Research Council of Italy, Lecco, Lombardy, Italy

**Keywords:** ontology, decision support system, technology adoption, medical devices, health technology assessment, service innovation

## Abstract

**Background:**

The health care industry is witnessing a rapid proliferation of medical devices. Health care organizations need effective tools to identify devices that best align with their needs, and ensure seamless integration into clinical processes. Existing conceptualizations of expert knowledge remain fragmented, and no comprehensive decision support systems exist to assist stakeholders in evaluating and introducing new medical devices. Ontology-based approaches offer a promising avenue to formalize such complex, multidisciplinary knowledge.

**Objective:**

This study aims to develop and validate an ontology designed as the backbone of a decision support system to facilitate the informed adoption of medical devices in health care organizations.

**Methods:**

The ontology was developed using a 5-phase methodology: (1) Elicitation of knowledge through a systematic literature review, a review of existing conceptualizations, and expert interviews; (2) Conceptualization of a preliminary conceptual map; (3) Co-design of a refined map through focus groups with experts; (4) Development of the ontology using the Protégé ontology editor; and (5) Validation of the ontology through interviews with experts. Forty experts from 13 companies across 3 European countries participated, ensuring multidisciplinary coverage.

**Results:**

The resulting ontology provides a modular and comprehensive conceptualization of medical devices that balances granularity, conciseness, and practical relevance. It explicitly models key dimensions required for informed adoption decisions, including medical conditions addressed by the device, health services enabled, roles and activities of health care professionals, manufacturer-related information, medical device applications, and structured evidence derived from Health Technology Assessment reports. The ontology’s instantiability and practical applicability were validated by populating it with data from 4 Health Technology Assessment reports and by expert assessment, confirming its ability to address stakeholders’ core decision-making needs.

**Conclusions:**

This study presents a validated ontology to support the informed adoption of medical devices in health care organizations. It addresses a literature gap by providing a comprehensive, structured conceptualization of medical devices that meets stakeholders’ key information needs. By formalizing complex expert knowledge, the ontology lays a foundation for future research and the practical development of decision support systems that enable transparent, effective, and efficient medical device adoption.

## Introduction

Recent advancements in technologies such as the Internet of Things, artificial intelligence, and extended reality have greatly accelerated the development of medical devices (MDs) [1]. The Parliament of the European Union defines MD as “any instrument, apparatus, appliance, software, implant, reagent, material or other article intended by the manufacturer to be used, alone or in combination, for human beings for one or more [...] medical purposes” [2]. The MD sector has experienced substantial growth in recent years, reaching a market size of about US $184.61 billion in the United States [3] and about US $160 billion in the European Union [4].

Adopting MDs can potentially enhance the effectiveness and efficiency of health care processes [5,6]. However, the decision to integrate MDs into health care organizations is extremely challenging, requiring interdisciplinary knowledge and collaboration among numerous stakeholders, all within a context of rapidly expanding MD offerings and accelerating innovation [7,8]. In many European countries, the decision to adopt new MDs occurs at the facility level [9] and is typically initiated by senior clinicians [10]. Being at the forefront of patient care, clinicians provide valuable insights into the potential clinical benefits of adopting MDs [11]. Based on their recommendations, health care managers select MDs that align with the organization’s broader needs and strategic goals by evaluating not only clinical benefits but also regulatory requirements and financial and operational implications [12,13]. This phase is often supported by Health Technology Assessment (HTA) analysts, who create detailed reports (hereafter, HTA reports) to evaluate the diverse impacts of MDs on various stakeholders, including patients, health care providers, and the overall health care system. These evaluations typically adhere to standardized frameworks such as the EUnetHTA (European Network for Health Technology Assessment) Core Model 3.0 (hereafter, EUnetHTA) [14], which help determine the relevant impacts to include in the assessment. Heads of medical units and senior physicians play a critical role in this decision-making process due to their expertise in clinical pathways and their understanding of patients’ needs and requirements [10].

Integrating multiple information sources is of utmost importance in navigating such a challenging decision-making process. In this regard, accessing expert knowledge—that is, valuable knowledge from qualified individuals deriving from experience, technical practices, training, and research [15]—on MDs can play a central role in supporting adoption decisions. However, the knowledge that can be articulated, documented, and shared (ie, explicit knowledge [16]) and nonverbalized, intuitive, and unarticulated knowledge that resides in experts’ minds (ie, implicit knowledge) [16] on MDs is not easy to access and use. Explicit knowledge (eg, the one included in technical sheets, scientific articles, regulations and standards, existing HTA reports, and so on) is often context-specific and complex to access and interpret. Moreover, it may be unavailable for certain MDs. Implicit knowledge (eg, that of clinicians, managers, and HTA analysts) is difficult to make explicit due to the variety of contexts in which it is acquired.

Conceptualizations of expert knowledge on MDs, such as taxonomies or ontologies, have been proposed over time. However, these are incomplete, as they focus solely on specific aspects of the knowledge domain. Moreover, to the best of the authors’ knowledge, to date, there are no tools, such as knowledge-based Decision Support Systems (DSS), supporting health care service providers in the introduction of new MDs.

To fill this gap, this paper lays the foundation for developing a DSS to support MD adoption decisions in health care organizations by presenting a prototypical domain ontology [17] intended as the backbone for such a DSS. The targeted knowledge domain includes all MDs that have obtained the Conformité Européenne mark that fall under the aforementioned European Union definition.

## Methods

### Overview

**Figure 1.**
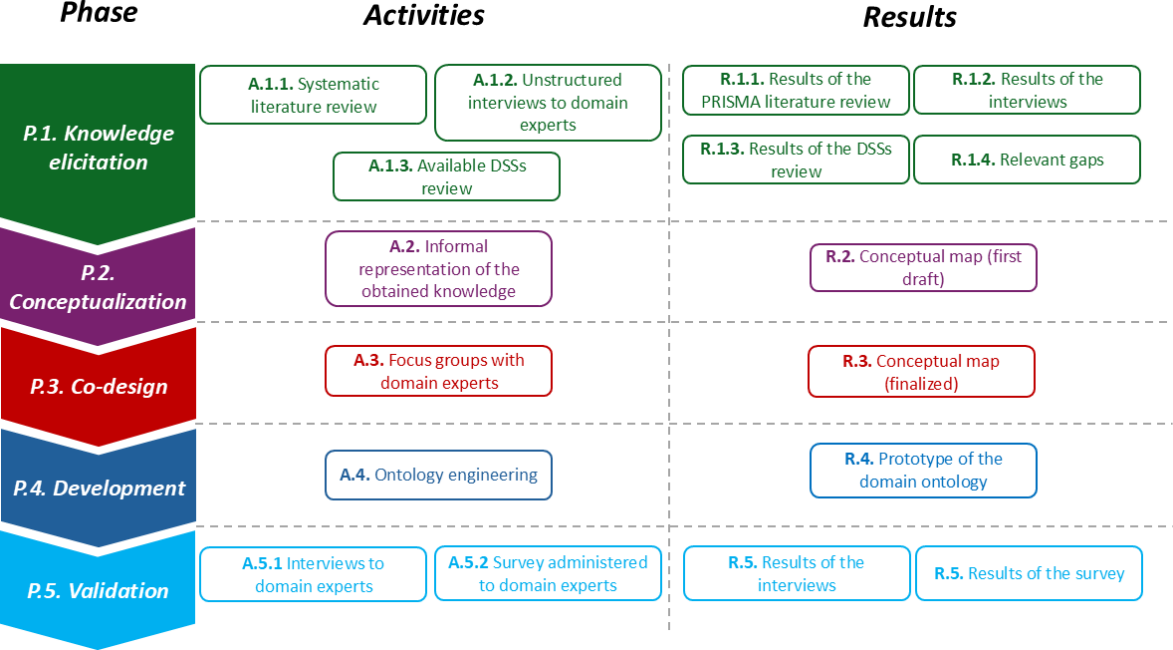
Custom 5-phase methodology. DSS: Decision Support Systems; PRISMA: Preferred Reporting Items for Systematic Reviews and Meta-Analyses.

The ontology was developed with the collaboration of 40 highly knowledgeable individuals from 13 companies located in 3 European countries, using a 5-phase methodology: (1) Elicitation of knowledge through systematic literature review and expert interviews (4 experts involved), (2) Conceptualization of a preliminary conceptual map, (3) Co-design of a refined map through 5 focus groups (23 experts involved), (4) Development of the ontology, and (5) Validation of the ontology (13 experts involved). Figure 1 shows the phases and their activities and outputs. The phases are described in the following paragraphs.

The ontology was developed by applying a knowledge-based approach since there is a lack of datasets about the knowledge domain. Indeed, no available dataset was retrieved, except for a few covering peculiar aspects of the US MD market, such as the one on “Manufacturer and User Facility Device Experience” [18]. These datasets do not cover the areas of interest in our research and do not address the European market. The fundamental knowledge-based ontology engineering activities [19] have been deeply rooted in a collaborative approach to foster the acquisition of relevant information and result in more reliable ontological models [20-22]. This adaptation was necessary due to the complexity and multidisciplinary nature of the knowledge to be conceptualized [23,24]. Specifically, in this study, the authors validated the effectiveness of using focus groups as a tool for knowledge acquisition and conceptualization phases in ontology engineering [25].

### Phase 1: Knowledge Elicitation

In the first phase*,* the knowledge relevant to the domain was elicited through a literature review and expert interviews, and the authors also analyzed any available DSS used by those responsible for deciding whether and how to introduce MDs into their organizations.

### PRISMA Literature Review

The literature review was guided by the PRISMA (Preferred Reporting Items for Systematic Reviews and Meta-Analyses) statement approach [26] and aimed at investigating the existing conceptualizations of MDs. In particular, the literature review aims to answer the following questions (Qs):

Q1. What types of conceptualizations are adopted?

Q2. What are the purposes of the conceptualizations?

Q3. What are the intended users of the conceptualizations?

Q4. What is the domain of knowledge conceptualized?

Q4a. What are the main conceptual items addressed?

Q4b. Which knowledge sources (eg, standards, regulations, ontologies, and so on) guide the conceptualizations?

Q5. Have the identified conceptualizations been adopted in any applications?

Q6. Which EUnetHTA assessment elements are covered by the conceptualizations?

Q6 referred to the EUnetHTA [27], as the European Union has recognized it as the official model for HTA reports. The EUnetHTA suggests a list of Assessment Elements (ie, factors, concerns, objectives, or preferences relevant to the decision problem) that should be considered when assessing MDs. Q6 was included to check whether existing conceptualizations make accessible the knowledge that stakeholders should look for when assessing MDs’ value.

We searched Clarivate ISI Web of Science, Scopus, PubMed, Cochrane Database, and ProQuest for English-language journal articles, reviews, book chapters, and conference proceedings published after 2001 in “Computer science,” “Engineering,” “Decision sciences,” “Medicine,” “Business, Management and Accounting,” and “Multidisciplinary” subject areas. The resulting query searched for (ontolog* OR taxonom* OR thesaur* OR conceptualization* OR “conceptual map” OR “conceptual model” OR “decision support system” OR “decision-support system” OR dss OR “knowledge-based system” OR “expert system”) AND (medical AND devic*) in the “Title,” “Keywords,” and “Abstract” fields. The search was conducted in April 2024 and led to the analysis of 20 papers. In April 2026, the search query was rerun to verify whether relevant papers had been published in the meantime. Three additional relevant papers were found [28-30]. These works were not available when the initial conceptual map was developed during Phase 2; however, for completeness, they are analyzed and discussed in the “Results” section together with the other 20 works retrieved in April 2024. It is necessary to note that, although pertinent to the topic, these additional contributions do not address the gaps identified in April 2024, thus maintaining the relevance of this study. Please see Checklist 1 for further details regarding the PRISMA protocol we followed in this study.

### Expert Interviews

Four participants (2 health care managers and 2 HTA analysts) from 2 leading hospitals in Italy were recruited for interviews using a purposive sampling strategy based on their expertise and direct involvement in MD adoption decisions. Additional details on the participants are reported in the “Results” section. Participants were identified through the authors’ professional networks.

Semistructured interviews were conducted, each lasting approximately 45 minutes. The interviews aimed at identifying the tools and sources experts usually use in MD’s adoption decisions and the problems they may face. Example prompts included (1) who is responsible for deciding which MDs to introduce, (2) which criteria are used when selecting and comparing MDs, (3) how the evaluation process is typically conducted, (4) what challenges are encountered in the selection and comparison of MDs, and (5) what type of information is required to support decision-makers and how a DSS could support this process.

Please see Checklist 2 for further details regarding the protocol for qualitative research we followed in this study.

### Phase 2: Conceptualization

The second phase aimed to draft the first version of a conceptual map [31], leveraging the knowledge obtained in Phase 1. The literature review and expert interviews were jointly used to identify relevant gaps that the ontology should address, to gain insights into how the decision-making problem is currently solved, and to identify and consolidate the main conceptual elements of the domain, including entities, attributes, and relationships relevant to MD adoption. The synthesis followed an iterative and incremental process. First, key concepts and relationships emerging from the literature were extracted and organized into preliminary conceptual elements. These were then refined and complemented using insights from expert interviews, which provided contextual validation and highlighted practical requirements and decision-making needs.

The resulting knowledge was formalized into a conceptual map, a structured glossary, and a set of competency questions—that is, natural language questions defining and constraining the scope of the knowledge represented in the ontology [32]. The conceptual map resulting from Phase 2 was implemented using Draw.io (Gaudenz Alder) and is presented in the “Results” section.

### Phase 3: Co-Design

The conceptual map resulting from the previous phase was adopted as the basis for the third phase, during which focus groups with domain experts were organized. Focus groups have proven effective in “co-designing” activities to elicit expert knowledge [33,34].

Domain experts were recruited through the authors’ professional networks using a purposive sampling strategy based on their roles and their direct involvement in MD adoption processes. Participation was voluntary, and experts were included to ensure a balanced representation of relevant stakeholders involved in the decision-making process. The experts who participated in the focus groups were health care managers, clinicians, HTA analysts, or MD manufacturers (ie, people involved in MD design, development, and sale). These latter were included because they have a comprehensive knowledge of the MDs and the sources (eg, databases) used for developing and selling MDs. Additional details on the participants are reported in the “Results” section.

Focus groups were conducted using a semistructured approach guided by the competency questions developed in Phase 2. During the sessions, participants were asked to comment on a populated version of the conceptual map based on a real MD and to identify missing elements or relationships. Each session lasted approximately 1 hour. Focus groups were conducted iteratively until no substantively new conceptual insights, missing conceptual elements, or relevant refinements to relationships emerged from focus group sessions.

All focus group sessions were recorded and transcribed verbatim. The transcripts were analyzed using thematic synthesis. Two authors independently coded the data, and discrepancies were resolved through discussion to reach consensus. Codes were iteratively grouped into higher-level themes, which were used to refine and extend the conceptual map, thereby improving the granularity (ie, the map allows the identification of the main relevant conceptual items and the relationships), instantiatability (ie, the map could be instantiated), and conciseness and completeness (ie, the map avoids any superfluous conceptual items and encompasses all essential ones) of the map [31,35]. The map resulting from this Phase is presented in the “Results” section.

### Phase 4: Development

The map resulting from the co-design phase was implemented into an ontology using the Protégé ontology editor [36]. Resource Description Framework [37] and Web Ontology Language [38] were adopted as ontological languages, with rules written in Semantic Web Rule Language [39]. The use of the Protégé ontology editor, together with W3C-endorsed languages for the semantic web, has become a common practice in biomedical and health care research, enabling semantic modeling, data integration, and knowledge-based decision support [40-42]. The prototypical ontology is described in the “Results” section and is available in Multimedia Appendix 1.

### Phase 5: Validation

The prototypical ontology developed in the previous phase was populated with data from 6 real MDs. Thirteen experts were then interviewed to assess whether the ontology met the information needs they typically have when comparing, evaluating, or introducing MDs. During the interviews, one of the authors presented examples, drawn from the 6 MDs used to populate the ontology, illustrating the knowledge represented in it. Interviewees could ask questions or comment throughout the interview, and afterward were invited to provide feedback and complete a questionnaire. Each interview lasted about 30 minutes. The questionnaire consisted of 13 Likert-scale items adapted from the subscales composing the Technology Acceptance Model 3 [43], aimed at assessing perceived usefulness, relevance, and intention to use the DSS that may be developed based on the ontology. The items were adapted to reflect the specific context of MD adoption and ontology-based decision support. In addition, 2 open-ended questions were included to capture perceived strengths and limitations of the ontology. Quantitative questionnaire responses were analyzed using descriptive statistics, while qualitative ones were analyzed through thematic coding to identify recurring strengths and limitations of the ontology. The evaluation metrics used to validate the ontology and the results of this phase are reported in the “Results” section.

### Ethical Considerations

The research was conducted in accordance with the Declaration of Helsinki and followed Good Clinical Practice guidelines. The study was performed within the Next Generation EU project ECS00000017 “Ecosistema dell’Innovazione” Tuscany Health Ecosystem (THE, PNRR, Spoke 9: Robotics and Automation for Health) and the project funded by NextGenerationEU-“Age-It-Aging well in an aging society” project (PE0000015), National Recovery and Resilience Plan (NRRP) -PE8-Mission4, C2, Intervention 1.3. This study did not involve clinical trials, the processing of sensitive patient data, medical record consultation, or sample archiving. According to national and regional regulations (Decreto Legislativo n. 211/2003, Legge Regione Toscana n.40/2005, Legge Regione Toscana n. 84/2015), ethics committee approval is not required for this type of research. Oral informed consent for meeting recording and the use of anonymized, aggregated data were obtained from all participants at the beginning of each session. All meetings were anonymized, and no identifying information was collected or stored. No identifiable features or personal data of participants are included in this publication. Data collection, processing, and management complied fully with national and European data protection regulations (EU Regulation Number 679/2016 and Decreto Legislativo Number 101/2018). Participants received no compensation or incentives for their participation.

## Results

### Overview

A collaborative approach was adopted to engineer the ontology [19]. Table 1 provides details about the experts supporting the development of the ontology, their role, and the Phase (1, 3, or 4) they were involved in. It is worth noticing that the experts involved in the ontology creation were not involved in the ontology validation to ensure the independence and objectivity of the evaluation process.

**Table 1.** Experts who participated in interviews (Phase 1), focus groups (Phase 3), or validation interviews (Phase 5).

Phase and number	ID	Expert group	Company	Country	KE^a^	FG^b^	VAL^c^
Phase 1: Knowledge elicitation							
1	KE1-Manager1	Manager	1	Italy	1		
2	KE1-Manager2	Manager	1	Italy	1		
3	KE2-HTA^d^	HTA analyst	1	Italy	2		
4	KE3- HTA	HTA analyst	2	Italy	3		
Phase 3: Co-design							
5	FG1-HTA	HTA analyst	3	Italy		1	
6	FG1-Manager	Manager	4	Italy		1	
7	FG1-Clinician1	Clinician	5	Italy		1	
8	FG1-Clinician2	Clinician	5	Italy		1	
9	FG1-Clinician3	Clinician	5	Italy		1	
10	FG1-Clinician4	Clinician	5	Italy		1	
11	FG1-Clinician5	Clinician	5	Italy		1	
12	FG2-HTA	HTA analyst	6	Italy		2	
13	FG2-Manufacturer1	MD manufacturer	7	Italy		2	
14	FG2-Manufacturer2	MD manufacturer	7	Italy		2	
15	FG3-Clinician1	Clinician	8	Italy		3	
16	FG3-Clinician2	Clinician	8	Italy		3	
17	FG3-HTA	HTA analyst	4	Italy		3	
18	FG4-Clinician1	Clinician	8	Italy		4	
19	FG4-Manager1	Manager	8	Italy		4	
20	FG4-Manager2	Manager	8	Italy		4	
21	FG4-Clinician2	Clinician	8	Italy		4	
22	FG4-Clinician3	Clinician	8	Italy		4	
23	FG4-Clinician4	Clinician	8	Italy		4	
24	FG5-Manufacturer1	MD manufacturer	7	Italy		5	
25	FG5-Clinician1	Clinician	8	Italy		5	
26	FG5-Manager1	Manager	9	Italy		5	
27	FG5- Manager2	Manager	9	Italy		5	
Phase 5: Validation							
28	VAL1-HTA	HTA analyst	10	Portugal			1
29	VAL2-Manager	Manager	11	Italy			2
30	VAL3-Manager	Manager	12	France			3
31	VAL4-Clinician	Clinician	8	Italy			4
32	VAL5-Manager	Manager	12	France			5
33	VAL6-Manager	Manager	5	Italy			6
34	VAL7-Manager	Manager	3	Italy			7
35	VAL8-HTA	HTA analyst	10	Portugal			8
36	VAL9-Manager	Manager	13	Italy			9
37	VAL10-HTA	HTA analyst	10	Portugal			10
38	VAL11-Manager	Manager	3	Italy			11
39	VAL11-Manager	Manager	3	Italy			11
40	VAL11-Manager	Manager	3	Italy			11
41	VAL12-Manager	Manager	6	Italy			12

a KE: knowledge elicitation interview in which the expert participated.

b FG: focus group in which the expert participated.

c VAL: validation interview in which the expert participated.

d HTA: Health Technology Assessment.

### Phase 1 Results: Knowledge Gained From Literature Review, Existing DSS Review, and Interviews

The knowledge elicitation phase includes 3 main activities: a systematic literature review, the analysis of existing DSSs on MDs, and expert interviews. This section presents the results of these activities, as well as the knowledge emerging from them.

### PRISMA Literature Review

**Figure 2.**
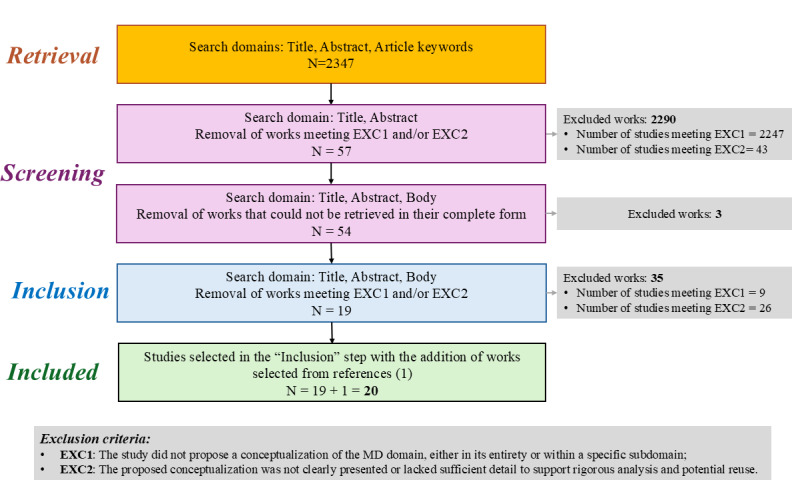
Phase 1: PRISMA (Preferred Reporting Items for Systematic Reviews and Meta-Analyses) flow followed in the literature review (search performed in April 2024). MD: medical device.

Figure 2 shows the PRISMA flow that the authors followed in the systematic literature review. Excluding duplicate works retrieved from more than one source, 2347 articles were retrieved from databases. The remaining works were scanned in their “Title” and “Abstract” fields to ensure consistency with the review aims; 57 works were retained. Works that could not be retrieved in their complete form were removed. Then, in the “Inclusion” phase, 2 authors read the screened works individually, and disagreements were resolved by a third author. Studies were excluded from the sample if they met one or more of the following exclusion criteria:

- EXC1: The study did not propose a conceptualization (eg, ontology, taxonomy, or conceptual model) of the MD domain, either in its entirety or within a specific subdomain (eg, portable MDs).
- EXC2: The proposed conceptualization was not clearly presented or lacked sufficient detail to support rigorous analysis and potential reuse.

The total number of scientific works selected in this way is 19.

Finally, in accordance with all researchers, one article from Dogmus et al [44] was included by looking at the articles referenced in the included papers. In the end, 20 papers were eligible.

The literature review results are presented in tabular form in Tables S1-S3 in Multimedia Appendix 2 and are briefly commented on in the text. Taxonomies and ontologies are the most frequently used types of conceptualizations (Q1), and only one study presents a conceptual map. Tables S1 and S2 provide an analysis of the literature concerning questions Q2, Q3, Q4, Q4a, Q4b, and Q5: Table S1 focuses on taxonomies, while Table S2 examines ontologies and conceptual maps. The reason behind this split relies on the fact that taxonomies and ontologies (and conceptual maps) are characterized by different granularities [31]. Therefore, they can be analyzed using different conceptual items: first-order categories for taxonomies are investigated in Table S1, while first-order classes with properties are investigated for ontologies and conceptual maps in Table S2. Each row of Tables S1 and S2 represents a study, and each column corresponds to one of the questions the literature review aims to answer (refer to the “Methods” section).

Looking at Tables S1 and S2, it can be noticed that conceptualizations in literature are characterized by different purposes (Q2) based on their intended users (Q3). They can support MD manufacturers in designing better MDs, clinicians in identifying MDs better fitting their needs, health care managers in improving the services they are responsible for, policymakers and HTA analysts in performing HTA assessments of different MDs, and patients and caregivers in choosing the best MD. Surprisingly, the intended user is sometimes not mentioned in the conceptualizations reviewed. The domain addressed (Q4) typically consists of a few types of MDs sharing some key commonalities (eg, wearable or portable devices). Only 4 conceptualizations consider the MDs’ domain as a whole. The conceptual items (Q4a) considered by the included works are various. Several properties are typically used to characterize the MD, such as the product code, lot number, nomenclature codes, manufacturer name, and MDs’ hardware and software components. These conceptual items are sometimes identified by resorting to existing public sources (Q4b; eg, European Medical Device Nomenclature [EMDN]). Other conceptual items include the resources needed to use an MD in a service, the potential users of the MD (mainly expressed in terms of targeted medical conditions, user impairments, or activity limitations), the stakeholders (other than the end user) who an MD could potentially impact, the health service within which the MD can be used, the location of use, the roles and tasks of those operating the MD, and the expected outcomes associated with the MD’s use. Finally, it has been proposed to provide links to scientific publications to help stakeholders access expert knowledge on MDs. Only 4 studies describe an application (Q5) leveraging the conceptualization presented. Half explicitly reference the conceptualizations’ role as a backbone for an application layer, and the other half only partially hypothesize or describe the application. The limited applicability of the works is also witnessed by the fact that, in only one case, there is a second work [44] that improves the application proposed by another [45].

Table S3 provides the information needed to answer Q6. The EUnetHTA outlines a comprehensive list of Assessment Elements, each designated by an alphanumeric code, representing a specific combination of Domain (ie, a broad category that groups assessment elements), Topic (subjects that characterize assessment elements), and Issue (ie, questions that need to be answered to evaluate the MD with regard to the assessment element). The EUnetHTA categorizes assessment elements into core (ie, the ones that should be included in all evaluations) and noncore ones and identifies 4 models (respectively for medical and surgical interventions, diagnostic technologies, screening technologies, and pharmaceuticals) that could differ in terms of core assessment elements. For each assessment element (corresponding to different rows in Table S3), the table provides its domain, topic, ID, issue, and a list of studies presenting conceptualization that have addressed this assessment element (even if they do not explicitly mention the EUnetHTA). Most of the assessment elements in the EUnetHTA (85%) have not been addressed in the reviewed conceptualizations. The selected conceptualizations mainly focus on identifying MD alternatives (B0001, 9 papers), describing the health conditions and populations (A0001, 8 papers), and describing the consequences and burden of the diseases targeted by the MD targets (A0009, 4 papers).

### Existing DSS

Our review highlighted that, although numerous DSS exist to assist various types of clinical decision-making [46,47], none have been specifically developed to support the evaluation of MDs and their introduction into health care organizations.

Currently, such evaluations can rely on a few databases, such as the European Database on Medical Devices (EUDAMED) [48], the “Public Access to Device Registration” (a database managed by the Medicines and Healthcare products Regulatory Agency on the MDs registered in the United Kingdom) [49], and the Global Unique Device Identification Database (a database managed by the FDA on MDs marketed in the United States) [50].

### Main Results of the Interviews

Interviews highlighted the need for a DSS to support MD introduction decisions and identified key stakeholder challenges. Interviews underscored that accessing expert knowledge could be essential to inform stakeholders in solving the decision problem concerning all the MDs for which proper HTA evaluations are not performed. Indeed, interviewees confirmed that, since HTA evaluations are time-consuming, they are typically reserved for MDs with significant expected clinical and economic impacts (ie, fewer than 5% of total purchase requests), while most MDs undergo less rigorous evaluations based on accessible available knowledge.

One of the main concerns raised by interviewees was the inadequacy of available knowledge-based apps and databases for solving the decision problem. For instance, interviewee INT3-HTA noted that EUDAMED provided only basic information and often contained incomplete records, limiting its usefulness. In addition, interviewee KE1-Manager1 highlighted the lack of detailed information on available databases on the organizational impact of MDs, including redistribution of staff tasks and modifications in health care service delivery processes. Moreover, interviewee KE1-Manager1 emphasized the need for a DSS that can assist them in comparing MDs that address the same medical condition and enable the provision of the same health service: “while clinicians often have specific MDs in mind when submitting purchase requests, for me it is still of paramount importance to evaluate alternative MDs.”

Furthermore, the interviews highlighted the relevance of the EUnetHTA while also emphasizing the challenges associated with using existing HTA reports developed under this framework. Indeed, in all the health care organizations where interviewees work, the EUnetHTA serves as a reference for conducting HTA assessments. However, as noted by interviewee KE2-HTA, accessing and using existing reports can be time-consuming and difficult, since they are produced by various national and international agencies, each following different reporting procedures.

Finally, it is worth noticing that none of the interviewees mentioned databases other than EUDAMED.

### Relevant Gaps

Neither the studies available in the literature nor the existing DSSs and databases adequately address the informational needs of stakeholders involved in the evaluation and selection of MDs.

Only a limited number of conceptualizations are available in the literature, and, to the best of the authors’ knowledge, only the EUDAMED database [48] considers the MD domain as a whole. Moreover, several conceptual elements that experts identified as essential to support decision-making during the interviews are either missing or only partially addressed. For example, the identification of medical conditions in which an MD can be used represents the starting point of most decision-making problems, as highlighted by both the literature [10] and the experts interviewed. Yet, to the best of the authors’ knowledge, this information is not included in existing DSSs and appears in only a few studies [51-53], mainly without reference to internationally recognized classifications.

Furthermore, experts emphasized the importance of assessing the overall impact of MDs, and the literature indicates that the effects of introducing an MD may vary considerably across different health services [54,55]. Nevertheless, only one study specifies the health services an MD can enable [56], and a few more limit themselves to identifying the locations of use rather than detailing the enabled services [51,54,57].

Similarly, identifying the roles and activities of those operating the MDs within health care services would support understanding stakeholders’ responsibilities and needs, as well as assessing whether workflows should be modified or MDs adapted to fit existing processes. However, these aspects are rarely conceptualized [56], and existing DSS or databases do not provide information about the service in which the MD is used or the human resources involved.

Interviewees also emphasized the importance of accessing information related to manufacturers, as they envision using the system to search for MDs produced by specific manufacturers. Nevertheless, the manufacturer is explicitly specified in only 3 existing works [53,58,59], and none adopt unique manufacturer identifiers such as those required by EUDAMED [48].

Moreover, no study suggests using HTA reports as information sources, despite their rich, structured, and publicly available content. Most of the conceptual elements proposed in EUnetHTA, a framework officially recognized by the European Union and reportedly used by the interviewed experts, are not addressed in the reviewed conceptualizations.

Finally, specifying the MD application is also essential for MD evaluation [27], as it allows the identification of appropriate reference evaluation models. However, only one study addresses this aspect [60], and it does so without referring to existing nomenclatures.

### Phase 2 Results: Conceptual Map (First Draft)

The first draft of the conceptual map resulted from the Knowledge elicitation and Conceptualization phases.

Several properties are used to identify the MD: the Trade name with which the MD is sold on the market, the UDI-DI (ie, the unique numeric or alphanumeric code associated with all MDs placed on the European market), the EMDN code (ie, code of the MD according to the EMDN [61]), the Model of the MD, the Risk class of the MD, Scientific publications related to the MD, Technical sheets related to the MD, Patents related to the MD and its components (if any). Moreover, each MD is characterized by its Application (ie, whether it is used for diagnostic or screening reasons or medical and surgical interventions). Finally, each MD’s Type is specified (ie, whether it is a single component, a system, or a procedure pack [2]). The Manufacturer (ie, the company that produces the MD) is described by specifying its Name, the ID the manufacturer has received from the European Union, and the Country where the manufacturer is headquartered. The Medical Condition (ie, health conditions the MD is expected to treat that make it eligible to be used) is expressed as the intersection of the target user’s impairments, disease, and activity limitations—which can be described with the World Health Organization standards International Classification of Functioning, Disability, and Health (ICF) [62] and ICD-10 (International Statistical Classification of Diseases and Related Health Problems, Tenth Revision*)* [63]. The Health Service (ie, individual services provided to maintain or improve a person’s health, such as medical visits, diagnostic tests, treatments, surgeries, and rehabilitation services) is identified by the Name and 2 Nomenclature codes*,* one corresponding to the Major Diagnostic Categories and the other to the Diagnosis-Related Groups. The Major Diagnostic Categories represent 25 codes organizing diagnoses for all organs and systems [64], while the Diagnosis-Related Groups were developed to identify the health products a patient can receive from a health service provider [65]. Together, the 2 classifications are used in Italy at a national level for administrative purposes and to organize the different health services that can be provided to patients. For each health service, the Human resources (ie, clinical personnel, caregivers, or patients) performing it and the Setting (ie, the location where the human resources performing the health service are, which can be the clinical setting or the patient’s house) are specified. The representation of the HTA reports on the MD is included in the map. The impacts generated by the introduction of MDs on stakeholders depend on how the health services are designed to deploy them [66]. Therefore, each HTA report is associated with one or more health services involving one or more MDs. One health service involving a given MD could be evaluated in more than one HTA report. Some properties are included in the conceptual map to identify the HTA report: the Link to the HTA report, the ID of the report, the Date on which the report was published, and the report’s Authors. Considering the importance of the EUnetHTA [67,68], only HTA reports produced following it are included. The “Assessment Elements” conceptual item refers to the specific aspects evaluated in the report. Each assessment element is characterized by an ID and consists of the combination of a Domain, a Topic*,* and an Issue. The “Results” item corresponds to the results for a given assessment element that are provided in the HTA report. The “Method” item describes the method by which the assessment element’s results have been obtained. Starting from the list of assessment elements in the EUnetHTA, the authors included only 23 elements in the map. Table S4 in Multimedia Appendix 2 describes the exclusion criteria that guided the selection of the assessment elements, while Table S5 in Multimedia Appendix 2 lists the assessment elements included in the map. Both tables were discussed with the domain experts who participated in the focus groups.

### Phase 3 Results: Conceptual Map (Finalized)

The conceptual map resulting from the previous phase was adopted as the basis for this phase, during which 5 focus groups with 23 domain experts were organized (refer to Table 1). An improved version of the conceptual map resulted from the Co-design phase. This phase consisted of 5 focus groups where 23 experts were asked to comment on a purposely populated map, referring to a real MD. All participants found that the conceptual items in the map were necessary for representing the domain, but they suggested that some properties should have been added to the map to better describe the MD:

1. Conditions of use: they represent the conditions under which the MD can be used safely and effectively according to the manufacturer’s specifications or best practices. The introduction of such an item was advocated, among others, by participant FG2-HTA, who pointed out that the conditions of use that manufacturers specify in technical sheets are essential to identify whether an MD can be chosen to address specific needs.
2. Clinical trials: they consist of the codes of clinical trials that are being or were performed on the MD. These data could be obtained from the available clinical trial databases, such as the ISRCTN registry [69]. Including these codes could support evidence-based decisions because, as FG5-Manufacturer1 put forward, they provide information on the safety, efficacy, and appropriateness of the MDs in various settings.
3. End of life: it represented the date when the MD would presumably be withdrawn from the market. As participant FG4-Manager2 stressed, this information is essential since it corresponds to the date until spare parts and maintenance services will be available.
4. Repeatability of usage; it refers to whether the MD is designed for single or multiple uses. Participant FG4-Clinician2 highlighted that while multiuse MDs can promote sustainability, they also present challenges related to cleaning and sterilization, which may adversely affect operational efficiency.
5. Market distribution: it identifies the countries where the MD is distributed, which is essential to determine whether an MD is available in a certain geographic area, as participant FG1-Clinician1 remarked.

**Figure 3.**
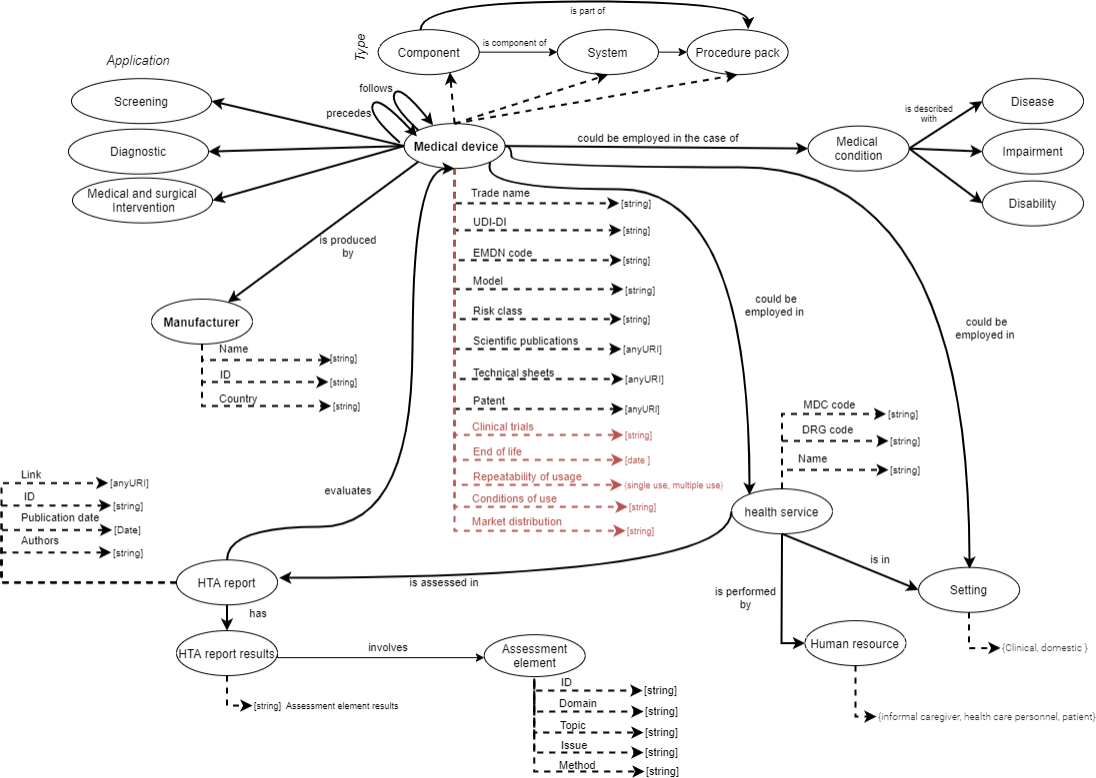
Phase 3: Conceptual map adopted as the basis for the ontology. HTA: Health Technology Assessment. DRG: Diagnosis-related groups, EMDN: European Medical Device Nomenclature. MDC: Major diagnostic categories.

Figure 3 shows the map; ovals correspond to classes, arrows correspond to relationships between classes, and properties are placed on dashed arrows related to the class they refer to. The conceptual items in red were added after Phase 3.

Table 2 shows the glossary of conceptual items included in the conceptual map, while Table 3 reports the list of competency questions. In both tables, the column “First (1) or second (2) draft” specifies whether the conceptual item and competency question were present in the first draft of the map or were added in the second draft, considering the comments received during focus groups.

**Table 2.** Phase 3: glossary of conceptual items included in the conceptual map (first and second draft).

ID	Description of the conceptual item
First draft	
Medical device	Medical device.
Trade name	Trade name with which the MD^a^ is sold on the market.
UDI-DI code	Unique numeric or alphanumeric code associated with all MDs placed on the European market.
EMDN^b^ code	Code of the MD according to the European Medical Device Nomenclature.
Model	Model of the MD.
Risk class	Risk class of the MD.
Scientific publication	Scientific publications related to the MD.
Technical sheets	Technical sheets related to the MD.
Patent	Patents related to the MD.
Type	Device, component, system, and procedure pack.
Application	Screening, medical and surgical intervention, and diagnosis.
Manufacturer	The company that produces the MD.
Manufacturer’s name	Name of the manufacturer.
Manufacturer’s ID	ID the manufacturer has received from the European Union.
Manufacturer’s country	Country where the manufacturer is headquartered.
Medical condition	Diseases and impairments described by the ICF^c^ and/or *ICD-10*^d^ codes
Health service	Individual services provided to maintain or improve a person’s health, such as medical visits, diagnostic tests, treatments, surgeries, and rehabilitation services.
Health service’s name	Name of the health service.
DRG code	Diagnosis-related groups code of the health service.
MDC code	Major diagnostic categories code of the health service.
Human resource	The person who performs the health service (informal caregiver or health care personnel or patient)
Setting	Location where the health service is performed (clinical or domestic)
HTA^e^ report	HTA report produced following the EUnetHTA^f^.
HTA report’s Link	Link to the HTA report.
HTA report’s ID	ID of the HTA report.
HTA report’s publication date	The date on which the HTA report was published.
HTA report’s Authors	Authors of the HTA report.
Assessment element	An element that is evaluated in the HTA report.
ID	A unique identifier assigned to the assessment element.
Domain	A broad category that groups assessment elements within the EUnetHTA.
Topic	Subjects that characterize assessment elements within the EUnetHTA. The same topic could characterize elements in multiple Domains.
Issue	Questions that need to be answered to evaluate the MD with regard to the assessment element.
Method	The method by which the assessment element’s results have been obtained.
Results	The results for a given assessment element that are provided in the HTA report.
Second draft	
Conditions of use	Conditions under which the MD can be used safely and effectively according to the manufacturer’s specifications or best practices.
Clinical trials	Codes of clinical trials that are being or were performed on the MD.
End of life	Date when the MD would presumably be withdrawn from the market.
Repeatability of usage	(single-use; multiple-use)
Market distribution	Countries where the MD is distributed.

a MD: medical device.

b EMDN: European Medical Device Nomenclature.

c ICF: International Classification of Functioning, Disability, and Health.

d ICD-10: *International Statistical Classification of Diseases and Related Health Problems, Tenth Revision.*

e HTA: Health Technology Assessment.

f EUnetHTA: European Network for Health Technology Assessment.

**Table 3.** Phase 3: competency questions defining the scope of the knowledge represented in the conceptual map (first and second draft).

ID	Competency questions
First draft	
CQ1	How is an MD^a^ identified? ANSWER: An MD is identified with its commercial name, model name, nomenclature code, UDI-DI code, and risk class (according to EU normative); it can be composed of one or more components. Each MD is classified according to at least one of the applications (screening, medical and surgical intervention, and diagnosis).
CQ2	What are the accessible technical and scientific materials on the MD? ANSWER: An MD can have scientific publications describing it, technical sheets explaining (part of) its functioning, and patents.
CQ3	In what medical conditions can the MD be used? ANSWER: Medical conditions in which the MD can be used are described by the ICF^b^ and/or ICD-10*^c^* codes, representing the diseases and the impairments characterizing the medical condition.
CQ4	Is the MD a single-component MD, a system, or a procedure pack? ANSWER: An MD can comprise a single component, a system (ie, a combination of products that are intended to be interconnected or combined to achieve a specific medical purpose), or a procedure pack (a combination of products packaged together and placed on the market with the purpose of being used for a specific medical purpose).
CQ5	What is the MD’s manufacturer? ANSWER: The MD manufacturer is a company that produces the MD, which is identified via a name, an ID, and the countries where it is headquartered.
CQ6	How is MD versioning managed? ANSWER: MD can be preceded by prior versions and followed by new versions, each characterized as an MD.
CQ7	Which health services involve the MD? ANSWER: The health services in which the MD is adopted are services provided to patients characterized by a name, DRG^d^ code, and MDC^e^ code.
CQ8	What are the human resources that perform the health service? ANSWER: The human resources performing the health service may be the patient’s informal caregiver, a member of the health care personnel, or the patient.
CQ9	In what settings can the MD be used? ANSWER: The setting can be the patient’s house or domestic environment or the clinical setting (clinic, hospital, and so on).
CQ10	In what setting is the human resource performing the health service? ANSWER: The setting can be the domestic setting (eg, patient’s house) or the clinical setting (eg, clinic, hospital, and so on) and has to be included among the settings in which the MD can be used.
CQ11	Which HTA reports analyze a certain health service using a certain MD? ANSWER: The HTA^f^ reports analyzing a health service are identified with the ID, the URL of the HTA report, its year of publication, and the authors’ list; each report is associated with one or more MDs used in one or more health services.
CQ12	Which assessment elements are evaluated in the HTA report, and how are they characterized? ANSWER: The assessment elements included in a report are identified with the ID, the domain, a topic, an issue, and the method by which it can be assessed.
CQ13	What is the result of the assessment element in the HTA report? ANSWER: The result of an assessment element in the HTA report is an individual reporting written results.
Second draft	
CQ14	In what conditions can the MD be used?
CQ15	What clinical trials are being or have been conducted on the MD?
CQ16	What is the end of life of the MD?
CQ17	Is the MD single-use or multiuse?
CQ18	In which countries is the MD sold?

a MD: medical device.

b ICF: International Classification of Functioning, Disability, and Health.

c ICD-10: *International Statistical Classification of Diseases and Related Health Problems, 10th Revision*.

d DRG: diagnosis-related group.

e MDC: major diagnostic category.

f HTA: Health Technology Assessment.

As elicited from focus groups, the health services in which the MD could be used are gathered from HTA reports concerning the MD. The properties referring to classes in the map could be obtained from HTA reports, scientific publications, or technical sheets concerning the MD and the data on EUDAMED [48] (if available).

During one of the focus groups, a participant (FG3-Clinician2) pointed out that the applications of MD (screening, diagnosis, and medical and surgical interventions) should not be disjointed since the same MD could be used for more than one application. The suggestion demonstrates that focus groups could stimulate a high level of collaboration among experts and ontologists [21,35].

### Phase 4 Results: Prototype of the Domain Ontology

The ontology development phase adopted a modular approach—that is, each of the domains composing the prototype is modeled on a separate ontology [70]. The modules are imported into the general box and are developed using the Resource Description Framework and the Web Ontology Language representations of the conceptual map resulting from Phase 3. Thus, the ontology is organized into 5 modules (HealthService, MedicalCondition, HumanResources, and HTA modules, imported into a general box named MedDev, in which rules are specified). The ontology includes a total of 1432 classes, 32 object properties, 28 data-type properties, 118 individuals, and 64 rules. The ontology foresees the possibility of indicating whether an MD has a previous or subsequent version by reusing the sequence Ontology Design Pattern [71].

The prototypical ontology is available in Multimedia Appendix 1.

**Figure 4.**
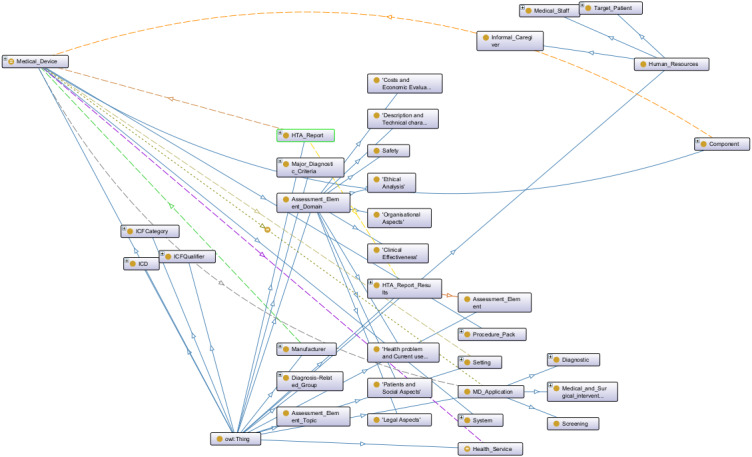
Ontograph obtained through Protégé. HTA: Health Technology Assessment; ICD-10: International Statistical Classification of Diseases and Related Health Problems, Tenth *Revision*; ICF: International Classification of Functioning, Disability, and Health.

Figure 4 shows the ontograph generated using Protégé, which provides a graphical representation of the ontology structure. In the ontograph, nodes represent ontology classes, while arrows denote semantic relationships between them. Solid blue arrows indicate explicitly asserted relationships defined in the ontology, whereas dashed arrows represent inferred relations generated by the reasoning process. Different arrow colors correspond to different object properties connecting the classes. Multimedia Appendix 3 comprises a figure (named “Modules composing the MedDev ontology”) that shows the graphical representation of the modules composing the MedDev ontology. In the figure, arrows indicate that a certain module is imported into another, and black rectangles specify reused ontological resources.

The ontology is instantiated with 6 MDs, whose information can be retrieved from 4 HTA reports. For example, 2 individuals, OTCA17 (Multimedia Appendix 4) and OTCA27 (Multimedia Appendix 5), belonging to the class HTA_Report assess GreenLight-XPS and compare it with Holmium_Laser_Therapeutic_Apparatus. The GreenLight-XPS is a system devoted to Medical_and_surgical_intervention which can be used for the ICD-10 disease N40.1 (benign prostatic hyperplasia with lower urinary tract symptoms) and for the ICF b610 (urinary excretory functions). This MD is adopted in Health_Services that involves MDC 12 “Diseases and disorders of the male reproductive system” and involves DRG 39 “benign prostatic hyperplasia” a health service performed by the human resource Surgeon.

Leveraging the Pellet reasoner [72], the ontology can represent the conceptual map to its full extent, enabling the representation of any MD and its characteristics, as well as its HTA reports (and results) characterizing it.

Furthermore, the semantic model of the ontology can answer the main questions stakeholders might raise when gathering information about MDs (refer to Table 3 reporting the competency questions). For example, leveraging the SPARQL query language, they could ask the ontology to retrieve all the MDs that treat a specific medical condition or ask for the single-use MDs that could be used in a certain health service. Similarly, it is possible to run a SPARQL query to search for specific strings within the HTA reports’ textual results (which are also reported using a specific data-type property; Textbox 1):

Textbox 1. **SPARQL query to search for specific strings within the Health Technology Assessment reports.**

SELECT DISTINCT ?MD ?report WHERE {

?MD a dev:Medical_Device .

?report a hta:HTA_Report ;

dev:investigatesMD ?MD ;

dev:hasReportResults ?results .

?results hta:assessElemResults ?resDesc .

FILTER (isLiteral(?resDesc) &&

regex(str(?resDesc),hospital”))

}

### Phase 5 Results: Feedback Obtained Through Ontology Validation

To assess whether a DSS based on the proposed ontology can meet the information needs that stakeholders typically have when comparing, evaluating, or introducing MDs, 13 stakeholders from 3 European countries were interviewed and surveyed (refer to Table 1). During the interviews, one of the authors presented examples, taken from the six MDs used to populate the ontology, to illustrate the types of knowledge represented within the proposed ontology. During and after the presentation of the examples, interviewees were invited to share their impressions and comments. Afterward, they were asked to complete a questionnaire composed of 13 Likert-scale questions and 2 open-ended questions, which allowed participants to elaborate on the strengths and weaknesses of the ontology.

Table S6 in Multimedia Appendix 2 reports the quantitative results of the questionnaire. The results show consistently high mean values with low variability, indicating overall positive expert feedback. Participants generally perceived that a DSS based on the proposed ontology may be useful, relevant, and well-aligned with their work tasks. The data also suggests a strong intention to use the system in the future.

Moreover, qualitative feedback was collected both during the presentation of the examples and through the open-ended questions in the questionnaire. Positive feedback primarily highlighted the system’s comprehensiveness, modular structure, interconnectivity, and usefulness in aggregating and linking information from different sources. Negative comments focused on the absence of cost-related information, the need for integration with national or regional databases, and the initial learning effort required to use the system effectively.

Overall, these findings confirm that the ontology can effectively support users in analyzing, selecting, and introducing new MDs within their organizations by satisfying the experts’ informational requirements.

### Examples of Use

This paragraph provides a nonexhaustive list of examples of how the ontology-based DSS could assist stakeholders in deciding whether and how to introduce MDs in real settings. The relevance of these applications emerged during the interviews and focus groups carried out during this study (Phases 1, 3, and 5).

As highlighted by VAL2-Manager, clinicians might use the DSS to retrieve information about a specific MD they have already selected. By consulting scientific publications, existing HTA evaluations, and available clinical trials on the MD, clinicians can assess whether it meets their needs before proposing it for purchase. Scientific publications may provide insights into how other organizations use the MD, while HTA evaluations and the final or preliminary results of clinical trials can further inform them of its potential impact.

Similarly, health care managers can use the DSS to explore information on selected MDs. They can access data on the medical conditions in which the MD can be used, identify the target population, and possibly realize that introducing the MD could modify care pathways for other conditions beyond those initially considered. Managers can also review technical sheets and patents to understand an MD’s functionality, application, and usage conditions, as well as check its availability, maintenance duration, and spare parts. As specified by VAL1-HTA and VAL3-Manager, such information helps assess organizational readiness, required adaptations, and regulatory compliance. For instance, they might discover that patient training is required or that the MD could be used in multiple health services, optimizing resource allocation.

The DSS also enables comparisons between MDs for a given medical condition. As highlighted by VAL6-Manager, this helps stakeholders identify the most suitable options to improve clinical pathways.

VAL5-Manager noted that managers can also search for MDs from specific manufacturers based on past positive purchase experiences.

Finally, as specified by VAL10-HTA, for MDs with minimal organizational and economic impact, typically not subjected to a comprehensive HTA analysis, the DSS alone might provide sufficient information to support decisions on whether to introduce a given MD.

## Discussion

### Main Findings

This study aimed to develop a comprehensive, evidence-based ontology to support the informed adoption of MDs in health care organizations. The authors successfully designed a modular ontology serving as the backbone of a DSS, capturing key elements to inform adoption decisions. It integrates conceptual components such as medical conditions, health services enabled by MDs, professional roles and activities, manufacturers, HTA report data, and MD applications, often absent in existing systems and prior literature (see the “PRISMA Literature Review” section). At the same time, it deliberately excludes components irrelevant to the decision problem (eg, hardware and software infrastructure), achieving a balance between granularity, conciseness, and comprehensiveness. The ontology is grounded in established international sources [61-63] and national nomenclature codes to identify the health service [64,65].

The ontology was developed through a custom knowledge-based methodology that integrates focus groups to elicit knowledge. Our approach builds on existing ontology engineering practices [19] and draws on AgiSCOnt principles [20,21], using conceptual maps to formalize complex, interdisciplinary domains. The ontology’s instantiability was validated by populating it with data from 4 HTA reports, confirming both its robustness and practical applicability. Furthermore, the validation phase demonstrated that the ontology effectively addresses the core information needs of stakeholders.

### Main Findings: Discussion and Comparison to Existing Literature

The ontology presented in this study represents, to the best of our knowledge, the first comprehensive, generalizable, and evidence-based conceptualization developed specifically to support informed adoption decisions for MDs. It fills key gaps in existing literature and DSSs by capturing the full spectrum of stakeholders’ knowledge needs. Only a limited number of existing conceptualizations consider the MD domain holistically, and key elements highlighted by expert interviews, such as the identification of medical conditions for MD use, the roles and activities of health care professionals, service-level impact, manufacturer-specific information, HTA reports, and MD applications, are either missing or incompletely addressed in prior works.

Beyond its conceptual coverage, the robustness of the proposed ontology is rooted in the knowledge-based and collaborative ontology engineering methodology adopted. Given the complexity and multidisciplinary nature of the MD adoption domain, traditional ontology engineering approaches are often insufficient to capture implicit knowledge. By integrating focus groups into the knowledge acquisition and conceptualization phases, this study empirically validates their effectiveness in supporting the cocreation of reliable ontological models, enabling the systematic elicitation and formalization of heterogeneous expert perspectives [25].

Grounded in established sources, the proposed ontology ensures interoperability across health care settings. Moreover, the ontology engineering methodology used by the authors followed and the ontology’s modular design ensure both generalizability and practical applicability across diverse contexts: the ontology’s core conceptual structure captures regulatory, organizational, and assessment processes that are widely shared across health care systems, and modules can be easily refined or extended to align with local practices.

### Limitations

Four key issues remain open for further development of the domain ontology of MDs.

First, most experts involved in this study were from public and private service providers in Italy, and the conceptual item Health Service was modeled using 2 Italian nomenclature codes, as no international standard currently exists. Involving mainly Italian experts in the ontology development does not compromise the conceptual soundness of the ontology at the structural level, and the core classes and relations were defined to capture regulatory, organizational, and assessment processes that are common across European health care systems. However, the use of Italian nomenclatures affects specific implementation-level components (ie, the health service conceptual item). Second, the HTA module is based on the EUnetHTA model (which is the one the European Union has recognized as the official model for HTA reports and is currently the most adopted framework for HTA in Europe), but other relevant models could be used to create HTA reports, such as models developed specifically for hospitals [73]. Third, the effort required to populate the ontology is substantial, and the low quality and limited availability of certain knowledge sources (eg, HTA reports, technical sheets, and so on) could pose a challenge for populating it. Fourth, while the ontology has been validated in terms of conceptual completeness, consistency, and instantiability, its full clinical and financial decision-making value can only be demonstrated through the implementation and pilot testing of a functioning ontology-based DSS.

### Conclusions: Theoretical Contributions, Managerial Contributions, and Future Research Directions

This paper proposes a validated ontology that aims to support the informed adoption of MDs in health care organizations. While prior literature has mostly focused on partial conceptualizations and existing databases cover only limited aspects of MDs, the proposed ontology fills this gap by providing a comprehensive conceptualization that addresses all the key information needs of stakeholders involved in the decision problem. The ontology’s ability to capture this full spectrum of information stems from the custom, collaborative, knowledge-based ontology engineering methodology adopted in its development. This methodological choice enabled the elicitation and integration of diverse forms of expert knowledge, ensuring that the resulting conceptual model is both robust and closely aligned with stakeholders’ needs. Thus, this study demonstrates the value of cocreating conceptualizations with stakeholders when dealing with complex and multidisciplinary domains and provides useful guidance for future efforts aimed at conceptualizing complex domains characterized by multiple stakeholders with heterogeneous information needs. This collaborative methodology supports the abstraction of locally elicited expertise into higher-level conceptual models, thereby enhancing the transferability of the resulting ontology beyond the specific national context in which it was developed.

From a managerial perspective, the ontology provides a structured, knowledge-driven foundation to support decision-making in the adoption of MDs. By integrating heterogeneous sources of explicit and implicit knowledge, it enables stakeholders to systematically compare MDs and select those best suited to their specific contexts. The ontology can guide structured evaluation and integration processes, ensuring that decisions are evidence-informed, transparent, and reproducible. A promising and still largely unexplored field of application could be oral and dental health care [74], a domain characterized by rapid innovation that requires informed and well-structured adoption processes. This approach can help health care organizations streamline MDs’ introduction, enhance clinical workflows, and reduce the underuse of innovations. The modular design allows managers to adapt components, such as health service nomenclatures or HTA modules, to local practices without altering the overarching conceptual structure, facilitating implementation across diverse health care systems. The purpose of this study is not to provide a system to be officially adopted at the European level, nor does it have the institutional mandate or resources to do so. Rather, its managerial value lies in illustrating how to rationalize expert knowledge and identify the key informational components necessary to support informed MD adoption decisions, thus offering a conceptual foundation for future research and development initiatives at the European level.

In light of the identified limitations and challenges, this study represents a call for the development of international projects aimed at establishing shared nomenclatures and replicating the knowledge acquisition process with stakeholders from other European health care systems. The ontology’s modular design allows the HTA module, based on the EUnetHTA framework, to be replaced with alternative HTA models if needed, and the HumanResources module to be adapted to other health care systems’ nomenclatures without affecting the overall structure. This modularity ensures that updating and adapting the ontology remains straightforward. Future efforts may also focus on ontology population, which could be automated through ontology learning frameworks, provided that datasets such as EUDAMED or other sources become publicly available. In this regard, the EU Regulation 2021/2282 on HTA may facilitate the creation of higher-quality knowledge sources, supporting ontology population. Finally, integrating the ontology into a DSS and evaluating its clinical and economic impact via real-world pilot studies involving multiple health care organizations is essential for full validation. Engaging experts and stakeholders from different countries in these pilots will further verify the ontology’s applicability across diverse health care contexts.

## Supplementary material

10.2196/88366 Multimedia Appendix 1 Zipped folder containing the MedDev ontology.

10.2196/88366 Multimedia Appendix 2 Additional tables.

10.2196/88366 Multimedia Appendix 3 Modules composing the MedDev ontology.

10.2196/88366 Multimedia Appendix 4 Lithium triborate laser for photoselective vaporisation of the prostate in the treatment of benign prostatic hyperplasia.

10.2196/88366 Multimedia Appendix 5 Comparative effectiveness of surgical techniques and devices for the treatment of benign prostatic hyperplasia.

10.2196/88366 Checklist 1 PRISMA checklist.

10.2196/88366 Checklist 2 Qualitative research checklist.

## Abbreviations

DSS: Decision Support Systems
EMDN: European Medical Device Nomenclature
EUDAMED: European Database of Medical Devices
EUnetHTA: European Network for Health Technology Assessment
HTA: Health Technology Assessment
ICD-10: International Statistical Classification of Diseases and Related Health Problems, Tenth Revision
ICF: International Classification of Functioning, Disability, and Health
MD: medical device
PRISMA: Preferred Reporting Items for Systematic Reviews and Meta-Analyses
Q: question
