# Supplementary material for: An Ontology for the Adoption of Medical Devices in Health Care Organizations: Design and Development Study
Source: J Med Internet Res. 2026 Jul 10;28:e88366. doi: 10.2196/88366 (PMC13353408; doi:10.2196/88366)
Supplement: Multimedia Appendix 2 [file jmir-v28-e88366-s002.docx]

**Table S1.** Phase 1 - Analysis of the **taxonomies** of MDs from the included articles.

| **Article** | **Purpose (Q2)** | **Intended users (Q3)** | **Domain (Q4)** | **Conceptual items (first-order categories) (Q4a)** | **Sources**  **(Q4b)** | **Appl.**  **(Q5)** |
| --- | --- | --- | --- | --- | --- | --- |
| [1] | Support in designing user-centered MDs | Clinicians, manufacturers | Pervasive systems | Body functions, activity limitations and participation restrictions of MD users, potential beneficiaries, source of data, target of actuator's action, environment where the MD is used, Products and technology. | [2] | no |
| [3] | Classify MDs | not specified | Portable MDs designed for the point-of-care environment, not implantable, including at least one output interface. | Type of MD, output interface types, and embedded security measures. | [4] | no |
| [5] | Support HTA assessments | Policymakers, HTA analysts | All | Risk categories, reimbursement characteristics. | [6–10] | no |
| [11] | Investigate MDs’ functionalities | not specified | Mobile applications for healthy nutrition and physical activity available on the Google Play Store. | Purpose, feature. | no | no |
| [12] | Classify MDs | not specified | Brain-Computer Interface systems | Type of brain-computer interface the MD is based on. | no | no |
| [13] | Classify MDs | not specified | Caregiver robots | Application, integration into smart environments, human-robot interaction, and the target audience. | no | no |
| [14] | Classify MDs | Researchers | All | MD Type. | no | no |
| [15] | Classify MDs | Patients, caregivers, and clinicians | MDs based on the Internet of Things for dementia | Type of dementia, variable of interest, type of participant involved in the evaluation, main objective, targeted users, actuator type, method to evaluate the system, origin of the data, MD type, deployment location, machine learning approach. | no | no |
| [16] | Classify MDs | Clinicians, manufacturers | Implantable MDs | MDs identification ("Manufacturer, product code, Unique Device Identification, lot number"), Type, and Technical and functional features. | [17, 18] | yes |
| [19] | Support new service design and MD selection | Clinicians, managers, and manufacturers | All | Technology, target disease, impacted tasks, stakeholders, service phases, setting, automation strategy. | [20] | no |
| [21] | Taxonomize MDs | Authorities, manufacturers, and HTA bodies | Digital MDs | Category (Inform, Diagnose, Manage, Monitor, and Treat), function, intended beneficiary, and context of use (stand-alone or integrated care pathway). | [22, 23], and national classifications | no |
| [24] | Understand the strengths and limitations of the data MDs collect. | Health workforce | MDs collecting Electronic Health Data | Category (MDs used by healthcare organizations, MDs used by consumers/community; MDs that passively collect data) | no | no |
| [25] | Clarify the current use of artificial intelligence in MDs | Clinicians, policymakers, and manufacturers | MDs based on artificial intelligence authorized by the American Food and Drug Administration | Data Type (Images, Signals, Omics data, and Tabular Electronic Health Records), Clinical Function (Assessment, Intervention), Function (Analysis, Generation, or both). | no | no |

**Table S2.** Phase 1 - Analysis of the ontologies and conceptual map (identified with an asterisk) of MDs from the included articles.

| **Article** | **Purpose (Q2)** | **Intended users (Q3)** | **Domain (Q4)** | **Conceptual items (class [properties])**  **(Q4a)** | **Sources**  **(Q4b)** | **Appl**  **(Q5)** |
| --- | --- | --- | --- | --- | --- | --- |
| [26]* | Address the challenges in designing, developing, implementing, using, and evaluating MDs | not specified | Health information technologies | Hardware and software infrastructure; Clinical content; Human-computer interface; People, Workflow, and communication; Internal Organizational Policies, Procedures, and Culture; External rules, regulations, and pressures; System Measurement and Monitoring. | no | no |
| [27] | Map the possible context of use for MDs | Clinicians and managers | Interactive MDs and medical software applications | Devices, widgets [Audio, Haptic, or Visual widgets], situational factors, locations | no | no |
| [28] | Support in designing, comparing, and selecting MDs | Manufacturers, Clinicians | Robots for physical rehabilitation | Rehabilitation robots [name, active degree-of-freedom, passive degree-of-freedom, control modes, disorder level, functionality, interaction type, intervention time, kinematic type, motion capability, targeted population, targeted disorder], joint movements [range of motion type, minimum range of motion, maximum range of motion, actuation, transmission, backdrivability, backdrivability type], owners [Name, mail, institution], references [title, author, clinical study, year, published at, URL], assessments. | no | yes |
| [29] |  |  |  |  |  |  |
| [30] | Describe the functional attributes of MDs | Clinicians | Cardiac implantable MDs | Type, Functions, and Patients. | [31] | partial |
| [32] | Ensure Semantic Interoperability between hospital information systems. | Manufacturers | All | MD [description, nomenclature code, risk, date of manufacturing, ID required to order the MD, CE marking, units of quantity used for the MD, standard it has to be adhered to], legal procedure [Name, underlying legal basis of the procedure, Result of the legal procedure], operator [ID, name, address], manufacturer [ID, name, address], selected institution [ID, date of renewal, task of the selected institution] | [8–10] | no |
| [33] | Define MDs from a technical perspective. | Manufacturers, computer scientists | Hearing aids | Types, Configurations, Conduction, Target, Functionalities, Technology, Loss severity. | no | no |
| [34] | Support in selecting MDs | Clinicians and patients | Wearable MDs | MDs [actuators, battery life, communication With Smartphone, Manufacturer, advantages and disadvantages of the MDs (as textual notes), the diseases and symptoms monitored, obtrusive (true/false), body position, URL of the web page describing the sensor] | [35] | partial |
| [36] | Formalize the metadata about MDs for health data analytics | Computer scientists | Wearable MDs | Activity Recognition model, Monitored Person, Sensors and Actuators that produce data, the wearables and sensors that can be attached to or near a monitored person, and caregivers. | [37–39] | yes |
| [40] | Identify, analyze, and manage vulnerabilities in MDs | IT Specialists | Internet of Medical Things | Type, Commercial and technical information, Firmware/Software, and security weaknesses. | National Databases | yes |

**Table S3.** Phase 1 - EUnetHTA's assessment elements addressed in the included works.

| **Domain** | **Topic** | **ID** | **Issue** | **Works** **addressing this element** |
| --- | --- | --- | --- | --- |
| Health problem and current use of technology | Utilisation | E0001 | For which health conditions and populations, and for what purposes is the technology used? | [1, 11, 13, 15, 19, 21, 28, 33, 34] |
|  | Target Population | A0007 | What is the target population in this assessment? | [15, 21] |
|  | Target Condition | A0009 | What aspects of the consequences/burden of disease are targeted by the technology? | [1, 13, 19, 28] |
|  | Regulatory Status | A0021 | What is the reimbursement status of the technology? | [5] |
| Description and technical characteristics of technology | Other | A0022 | Who manufactures the technology? | [16, 32, 34] |
|  | Features of the technology | B0001 | What is this technology and the comparator(s)? | [3, 11, 16, 27–30, 32, 36] |
|  |  | B0004 | Who administers the technology and the comparators, and in what context and level of care are they provided? | [19, 26, 32] |
|  | Investments and tools required to use the technology | B0009 | What equipment and supplies are needed to use the technology and the comparator? | [26] |
|  | Patient safety | C0008 | How safe is the technology in relation to the comparator(s)? | [5] |
|  | Morbidity | D0005 | How does the technology affect symptoms and findings (severity, frequency) of the disease or health condition? | [26, 29, 34] |
|  | Function | D0011 | What is the effect of the technology on patients' body functions? | [29, 34] |
|  | Benefit-harm balance | D0029 | What are the overall benefits and harms of the technology in health outcomes? | [26] |
| Costs and economic evaluation | Resource utilisation | E0001 | What types of resources are used when delivering the assessed technology and its comparators (resource-use identification)? | [32] |
| Ethical analysis | Benefit-harm balance | F0003 | Are there any other hidden or unintended consequences of the technology and its applications for patients, relatives, other patients, organisations, commercial entities, society etc.? | [26] |
|  | Autonomy | F0007 | Does the implementation or withdrawal of the technology challenge or change professional values, ethics or traditional roles? | [19] |
|  | Respect for persons | F0101 | Does the technology invade the sphere of privacy of the patient/user? | [3] |
| Organisational aspects | Health delivery process | G0001 | How does the technology affect the current work processes? | [19] |
|  |  | G0002 | What kind of involvement has to be mobilized for patients/participants and important others and/or caregivers? | [19] |
|  |  | G0012 | In what way is the quality assurance and monitoring system of the new technology organised? | [26] |
|  | Culture | G0011 | How are the other interest groups taken into account in the planning/implementation of the technology? | [26] |
|  | Privacy of the patient | I0007 | Is there a possibility that the use of the technology produces additional information that is not directly related to the current care of the patient and may violate their right to respect for privacy? | [3] |
|  |  | I0009 | What do laws/binding rules require with regard to appropriate measures for securing patient data and how should this be addressed when implementing the technology? | [3] |
|  | Regulation of the market | I0024 | What kind of regulation exists for the acquisition and use of the technology? | [32] |
|  |  | I0025 | What legal restrictions are there for marketing the technology to the patients? | [32] |

**Table S4.** Phase 2 - Exclusion criteria that guided the selection of the assessment elements to include in the conceptual map.

| **Exclusion criterion** | **Description** | **Motivation of the criterion** |
| --- | --- | --- |
| *Context-dependent* | The results of the assessment element depend on the service put in place by the organization at the time of the analysis. | HTA evaluations compare the value of the current service (without the MD) to that of services including the MD, to assess if the investment improves the overall value offered to stakeholders. Therefore, the results of some elements depend on the service put in place by the organization at the time of the analysis. The results of these elements could not be useful for hypothetical stakeholders involved in adoption decisions because they are context-dependent. |
| *Regulatory Status* | The element describes the development and regulatory status of the MDs. | Only CE-marked MDs are included. |
| *Comparison* | The element compares different MDs and/or alternative ways of employing an MD based on other assessment elements. | Users will autonomously compare the performance of alternative MDs and/or of the same MD employed in different health services by looking at other assessment elements and navigating the ontology. |
| *Non-independent* | The element is not independent of other elements. | Assessment elements are excluded if they are the means to impact or are strictly related to other elements in the EUnetHTA. |
| *Out of scope* | The element reports problems or obstacles that those who have conducted the analysis had to overcome. | Expert knowledge has already been produced. The decision-makers are interested in the results and not in the problems or obstacles that those who have conducted the analysis had to overcome. |

**Table S5.** Phase 2 - Assessment elements included in the conceptual map.

| **Domain** | **Topic** | **ID** | **Issue** |
| --- | --- | --- | --- |
| Description and technical characteristics of technology | Investments and tools required to use the technology | B0007 | What material investments are needed to use the technology? |
|  |  | B0008 | What kind of special premises are needed to use the technology and the comparator(s)? |
|  |  | B0009 | What equipment and supplies are needed to use the technology and the comparator? |
|  |  | B0010 | What kind of data/records and/or registry is needed to monitor the use of the technology and the comparator? |
|  | Training and information needed to use the technology | B0013 | What kinds of skills and training characteristics and information are needed for the personnel/caregivers using this technology? |
| Safety | Patient safety | C0002 | Are there harms related to dosage or frequency of applying the technology? |
| Clinical  Effectiveness | Mortality | D0001 | What is the expected beneficial effect of the technology on mortality? |
|  | Morbidity | D0005 | How does the technology affect symptoms and findings (severity, frequency) of the disease or health condition? |
|  | Morbidity | D0006 | How does the technology affect progression (or recurrence) of the disease or health condition? |
|  | Change-in management | D0010 | How does the technology modify the need for hospitalisation? |
|  | Function | D0011 | What is the effect of the technology on patients' body functions? |
|  | Health-related Quality of Life | D0012 | What is the effect of the technology on generic health-related quality of life? |
|  | Health-related Quality of Life | D0013 | What is the effect of the technology on disease-specific quality of life? |
|  | Function | D0014 | What is the effect of the technology on work ability? |
|  | Function | D0015 | What is the effect of the technology on return to previous living conditions? |
|  | Function | D0016 | How does the use of the technology affect activities of daily living? |
|  | Quality of life | D0030 | Does the knowledge of the test result affect the patient's non-health-related quality of life? |
|  | Test accuracy | D1001 | What is the accuracy of the test against reference standard? |
| Ethical analysis | Benefit-harm balance | F0003 | Are there any other hidden or unintended consequences of the technology and its applications for patients, relatives, other patients, organisations, commercial entities, society etc.? |
|  | Autonomy | F0004 | Does the implementation or use of the technology affect the patient's capability and possibility to exercise autonomy? |
|  | Respect for persons | F0008 | Does the implementation or use of the technology affect human dignity? |
|  | Respect for persons | F0009 | Does the implementation or use of the technology affect the patient's moral, religious or cultural integrity? |
|  | Respect for persons | F0101 | Does the technology invade the sphere of privacy of the patient/user? |

**Table S6.** Phase 5 - Results of the questionnaire administered to validate the ontology.

| **Questions** | **Mean** | **SD** |
| --- | --- | --- |
| Using the system improves my performance in my job. | 3.71 | 0.96 |
| Using the system in my job increases my productivity | 3.79 | 0.67 |
| Using the system enhances my effectiveness in my job. | 4.00 | 0.65 |
| I find the system to be useful in my job. | 4.29 | 0.80 |
| In my job, the usage of the system is important. | 4.00 | 1.00 |
| In my job, the usage of the system is relevant. | 3.93 | 0.80 |
| The use of the system is pertinent to my various job-related tasks. | 3.86 | 0.52 |
| Assuming I have access to the system, I intend to use it. | 4.07 | 0.70 |
| Given that I had access to the system, I predict that I would use it. | 4.21 | 0.41 |
| I plan to use the system in the future. | 3.86 | 0.64 |
| The set of information the system can provide is relevant for my job. | 4.21 | 0.41 |
| I believe the system adequately meets my needs when performing my job. | 3.93 | 0.70 |
| The information available in the system fully addresses the questions I have while performing my job. | 3.86 | 0.83 |

# References

1. Muras JA, Cahill V, Stokes EK. A taxonomy of pervasive healthcare systems. 2006 Pervasive Health Conference and Workshops, PervasiveHealth. 2006;:1–10. https://doi.org/10.1109/PCTHEALTH.2006.361680.
2. World Health Organization. International Classification of Functioning, Disability, and Health (ICF). 2024. https://www.who.int/standards/classifications/international-classification-of-functioning-disability-and-health. Accessed 9 Oct 2024.
3. Velez D, Shanblatt M. Taxonomy of current medical devices for POCT applications and the potential acceptance of Bluetooth technology for secure interoperable applications. 2011 IEEE 13th International Conference on e-Health Networking, Applications and Services, HEALTHCOM 2011. 2011;:288–95. https://doi.org/10.1109/HEALTH.2011.6026767.
4. IEEE/ISO 11073-20101-2004, Health informatics — Point‑of‑care medical device communication — Part 20101: Application profiles — Base standard. 2004.
5. Henschke C, Panteli D, Perleth M, Busse R. Taxonomy of medical devices in the logic of health technology assessment. Int J Technol Assess Health Care. 2016;31:324–30. https://doi.org/10.1017/S0266462315000562.
6. OECD/WHO/Eurostat. A System of Health Accounts: 2011 Edition. Paris: OECD Publishing; 2011. https://doi.org/10.1787/9789264116016-en.
7. Anand K, Veermaram C, Saini SK, Singh BK. Global Medical Device Nomenclature: The Concept for Reducing Device-Related Medical Errors. Journal of Young Pharmacists. 2010;2:403–9. https://doi.org/10.4103/0975-1483.71637.
8. Council Directive 90/385/EEC of 20 June 1990 on the approximation of the laws of the Member States relating to active implantable medical devices, Official Journal L 189, 20.7.1990, pp. 17–37. :17–37.
9. Council Directive 93/42/EEC of 14 June 1993 concerning medical devices, Official Journal L 169, 12.7.1993, pp. 1–43.
10. Directive 98/79/EC of the European Parliament and of the Council of 27 October 1998 on in vitro diagnostic medical devices, Official Journal L 331, 7.12.1998, pp. 1–37.
11. Villasana MV, Pires IM, Sá J, Garcia NM, Zdravevski E, Chorbev I, et al. Mobile Applications for the Promotion and Support of Healthy Nutrition and Physical Activity Habits: A Systematic Review, Extraction of Features and Taxonomy Proposal. Open Bioinforma J. 2020;12:50–71. https://doi.org/10.2174/1875036201912010050.
12. Wang Y, Nakanishi M, Zhang D. EEG-Based Brain-Computer Interfaces. In: X. Zheng, editor. Neural Interface: Frontiers and Applications. Advances in Experimental Medicine and Biology, vol 1101. Singapore: Springer; 2019. p. 41–65. https://doi.org/10.1007/978-981-13-2050-7_2.
13. Santos NB, Bavaresco RS, Tavares JERR, Ramos GDO, Barbosa JLV V. A systematic mapping study of robotics in human care. Rob Auton Syst. 2021;144:1–24. https://doi.org/10.1016/j.robot.2021.103833.
14. Chaudhary S, Kakkar R, Jadav NK, Nair A, Gupta R, Tanwar S, et al. A Taxonomy on Smart Healthcare Technologies: Security Framework, Case Study, and Future Directions. J Sens. 2022;2022:1–30. https://doi.org/10.1155/2022/1863838.
15. Esquer-Rochin M, Rodríguez L-F, Gutierrez-Garcia JO. The Internet of Things in dementia: A systematic review. Internet of Things. 2023;22:100824. https://doi.org/10.1016/j.iot.2023.100824.
16. Pascucci S, Franzò M, Mari V, Biondi A, Ceccarelli S, Bini F, et al. Key points to design the MDs Library of a National implant Registry: lesson learned from the Italian experience. Convegno Nazionale di Bioingegneria. 2023;:1–4.
17. Italian Ministry of Health. National Classification of Medical Devices (CND). 2023. https://www.salute.gov.it/portale/dispositiviMedici/dettaglioContenutiDispositiviMedici.jsp?lingua=italiano&id=328&area=dispositivi-medici&menu=registrazione. Accessed 15 Feb 2024.
18. Racchi M, Govoni S, Lucchelli A, Capone L, Giovagnoni E. Insights into the definition of terms in European medical device regulation. Expert Rev Med Devices. 2016;13:907–17. https://doi.org/10.1080/17434440.2016.1224644.
19. Vannelli S, Visintin F. A Process-Based Taxonomy of Medical Devices for Clinical Pathways Design and Innovation. In: Borgianni Y, Matt DT, Molinaro M, Orzes G, editors. Towards a Smart, Resilient and Sustainable Industry. ISIEA 2023. Lecture Notes in Networks and Systems, vol 745. Springer; 2023. p. 296–308. https://doi.org/10.1007/978-3-031-38274-1_25.
20. National Center for O*NET Development. O*Net Portal. 2024. https://www.onetonline.org/. Accessed 6 May 2024.
21. Boers M, Rochereau A, Stuwe L, Miguel LS, Klucken J, Mezei F, et al. Classification grid and evidence matrix for evaluating digital medical devices under the European union landscape. NPJ Digit Med. 2025;8:304. https://doi.org/10.1038/s41746-025-01697-w.
22. European Parliament. Regulation 2017/745 on medical devices. 2017.
23. Assessment E. EUnetHTA Joint Action 3 WP4. 2018; February:1–500.
24. Janssen A, Donnelly C, Shaw T. A Taxonomy for Health Information Systems. J Med Internet Res. 2024;26:e47682. https://doi.org/10.2196/47682.
25. Singh R, Bapna M, Diab AR, Ruiz ES, Lotter W. How AI is used in FDA-authorized medical devices: a taxonomy across 1,016 authorizations. NPJ Digit Med. 2025;8:388. https://doi.org/10.1038/s41746-025-01800-1.
26. Sittig DF, Singh H. A new sociotechnical model for studying health information technology in complex adaptive healthcare systems. Qual Saf Health Care. 2010;19 Suppl 3:i68–74. https://doi.org/10.1136/qshc.2010.042085.
27. Bowen J, Hinze A. Using ontologies to reason about the usability of interactive medical devices in multiple situations of use. EICS’12 - Proceedings of the 2012 ACM SIGCHI Symposium on Engineering Interactive Computing Systems. 2012;:247–56. https://doi.org/10.1145/2305484.2305525.
28. Dogmus Z, Papantoniou A, Kilinc M, Yildirim SA, Erdem E, Patoglu V. Rehabilitation robotics ontology on the cloud. In: IEEE International Conference on Rehabilitation Robotics. IEEE; 2013. p. 1–6. https://doi.org/10.1109/ICORR.2013.6650415.
29. Dogmus Z, Erdem E, Patoglu V. RehabRobo-Onto: Design, development and maintenance of a rehabilitation robotics ontology on the cloud. Robot Comput Integr Manuf. 2015;33:100–9. https://doi.org/10.1016/j.rcim.2014.08.010.
30. Rosier A, Mabo P, Chauvin M, Burgun A. An Ontology-Based Annotation of Cardiac Implantable Electronic Devices to Detect Therapy Changes in a National Registry. IEEE J Biomed Health Inform. 2015;19:971–8. https://doi.org/10.1109/JBHI.2014.2338741.
31. Salvador-Mazenq M. The French pacemaker register. Arch Mal Coeur Vaiss. 2004;97:42–5.
32. Schütz AE, Fertig T, Weber K. Modelling and Development of Intelligent Systems. Cham: Springer International Publishing; 2021. https://doi.org/10.1007/978-3-030-68527-0.
33. Napoli-Spatafora MA. HAO: Hearing Aid Ontology. In: International Conference on Biomedical Ontologies 2021. 2021.
34. de Lauretis L, Costantini S, Pallotta E, Balsano C. An Ontology of Medical Wearables. In: 2022 IEEE International Conference on Consumer Electronics (ICCE). IEEE; 2022. p. 1–4. https://doi.org/10.1109/ICCE53296.2022.9730210.
35. OBO Technical WG. Human Disease Ontology. 2024. https://obofoundry.org/ontology/doid.html. Accessed 23 Sep 2024.
36. Steenwinckel B, De Brouwer M, Stojchevska M, Van Der Donckt J, Nelis J, Ruyssinck J, et al. Data Analytics for Health and Connected Care: Ontology, Knowledge Graph and Applications. 2023. p. 344–60. https://doi.org/10.1007/978-3-031-34586-9_23.
37. Moreira J, Pires LF, van Sinderen M, Daniele L. SAREF4health: IoT Standard-Based Ontology-Driven Healthcare Systems. In: Formal Ontology in Information Systems. 2018. https://doi.org/10.3233/978-1-61499-910-2-239.
38. Esnaola-Gonzalez J, Bermúdez I, Fernández I, Arnaiz A. Two Ontology Design Patterns toward Energy Efficiency in Buildings. Proceedings of the 9th Workshop on Ontology Design and Patterns (WOP 2018) co-located with 17th International Semantic Web Conference (ISWC 2018). 2018.
39. W3C. Time Ontology in OWL. 2022. https://www.w3.org/TR/owl-time/. Accessed 23 Sep 2024.
40. Bughio KS, Cook DM, Shah SAA. Novel Knowledge Graph-Based Modeling for Vulnerability Detection in the Internet of Medical Things. In: Recent Challenges in Intelligent Information and Database Systems. Springer; 2024. p. 314–25. https://doi.org/10.1007/978-981-97-5937-8_26.
