# Supplementary figures and images for: An Ontology for the Adoption of Medical Devices in Health Care Organizations: Design and Development Study
Source: J Med Internet Res. 2026 Jul 10;28:e88366. doi: 10.2196/88366 (PMC13353408; doi:10.2196/88366)

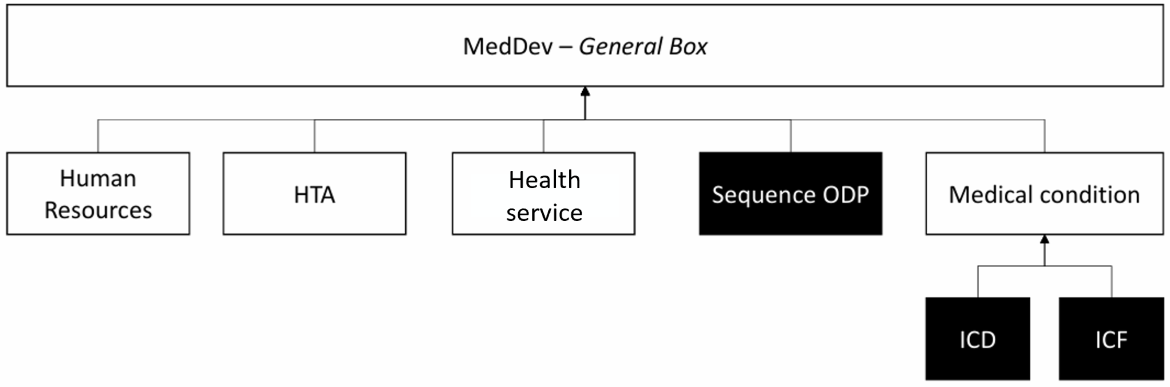

Supplement: Multimedia Appendix 3 [file jmir-v28-e88366-s003.png]
