# Supplementary material for: An Ontology for the Adoption of Medical Devices in Health Care Organizations: Design and Development Study
Source: J Med Internet Res. 2026 Jul 10;28:e88366. doi: 10.2196/88366 (PMC13353408; doi:10.2196/88366)
Supplement: Multimedia Appendix 4 [file jmir-v28-e88366-s004.pdf]

eunethta

EUROPEAN NETWORK FOR HEALTH TECHNOLOGY ASSESSMENT

EUnetHTA Joint Action 3 WP4

Rapid assessment of other technologies using the HTA Core Model®
for Rapid Relative Effectiveness Assessment

LITHIUM TRIBORATE (LBO) LASER FOR PHOTOSELECTIVE VAPORISATION OF THE PROSTATE (PVP) IN THE TREATMENT OF BENIGN PROSTATIC HYPERPLASIA (BPH)

Project ID: OTCA17

Version 1.5, 21st November 2019

This report is part of the project / joint action '724130 / EUnetHTA JA3' which has received funding from the European Union's Health Programme (2014-2020)

DOCUMENT HISTORY AND CONTRIBUTORS

Version	Date	Description
**V1.0**	**22/02/2019**	Early draft prepared by the authors.
**V1.1**	**19/07/2019**	Input from co-author has been processed.
**V1.2**	**10/10/2019**	Input from dedicated reviewers has been processed.
**V1.3**	**08/11/2019**	Input from external experts and manufacturer(s) has been processed.
**V1.4**	**20/11/2019**	Input from medical editor has been processed.
**V1.5**	**21/11/2019**	Final version.

Disclaimer

The assessment represents a consolidated view of the EUnetHTA assessment team members and is in no case the official opinion of the participating institutions or individuals.

EUnetHTA Joint Action 3 is supported by a grant from the European Commission. The sole responsibility for the content of this document lies with the authors and neither the European Commission nor EUnetHTA are responsible for any use that may be made of the information contained therein.

Assessment team

Author(s)	Agenzia Nazionale per i Servizi Sanitari Regionali (AGENAS)
Co-Author(s)	Regione Emilia Romagna (RER)
Dedicated Reviewer(s)	Agencia de Evaluación de Tecnologías Sanitarias de Andalucía (AETSA) and Swiss Network for Health Technology Assessment (SNHTA)

Further contributors

The authors would like to thank Patrizia Brigoni (AGENAS, Italy) for assisting in designing and performing the systematic literature searches and Judit Erdos from Ludwig Boltzmann Institute for Health Technology Assessment (LBI-HTA, Austria) for her patience and kind support on aspects of project management.

Consultation of the draft Rapid Assessment

External experts	Paolo Beltrami<br>Unità Operativa Semplice Dipartimentale di Endourologia Clinica Urologica Dipartimento di Scienze Chirurgiche, Oncologiche e Gastroenterologiche Azienda Ospedaliera - Università di Padova (Italy)<br>Franco Bergamaschi<br>Director of the Urology Unit, Santa Maria Hospital IRCCS, Reggio Emilia (Italy)<br>Michele Colicchia<br>Unità Operativa Semplice Dipartimentale di Endourologia Clinica Urologica Dipartimento di Scienze Chirurgiche, Oncologiche e Gastroenterologiche Azienda Ospedaliera - Università di Padova (Italy)<br>Rafael Medina<br>Director of the Urologist and Nephrologist Clinical Unit, Hospital Virgen del Roció, Sevilla (Spain)
Manufacturer(s)<br>(factual accuracy check)	Anna Creatore<br>Analyst II, Health Economics & Market Access, Boston Scientific (Italy)<br>Stephan Heumann<br>Director, Market Access Urology & Pelvic Health EMEA, Health Economics & Market Access EMEA, Boston Scientific (Germany)
Medical editor V1.4	Compuscript, Ireland

Conflict of interest

All authors, co-authors, dedicated reviewers and external experts involved in the production of this assessment have declared that they have no conflicts of interest in relation to the technology and comparator assessed according to the EUnetHTA Declaration of Interest and Confidentiality Undertaking of Interest (DOICU) statement form.

How to cite this assessment

Please cite this assessment as follows:

EUnetHTA OTCA17 Assessment Team. Lithium triborate (LBO) laser for photoselective vaporisation of the prostate (PVP) in the treatment of benign prostatic hyperplasia (BPH). Collaborative Assessment. Diemen (The Netherlands): EUnetHTA; 2019. Report No.: OTCA17.

This document is available on the website of EUnetHTA (https://www.eunethta.eu/).

**Assessment/Authoring team**		
	Organisation	Individual name
Author(s)	Experts from AGENAS (Italy)	Antonio Migliore<br>Massimiliano Orso<br>Iosief Abraha<br>Emilio Chiarolla<br>Tom Jefferson
Co-Author(s)	Experts from RER (Italy)	Giulio Formoso<br>Luciana Ballini<br>Massimo Vicentini<br>Pamela Mancuso
Dedicated Reviewer(s)	Experts from AETSA (Spain)	Juan Ruano Ruiz
	Experts from SNHTA (Switzerland)	Heike Raatz
Project management	AGENAS (Italy)	Antonio Migliore<br>Nicola Vicari

**Observers**
Anna Maria Vincenza Amicosante (AGENAS) (Italy)
Simona Paone (AGENAS) (Italy)

TABLE OF CONTENTS

DOCUMENT HISTORY AND CONTRIBUTORS 2
TABLE OF CONTENTS 5
LIST OF TABLES AND FIGURES 7
LIST OF ABBREVIATIONS 8
SUMMARY OF RELATIVE EFFECTIVENESS OF LBO LASER FOR PVP IN THE TREATMENT OF BPH 11
SCOPE 11
INTRODUCTION 11
METHODS 11
RESULTS 12
DISCUSSION 30
CONCLUSION 30
1 SCOPE 32
2 METHODS AND EVIDENCE INCLUDED 34
2.1 ASSESSMENT TEAM 34
2.2 SOURCE OF ASSESSMENT ELEMENTS 37
2.3 SEARCH 37
2.4 STUDY SELECTION 38
2.5 DATA EXTRACTION AND ANALYSES 38
2.6 QUALITY RATING 39
2.7 PATIENT INVOLVEMENT 39
2.8 DESCRIPTION OF THE EVIDENCE USED 39
2.9 DEVIATIONS FROM PROJECT PLAN 42
3 DESCRIPTION AND TECHNICAL CHARACTERISTICS OF TECHNOLOGY (TEC) 43
3.1 RESEARCH QUESTIONS 43
3.2 RESULTS 43
4 HEALTH PROBLEM AND CURRENT USE OF THE TECHNOLOGY (CUR) 47
4.1 RESEARCH QUESTIONS 47
4.2 RESULTS 47
5 CLINICAL EFFECTIVENESS (EFF) 52
5.1 RESEARCH QUESTIONS 52
5.2 RESULTS 52
6 SAFETY (SAF) 60
6.1 RESEARCH QUESTIONS 60
6.2 RESULTS 60
7 POTENTIAL ETHICAL, ORGANISATIONAL, PATIENT AND SOCIAL, AND LEGAL ASPECTS (ETH, ORG, SOC, LEG) 66
7.1 RESEARCH QUESTIONS 66
7.2 RESULTS 66
8 DISCUSSION 67
9 CONCLUSION 70
10 REFERENCES 71
APPENDIX 1: METHODS AND DESCRIPTION OF THE EVIDENCE USED 73
DOCUMENTATION OF THE SEARCH STRATEGIES 73
DESCRIPTION OF THE EVIDENCE USED 75

APPENDIX 2: REGULATORY AND REIMBURSEMENT STATUS 86

APPENDIX 3: CHECKLIST FOR POTENTIAL ETHICAL, ORGANISATIONAL, PATIENT AND SOCIAL AND LEGAL ASPECTS 87

APPENDIX 4: MISCELLANEOUS 88

LIST OF TABLES AND FIGURES

Tables

Table 1. *Summary of findings table of LBO laser for PVP in the treatment of BPH: comparison of GL-XPS versus TURP indicating critically important outcomes* 16
Table 2. *Summary of findings table of LBO laser for PVP in the treatment of BPH: comparison of GL-XPS versus HoLEP, indicating critically important outcomes* 26
Table 2.1. *Ratings of importance for each outcome, as rated by all the panellists (n=8)*
Table 2.2. *Description of the outcomes*.. 36
Table 2.3. *Main characteristics of included studies* 40
Table 4.1. *Diagnostic evaluation pathway of patients with bothersome LUTS* 48
Table 4.2. *Recommendations for the use of LBO laser for PVP*.. 50
Table A1. *Characteristics of included studies*.. 76
Table A2. *Risk of bias – study level (RCTs)* 79
Table A3. *Risk of bias – outcome level (RCTs)*... 79
Table A4. *List of ongoing studies on GreenLight XPS (180 W).* 82
Table A5. *Summary table characterising the applicability of a body of studies*........................... 85
Table A6. *Regulatory status of the GreenLight XPS Laser System (Boston Scientific)*.............. 86
Table A7. *Documentation of queries to study authors in the assessment report* 88

Figures

FIGURE 2.1. *Prisma flow chart of the systematic literature search* 38
Figure 5.1. *Assessment of risk of bias in included studies* 54
Figure 5.2. *Rate of dysuria reported by the goliath and Jovanović et al. studies* 56
Figure 5.3. *Duration of catheterisation reported by the Goliath and Jovanović et al. studies*... 56
Figure 5.4 *Length of hospital stay reported by the Goliath and Jovanović et al. studies* 56
Figure 6.1. *Strictures experienced in the 0–12-month timeframe reported by the Goliath and Jovanović et al. studies* 63

LIST OF ABBREVIATIONS

AE	Assessment element
AETSA	Agencia de Evaluación de Tecnologías Sanitarias de Andalucía
AGENAS	Agenzia Nazionale per i Servizi Sanitari Regionali
AMS	American Medical Systems
AOTMiT	Agencja Oceny Technologii Medycznych i Taryfikacji
AUA	American Urological Association
AUR	Acute urinary retention
BPE	Benign prostate enlargement
BPEP	Bipolar plasma enucleation of the prostate
BPH	Benign prostatic hyperplasia
BPO	Benign prostatic obstruction
BOO	Bladder outlet obstruction
CE	Conformité Européenne
CI	Confidence interval
CUA	Canadian Urological Association
CUR	Health Problem and Current Use of the Technology
DALY	Disability-adjusted life year
DAN-PSS	Danish Prostate Symptom Score
DOICU	Declaration of interest and confidentiality undertaking of interest
EAU	European Association of Urology
EFF	Clinical effectiveness
EQ-5D	EuroQol-5D
EU	European Union
FDA	US Food and Drugs Administration
FVC	Frequency volume charts
GL-XPS	GreenLight XPS
GR	Grade of recommendations
GRADE	Grades of Recommendation, Assessment, Development and Evaluation
HoLEP	Holmium laser enucleation of the prostate
Ho:YAG	Holmium:yttrium-aluminium garnet
HRQoL	Health-related quality of life
HVB	Hauptverband der Österreichischen Sozialversicherungsträger
ICD	International Classification of Diseases
ICIQ-MLUTS	International Consultation on Incontinence Questionnaire
ICIQ-UI SF	International Consultation on Incontinence Questionnaire Short Form
ICTRP	International Clinical Trials Registry Platform
IFU	Instructions for use

IIEF-5	International Index of Erectile Function-5
IIEF-15	International Index of Erectile Function-15
IPSS	International Prostate Symptom Score
IPSSQoL	International Prostate Symptom Score-Quality of Life
IQWiG	Institut für Qualität und Wirtschaftlichkeit im Gesundheitswesen
ISRCTN	International Standard Randomised Controlled Trials Number
KTP	Potassium-titanylphosphate
LBO	Lithium triborate
LE	Level of evidence
LMWH	Low-molecular-weight heparin
LUTS	Lower urinary tract symptoms
MD	Mean difference
MeSH	Medical subject headings
MPV	Mean prostate volume
NICE	National Institute for Health and Care Excellence
OABq-SF	Overactive Bladder Questionnaire Short Form
OAC	Oral anticoagulants
OP	Open prostatectomy
PICO	Population, Intervention, Comparison, Outcome
PkEP	Plasmakinetic enucleation of the prostate
PSA	Prostate-specific antigen
PVP	Photoselective vaporisation of the prostate
PVR volume	Postvoid residual urine volume
Qmax	Maximum urine flow rate
QoL	Quality of life
RCT	Randomised controlled trial
REA	Relative Effectiveness Assessment
RER	Regione Emilia-Romagna
RoB	Risk of bias
RR	Risk ratio
SAF	Safety
SF-36	Short Form (36) Health Survey
SNHTA	Swiss Network for Health Technology Assessment
SoF	Summary of findings
TEC	Description and technical characteristics of technology
ThuLEP	Thulium laser enucleation of the prostate
ThuVAP	Thulium laser vaporisation of the prostate
ThuVaRP	Thulium vaporesection of the prostate
ThuVEP	Thulium vapoenucleation of the prostate

Tm:YAG	Thulium:yttrium-aluminium-garnet laser
TRUS	Transrectal ultrasound
TUIP	Transurethral incision of the prostate
TURP	Transurethral resection of the prostate
UK	United Kingdom
USA	United States of America
UTI	Urinary tract infection
VAS	Visual analogue scale
WHO	World Health Organization
WP4	Work Package 4
ZIN	Zorginstituut Nederland

SUMMARY OF RELATIVE EFFECTIVENESS OF LBO LASER FOR PVP IN THE TREATMENT OF BPH

Scope

The scope can be found here: Scope.

Introduction

Description of technology and comparators

Lithium triborate (LBO) laser photoselective vaporisation of the prostate (PVP) uses a high-power laser source to ablate and coagulate obstructive prostatic tissue. GreenLight XPS (GL-XPS), manufactured by Boston Scientific, is the LBO laser system for PVP that has been assessed in the present document and represents the latest version of the technology. The procedure involves passing the laser fibre through a cystoscope to reach the enlarged area of the prostate. The laser energy is then released and vaporisation is performed from the prostatic urethra towards the prostatic capsule. In the management of benign prostatic hyperplasia (BPH), according to the prostate volume and the risk of bleeding sequelae, the following procedures and technologies can be identified as comparators of GL-XPS: transurethral incision of the prostate (TUIP); transurethral resection of the prostate (TURP); open prostatectomy (OP); holmium laser enucleation of the prostate (HoLEP); bipolar enucleation; thulium laser vaporisation of the prostate (ThuVAP); diode laser vaporisation; and laser enucleation **(B0001)**.

GL-XPS was first Conformité Européenne (CE) marked in 2010, and has been US Food and Drug Administration (FDA) approved since 2009 for the surgical incision and/or excision, vaporisation, ablation, haemostasis and coagulation of soft tissue, and has only been used for urological applications **(A0020)**.

GL-XPS claims the following benefits: shorter hospital length of stay (because the procedure can be done as a day-case procedure); shorter duration of catheterisation; quicker return to normal activity following treatment; reduction in pain leading to improved quality of life; can be used for patients taking anticoagulants and those with larger prostates; reduction in hospital readmissions; and reduced risk from capsular perforation, bleeding and TURP syndrome **(B0002)**.

Health problem

BPH is a condition that has adverse impacts on the lower urinary tract function because of the hyperplasia and enlargement of the central transitional zone of the prostate. This can be caused by increased rate of cell proliferation, reduced rate of apoptosis (cell death), or both **(A0002)**. BPH is a progressive disease that, if left untreated, can lead to increased prostate volume, reduction in maximum urinary flow rate and increased lower urinary tract symptoms (LUTS), resulting eventually in acute urinary retention (AUR), which is a medical emergency **(A0004)**. For the present assessment, the target population was defined as specific groups of the general population of patients presenting with bothersome LUTS because of BPH and/or benign prostate enlargement (BPE) having: absolute indication for surgery and being nonresponders to medical treatment or unwilling to undergo medical treatment but requesting active treatment. These groups were identified according to the latest European guidelines and taking in account the advice received from the external clinical experts involved in the assessment. Target populations were defined as men with: (i) prostate volume <30 ml; (ii) prostate volume between 30 and 80 ml; (iii) prostate volume >80 ml; or (iv) at risk of bleeding sequelae who are unable to stop anticoagulation therapy. Recommended treatment options differ among these four groups, and the different comparators are detailed in the Scope. Nevertheless, the technology under assessment (GL-XPS) has been acknowledged as a potential treatment option for each of these four groups **(A0007)**.

Methods

During the preparation of the EUnetHTA project plan, authors, co-authors, dedicated reviewers and external clinical experts agreed to adopt the Grades of Recommendation, Assessment, Development and Evaluation (GRADE) approach to rate the importance of each outcome.

According to their importance, outcomes were rated as ‘critical’, ‘important’ or ‘not important’. Summary of Findings (SoF) tables were completed only for outcomes rated as ‘critical’.

A structured manufacturer questionnaire was used to inform the scoping phase and gather information on the specific technology and its use. Further sources (e.g., manufacturer’s website, technical reports, instructions for use documents, and regulatory body databases) were also used to validate or supplement data in the Description and Technical Characteristics of Technology (TEC) domain.

The latest published clinical guidelines on the management of the condition of interest and *ad hoc* literature searches for epidemiological studies were used to develop the Health Problem and Current Use of the Technology (CUR) domain. Current use of the technology across EUnetHTA partners was described by using data collected through a survey among Work Package 4 (WP4) members. When necessary, information and data presented were integrated with those provided by the manufacturer. Input from clinical experts was considered in particular for the description of the condition, current treatment options and outcome selection.

Clinical Effectiveness (EFF) and Safety (SAF) domains were based on a systematic review of evidence. Only articles published from 1st January 2009 to 13th November 2018 were considered. Details on search strategy are presented in Appendix 1. Searches were run on Embase, Medline and The Cochrane Library. Manual searches of the reference lists of relevant studies were also performed. In addition, the following clinical trials databases were searched to identify relevant studies: clinicaltrials.gov, United Kingdom (UK) Clinical Trials Gateway, International Standard Randomised Controlled Trials Number (ISRCTN) Registry, European Union (EU) Clinical Trials Register and International Clinical Trials Registry Platform (ICTRP). Literature screening was performed independently by two authors. Divergences were solved through discussion with a third author. Relevant data from the selected study were extracted by one author and reviewed by another author. Where possible, data from single studies were pooled with the RevMan 5.3 software using the random-effects model. The authors of the studies were contacted to get data missing from the published articles.

For the TEC and CUR domains, no quality assessment tool was used, but multiple sources were consulted for data validation. For the EFF and SAF domains, two authors independently assessed the quality of evidence of the included studies. The risk of bias (RoB) assessment of the included studies was performed by one author and checked by another author using the Cochrane Risk of Bias (RoB) Tool in the RevMan 5.3 software. RoB tables at study level and outcome level were prepared (Appendix 1). The quality of evidence was assessed using GRADE.

The whole process, from the application of the methods used to the analyses performed, was checked by the co-authors.

Results

Available evidence

After the literature-screening process, three randomised controlled trials (RCTs) enrolling 434 participants in total were included in the EFF and SAF analyses: the GOLIATH non-inferiority study [1–3] and Jovanović et al. [4] for the comparison GL-XPS versus TURP, and the non-inferiority trial by Elshal et al. [5] for the comparison GL-XPS versus HoLEP. The GOLIATH Study [1–3] was financed by the manufacturer, American Medical Systems (AMS), which was also involved in designing and conducting the study, as well as in the statistical analysis; the Elshal trial authors acknowledged conflicts of interest with AMS and Lumenis.

Evidence was found for most outcomes defined by the scope with exception of mortality and frequency of completion as a day-case, neither of which were reported in any of the three included trials. The length of follow-up was 12 months in the Jovanović et al. [4] and Elshal et al. [5] studies, and 24 months in the GOLIATH Study [1–3]. The main results of the effectiveness and safety critical outcomes considered, together with their GRADE assessment, are described later. Results in terms of non-inferiority were reported for the outcomes for which a non-inferiority design and a non-inferiority margin were anticipated, namely International Prostate Symptom Score (IPSS) and maximum urine flow rate (Qmax) for the GOLIATH Study, and IPSS for the Elshal et al. trial. The results for the remaining outcomes were considered in terms of superiority analysis and we use

terms such as 'no difference was found' or 'the results were similar between the groups' to describe differences between outcomes.

Clinical effectiveness (D0005) (D0006) (D0011)

GL-XPS versus TURP

Reduction of symptoms using the IPSS score was evaluated by the two available trials (GOLIATH [1–3] and Jovanović et al. [4]), but it was not possible to pool the data because of insufficient data provided by one of the studies [4]. Low-quality data suggested that, at 6-, 12- or 24-month follow-up, GL-XPS is non-inferior to TURP in terms of reduction of symptoms based on the IPSS score.

Although improvements in Qmax and reduction of postvoid residual urine (PVR) volume were evaluated by both trials, pooling was again not possible because of insufficient data provided by one of the studies [4]. Low-quality data at 6 and 12 months, and moderate-quality data at 24 months showed non-inferiority of GL-XPS versus TURP in improving Qmax and a similar reduction in PVR volume.

Rate of dysuria was reported by both trials [1–4]. Low-quality data from both trials suggested a similar rate of dysuria between the two groups at 12 months.

Length of hospital stay was evaluated by both trials [1–4]. Very low-quality data from both trials indicated a shorter hospital stay in the GL-XPS group than in TURP group. Duration of catheterisation was also evaluated by both trials [1–4] and was shorter in the GL-XPS group than in the TURP group.

Erectile function assessed using the International Index of Erectile Function-5 (IIEF-5) score was evaluated only by the GOLIATH Study, with a follow-up of 12 and 24 months. In addition, overactive bladder symptoms and health-related quality of life (HRQoL) were assessed only by the study [1–3] that used the Overactive Bladder Questionnaire–short form (OABq-SF) Symptom, OABq-SF Health, and the International Consultation on Incontinence Questionnaire-Urinary Incontinence short form (ICIQ-UI SF) with a follow-up of 24 months. No differences were found between GL-XPS and TURP. However, the quality of evidence for all these outcomes was low.

GL-XPS versus HoLEP

Only one trial [5], with a small sample size, compared GL-XPS to HoLEP, showing non-inferiority of GL-XPS versus HoLEP in the reduction of symptoms using the IPSS score, higher Qmax improvement at 4- and 12-month follow-up, and shorter length of catheterisation in the HoLEP group, whereas PVR volume reduction, rates of dysuria and length of hospital stay were similar between the two groups. However, the quality of evidence for all these outcomes was very low.

Erectile function was assessed by the single included study [5] using the International Index of Erectile Function-15 (IIEF-15) score. However, very low-quality data showed no difference between GL-XPS and HoLEP.

Health-related quality of life (D0012) (D0013) (D0016)

GL-XPS versus TURP

The improvement in HRQoL using the International Prostate Symptom Score-Quality of Life (IPSS-QoL) score was evaluated by the only included study with a follow-up of 6, 12 or 24 months [1–3]. Low-quality data showed no differences between the two strategies.

GL-XPS versus HoLEP

The improvement of HRQoL using the IPSS-QoL score was evaluated by the single included study [5] with a follow-up of 12 months. Low-quality data showed no difference between the two strategies.

Patient satisfaction (D0017)

GL-XPS versus TURP

Patient satisfaction was reported by one study [1–3]. At the end of the 24-month follow-up, a similar proportion of patients in the GL-XPS group and in TURP group would undergo the therapy again and would recommend their therapy.

GL-XPS versus HoLEP

No evidence was found to answer this research question.

Safety (C0008) (C0002) (C0004) (C0005) (C0007)

GL-XPS versus TURP

Re-intervention rate was assessed by one study [1–3]. At 30-day follow-up, low-quality data showed that the patients in the GL-XPS group had a significantly lower rate of re-intervention compared with patients in the TURP group, but this difference was not maintained at 6- and 12-month follow-up. The rate of surgical retreatments for obstructions was assessed by one study [1–3]. Low-quality data did not show any difference between the two strategies at 24-month follow-up.

Urinary incontinence was assessed by both available trials [1–4], but it was not possible to pool the data because of insufficient data provided by one of the studies [4]. Low-quality data did not show any difference between the two strategies at 6-, 12- or 24-month follow-up.

Strictures were assessed by both available trials [1–4]. Pooled, very low-quality, data at 12-month follow-up showed no evidence of a difference between GL-XPS and TURP.

Irritative symptoms, including pain and discomfort, urinary tract infections (UTIs) and urinary retention were assessed only by one study [1–3]. Low-quality data did not show any difference between the two strategies at 6-, 12- or 24-month follow-up.

Erectile dysfunction was also only reported by one study [1–3]. Very low-quality data did do not show any differences between the two strategies at 12- or 24-month follow-up.

Procedural transfusions were reported by both trials [1–4]. Patients in the GL-XPS group had a lower risk of being transfused during the surgical procedure compared with patients in the TURP group.

Procedural and postoperative bleeding were evaluated only by one study [1–3]. At 6-, 12- and 24-month follow-ups, there was no evidence of differences between the GL-XPS and TURP groups.

TURP syndrome and rate of capsular perforation were reported only by one study [4]. No evidence of differences was found between the GL-XPS and TURP groups.

The GOLIATH study [1–3] reported that all surgeons were licensed urologists trained and experienced with TURP; however, prior surgical experience with XPS varied widely among surgeons, from <10 cases to >500 cases.

GL-XPS versus HoLEP

For the following safety outcomes, there was very low-quality evidence available from the only study that reported them [5], and suggested no difference between the two strategies at 12-month follow-up: re-intervention rate; urinary incontinence; stricture occurrence; UTI occurrence; and urinary retention.

There was no evidence for differences in procedural transfusions, postoperative haematuria, capsular perforation and conversion rate to monopolar TURP for haemostasis or because of residual prostate tissue in the GL-XPS versus HoLEP groups.

All procedures reported in the included study [5] were performed by a single surgeon experienced in both techniques (they had performed >1200 HoLEP and 400 GL procedures).

Ethical, organisational, patient, and social and legal aspects

Two further research questions related to organisational aspects were answered: effect on length of hospital stay and frequency of completion as a day-case. In the comparison of GL-XPS versus TURP, length of hospital stay was assessed by both the GOLIATH Study [1–3] and Jovanović et al. [4], finding significantly shorter stays in the GL-XPS group than in the TURP group. However, data quality was rated very low. This result should be considered when performing economic analyses because its impact on costs can be relevant. By contrast, in the comparison of GL-XPS versus HoLEP, length of hospital stay did not differ statistically between the two groups.

We did not find any evidence in the included studies to assess the frequency of completion as a day-case. Thus, to clear conclusions on this aspect, specific literature searches and analyses are required (**G0001**).

Upcoming evidence

We identified two studies on GL-XPS labelled as completed or with unknown status. The study labelled as completed (NCT01500057) had a randomised design, enrolled 66 patients to compare GL-XPS versus bipolar vaporisation and was completed in October 2016. No results or publications related to the study were identified. The study with unknown status (NCT02139969) had a retrospective cohort design, aimed to review charts from 1000 patients and complete data analysis by November 2014.

Among the six ongoing studies identified (Table A4, Appendix 1), one was a registry study planning to enrol 300 patients and one was non-comparative. The remaining four studies were comparative. One study (NCT03318991) aimed to enrol 100 patients to compare GL-XPS with thulium laser enucleation of the prostate (ThuLEP) with a follow-up of 12 months. Completion was estimated for June 2019. One study (NCT03305861) aimed to enrol 150 patients with prostate volume ≥80 ml to compare GL-XPS and ThuLEP to HoLEP with a follow-up at 6 months. Completion was estimated for December 2018, but the study record had not been updated. Another study (NCT03297281) aimed to enrol 386 patients taking oral anticoagulant (OAC) therapy with prostate volume ≤80 ml to assess the discontinuation of OAC therapy concomitant with the GL-XPS procedure, with follow-up at 6 months. Completion is expected by May 2020. The final study (NCT02332538) aimed to enrol 182 patients with prostate volume ≥80 ml to compare GL-XPS versus HoLEP versus bipolar TURP with follow-up at 24 months. Completion was estimated by December 2018, but the study record had not been updated.

The upcoming evidence can be considered for an update of the present assessment, in particular in relation to the comparison of GL-XPS versus HoLEP or ThuLEP, and the assessment of the procedure in a population of men treated with OAC. The three ongoing randomised studies could increase the quality of evidence and change the estimates related to some of the critical outcomes, such as IPSS and Qmax score at 12 and 24 months, failure rate, and retreatment at 2 years.

Reimbursement

Specific reimbursement codes have been issued in Austria and Germany. In the other countries, the procedure is reimbursed under an umbrella code for transurethral prostatectomy, irrespective of the technology used.

Table 1. Summary of findings table of LBO laser for PVP in the treatment of BPH: comparison of GL-XPS versus TURP indicating critically important outcomes

Certainty assessment							No. of patients		Effect		Certainty[b]	Importance
**No. of studies**	**Study design**	**Risk of bias**	**Inconsistency**	**Indirectness**	**Imprecision[a]**	**Other considerations**	**GL-XPS**	**TURP**	**Relative (95% CI)**	**Absolute (95% CI)**		
**EFFECTIVENESS**												
**Reduction of symptoms (IPSS score[c]) at 6 months**												
2[d,e]	Randomised trials	Serious[f,g]	Not serious	Not serious	Serious[h]	None	167<br>GOLIATH: 136<br>Jovanović et al.: 31	164<br>GOLIATH: 133<br>Jovanović et al.: 31	No pooled estimates possible<br>GOLIATH: MD 1.2 higher (0.0 lower to 2.4 higher)<br>Jovanović et al.: not reported		⊕⊕◯◯ LOW	CRITICAL
**Reduction of symptoms (IPSS score[c]) at 12 months**												
2[d,e]	Randomised trials	Serious[f,g]	Not serious	Not serious	Serious[h,i]	None	162<br>GOLIATH: 131<br>Jovanović et al.: 31	158<br>GOLIATH: 127<br>Jovanović et al.: 31	No pooled estimates possible<br>GOLIATH: MD 1.2 higher (0.2 lower to 2.6 higher)<br>Jovanović et al.: MD 0.4 higher (CI not reported)		⊕⊕◯◯ LOW	CRITICAL
**Reduction of symptoms (IPSS score[c]) at 24 months**												
1[d]	Randomised trial	Serious[f]	Not serious	Not serious	Serious[h]	None	128	121	—	MD 1.0 higher (0.5 lower to 2.5 higher)	⊕⊕◯◯ LOW	CRITICAL

Certainty assessment							No. of patients		Effect		Certainty[b]	Importance
No. of studies	Study design	Risk of bias	Inconsistency	Indirectness	Imprecision[a]	Other considerations	GL-XPS	TURP	Relative (95% CI)	Absolute (95% CI)		
**Improvement in Qmax[j] (ml/s) at 6 months**												
2[d,e]	Randomised trial	Serious[g]	Not serious	Not serious	Serious[h]	None	165<br>GOLIATH: 134<br>Jovanović et al.: 31	162<br>GOLIATH: 131<br>Jovanović et al.: 31	No pooled estimates possible<br>GOLIATH: MD 1.0 lower (3.6 lower to 1.6 higher)<br>Jovanović et al.: not reported		⊕⊕◯◯ LOW	CRITICAL
**Improvement in Qmax[j] (ml/s) at 12 months**												
2[d,e]	Randomised trials	Serious[g]	Not serious	Not serious	Serious[h,i]	None	162<br>GOLIATH: 131<br>Jovanović et al.: 31	158<br>GOLIATH: 127<br>Jovanović et al.: 31	No pooled estimates possible<br>GOLIATH: MD 1.8 lower (4.3 lower to 0.7 higher);<br>Jovanović et al.: MD 0.2 higher (CI not reported).		⊕⊕◯◯ LOW	CRITICAL
**Improvement in Qmax[j] (ml/s) at 24 months**												
1[d]	Randomised trial	Not serious	Not serious	Not serious	Serious[h]	None	128	121	—	MD 1.3 lower (3.8 lower to 1.2 higher)	⊕⊕⊕◯ MODERATE	CRITICAL
**Improvement in PVR volume (ml) at 6 months**												
2[d,e]	Randomised trial	Serious[g]	Not serious	Not serious	Serious[h]	None	163<br>GOLIATH: 132<br>Jovanović et al.: 31	160<br>GOLIATH: 129<br>Jovanović et al.: 31	No pooled estimates possible<br>GOLIATH: MD 3.8 higher (8.4 lower to 16.0 higher)<br>Jovanović et al.: not reported		⊕⊕◯◯ LOW	CRITICAL

Certainty assessment							No. of patients		Effect		Certainty[b]	Importance
No. of studies	Study design	Risk of bias	Inconsistency	Indirectness	Imprecision[a]	Other considerations	GL-XPS	TURP	Relative (95% CI)	Absolute (95% CI)		
**Improvement in PVR volume (ml) at 12 months**												
2[d,e]	Randomised trials	Serious[g]	Not serious	Not serious	Serious[h,i]	None	160<br>GOLIATH: 129<br>Jovanović et al.: 31	156<br>GOLIATH: 125<br>Jovanović et al.: 31	No pooled estimates possible<br>GOLIATH: MD 9.4 higher (3.1 lower to 21.9 higher)<br>Jovanović et al.: not reported ('…PVR volumes were comparable in both groups…').		⊕⊕◯◯ LOW	CRITICAL
**Improvement in PVR volume (ml) at 24 months**												
1[d]	Randomised trial	Not serious	Not serious	Not serious	Serious[h]	None	128	119	—	MD 10.7 higher (3.5 lower to 24.9 higher)	⊕⊕⊕◯ MODERATE	CRITICAL
**Dysuria 0–12 months**												
2[d,e]	Randomised trials	Serious[g]	Not serious	Not serious	Very serious[k]	None	16/167 (9.6%)	20/164 (12.2%)	RR 0.81 (0.45 to 1.45)	23 fewer per 1000 (from 67 fewer to 55 more)	⊕◯◯◯ VERY LOW	CRITICAL
**Patient-reported outcomes: erectile function (IIEF-5) at 12 months**												
1[d]	Randomised trial	Serious[f]	Not serious	Not serious	Serious[h]	None	129	121	—	MD 1.3 lower (3.3 lower to 0.7 higher)	⊕⊕◯◯ LOW	CRITICAL

Certainty assessment							No. of patients		Effect		Certainty[b]	Importance
No. of studies	Study design	Risk of bias	Inconsistency	Indirectness	Imprecision[a]	Other considerations	GL-XPS	TURP	Relative (95% CI)	Absolute (95% CI)		
**Patient-reported outcomes: erectile function (IIEF-5) at 24 months**												
1[d]	Randomised trial	Serious[f]	Not serious	Not serious	Serious[h]	None	124	119	—	MD 1.0 lower (3.0 lower to 1.0 higher)	⊕⊕◯◯ LOW	CRITICAL
**Patient-reported outcomes: OABq-SF Symptoms at 6 months**												
1[d]	Randomised trial	Serious[f]	Not serious	Not serious	Serious[h]	None	132	129	—	MD 5.1 higher (1.5 higher to 8.7 higher)	⊕⊕◯◯ LOW	CRITICAL
**Patient-reported outcomes: OABq-SF Symptoms at 12 months**												
1[d]	Randomised trial	Serious[f]	Not serious	Not serious	Serious[h]	None	131	125	—	MD 4.0 higher (0.0 higher to 8.0 higher)	⊕⊕◯◯ LOW	CRITICAL
**Patient-reported outcomes: OABq-SF Symptoms at 24 months**												
1[d]	Randomised trial	Serious[f]	Not serious	Not serious	Serious[h]	None	126	120	—	MD 3.4 higher (0.4 lower to 7.2 higher)	⊕⊕◯◯ LOW	CRITICAL
**Patient-reported outcomes: OABq-SF Health at 6 months**												
1[d]	Randomised trial	Serious[f]	Not serious	Not serious	Serious[h]	None	133	129	—	MD 3.6 lower (7.2 lower to 0.0 lower)	⊕⊕◯◯ LOW	CRITICAL

Certainty assessment							No. of patients		Effect		Certainty[b]	Importance
No. of studies	Study design	Risk of bias	Inconsistency	Indirectness	Imprecision[a]	Other considerations	GL-XPS	TURP	Relative (95% CI)	Absolute (95% CI)		
**Patient-reported outcomes: OABq-SF Health at 12 months**												
1[d]	Randomised trial	Serious[f]	Not serious	Not serious	Serious[h]	None	131	122	—	MD 4.3 lower (8.1 lower to 0.5 lower)	⊕⊕◯◯ LOW	CRITICAL
**Patient-reported outcomes: OABq-SF Health at 24 months**												
1[d]	Randomised trial	Serious[f]	Not serious	Not serious	Serious[h]	None	127	120	—	MD 2.6 lower (6.3 lower to 1.1 higher)	⊕⊕◯◯ LOW	CRITICAL
**Patient-reported outcomes: ICIQ-UI SF at 6 months**												
1[d]	Randomised trial	Serious[f]	Not serious	Not serious	Serious[h]	None	132	128	—	MD 1.3 higher (0.5 higher to 2.2 higher)	⊕⊕◯◯ LOW	CRITICAL
**Patient-reported outcomes: ICIQ-UI SF at 12 months**												
1[d]	Randomised trial	Serious[f]	Not serious	Not serious	Serious[h]	None	128	122	—	MD 1.2 higher (0.2 higher to 2.2 higher)	⊕⊕◯◯ LOW	CRITICAL
**Patient-reported outcomes: ICIQ-UI SF at 24 months**												
1d	Randomised trial	Serious[f]	Not serious	Not serious	Serious[h]	None	122	118	—	MD 0.8 higher (0.1 lower to 1.7 higher)	⊕⊕◯◯ LOW	CRITICAL

Certainty assessment							No. of patients		Effect		Certainty[b]	Importance
No. of studies	Study design	Risk of bias	Inconsistency	Indirectness	Imprecision[a]	Other considerations	GL-XPS	TURP	Relative (95% CI)	Absolute (95% CI)		
**Reduction of symptoms using IPSS-QoL score at 6 months**												
1[d]	Randomised trial	Serious[f]	Not serious	Not serious	Serious[h]	None	134	130	—	MD 0.3 higher (0.0 lower to 0.6 higher)	⊕⊕◯◯ LOW	CRITICAL
**Reduction of symptoms using IPSS-QoL score at 12 months**												
1[d]	Randomised trial	Serious[f]	Not serious	Not serious	Serious[h]	None	129	126	—	MD 0.2 higher (0.1 lower to 0.5 higher)	⊕⊕◯◯ LOW	CRITICAL
**Reduction of symptoms using IPSS-QoL score at 24 months**												
1[d]	Randomised trial	Serious[f]	Not serious	Not serious	Serious[h]	None	127	120	—	MD 0.1 higher (0.2 lower to 0.4 higher)	⊕⊕◯◯ LOW	CRITICAL
**SAFETY**												
**Rate of re-intervention at 30 days**												
1[d]	Randomised trial	Not serious	Not serious	Not serious	Very serious[l]	None	4/136 (2.9%)	13/133 (9.8%)	RR 0.30 (0.10 to 0.90)	68 fewer per 1000 (from 126 fewer to 10 fewer)	⊕⊕◯◯ LOW	CRITICAL
**Rate of re-intervention at 6 months**												
1[d]	Randomised trial	Not serious	Not serious	Not serious	Very serious[l]	None	13/136 (9.6%)	18/133 (13.5%)	RR 0.71 (0.36 to 1.38)	40 fewer per 1000 (from 116 fewer to 37 more)	⊕⊕◯◯ LOW	CRITICAL

Certainty assessment							No. of patients		Effect		Certainty[b]	Importance
No. of studies	Study design	Risk of bias	Inconsistency	Indirectness	Imprecision[a]	Other considerations	GL-XPS	TURP	Relative (95% CI)	Absolute (95% CI)		
**Rate of re-intervention at 12 months**												
1[d]	Randomised trial	Not serious	Not serious	Not serious	Very serious[l]	None	16/136 (11.8%)	20/133 (15.0%)	RR 0.78 (0.42 to 1.44)	33 fewer per 1000 (from 114 fewer to 49 more)	⊕⊕◯◯ LOW	CRITICAL
**Rate of surgical retreatment for obstruction 0–24 months**												
1[d]	Randomised trial	Not serious	Not serious	Not serious	Very serious[l]	None	14/136 (10.3%)	10/133 (7.5%)	RR 1.37 (0.63 to 2.97)	28 more per 1000 (from 40 fewer to 96 more)	⊕⊕◯◯ LOW	CRITICAL
**Urinary incontinence 0–6 months**												
1[d]	Randomised trial	Not serious	Not serious	Not serious	Very serious[l]	None	15/136 (11.0%)	4/133 (3.0%)	RR 3.67 (1.25 to 10.76)	80 more per 1000 (from 20 more to 140 more)	⊕⊕◯◯ LOW	CRITICAL
**Urinary incontinence 7–12 months**												
1[d]	Randomised trial	Not serious	Not serious	Not serious	Very serious[l]	None	2/136	2/133	RR 0.98 (0.14 to 6.84)	0.3 fewer per 1000 (from 29 fewer to 29 more)	⊕⊕◯◯ LOW	CRITICAL
**Urinary incontinence 13–24 months**												
1[d]	Randomised trial	Not serious	Not serious	Not serious	Very serious[m]	None	0/136 (0.0%)	0/133 (0.0%)	Not estimable	0 more per 1000 (from 14 fewer to 14 more)	⊕⊕◯◯ LOW	CRITICAL

Certainty assessment							No. of patients		Effect		Certainty[b]	Importance
No. of studies	Study design	Risk of bias	Inconsistency	Indirectness	Imprecision[a]	Other considerations	GL-XPS	TURP	Relative (95% CI)	Absolute (95% CI)		
**Irritative symptoms 0–24 months**												
1[d]	Randomised trial	Not serious	Not serious	Not serious	Very serious[l]	None	31/136 (22.8%)	30/133 (22.6%)	RR 1.01 (0.65 to 1.57)	2 more per 1000 (from 98 fewer to 103 more)	⊕⊕◯◯ LOW	CRITICAL
**Strictures 0–12 months**												
2[d,e]	Randomised trials	Serious[g]	Not serious	Not serious	Very serious[n]	None	9/167 (5.4%)	13/164 (7.9%)	RR 0.69 (0.26 to 1.79)	25 fewer per 1000 (from 79 fewer to 283 more)	⊕◯◯◯ VERY LOW	CRITICAL
**Strictures 13–24 months**												
1[d]	Randomised trial	Not serious	Not serious	Not serious	Very serious[l]	None	1/136 (0.7%)	0/133 (0.0%)	RR 2.93 (0.12 to 71.39)	7 more per 1000 (from 13 fewer to 28 more)	⊕⊕◯◯ LOW	CRITICAL
**Urinary tract infection 0–24 months**												
1[d]	Randomised trial	Not serious	Not serious	Not serious	Very serious[l]	None	28/136 (20.6%)	14/133 (10.5%)	RR 1.96 (1.08 to 3.55)	101 more per 1000 (from 15 more to 186 more)	⊕⊕◯◯ LOW	CRITICAL
**Urinary retention 0–24 months**												
1[d]	Randomised trial	Not serious	Not serious	Not serious	Very serious[l]	None	18/136 (13.2%)	14/133 (10.5%)	RR 1.26 (0.65 to 2.42)	27 more per 1000 (from 50 fewer to 104 more)	⊕⊕◯◯ LOW	CRITICAL

Certainty assessment							No. of patients		Effect		Certainty[b]	Importance
No. of studies	Study design	Risk of bias	Inconsistency	Indirectness	Imprecision[a]	Other considerations	GL-XPS	TURP	Relative (95% CI)	Absolute (95% CI)		
**Erectile dysfunction 0–24 months**												
1[d]	Randomised trial	Serious[n]	Not serious	Not serious	Very serious[l]	None	0/136 (0%)	1/133 (0.8%)	RR 0.33 (0.01 to 7.93)	8 fewer per 1000 (from 28 fewer to 13 more)	⊕◯◯◯ VERY LOW	CRITICAL

Abbreviations: BPH=benign prostatic hyperplasia; CI=confidence interval; GL-XPS=GreenLight XP; IIEF-5=International Index of Erectile Function-5; IPSS=International Prostate Symptom Score; ICIQ-UI SF=International Consultation on Incontinence Questionnaire Short Form; IPSS-QoL=International Prostate Symptom Score-Quality of Life; LBO=lithium triborate; MD=mean difference; OABq-SF=Overactive Bladder Questionnaire Short Form; PVR=postvoid residual urine volume; PVP=photoselective vaporisation of the prostate; Qmax=maximum urine flow rate; RoB=risk of bias; RR=risk ratio; TURP=transurethral resection of the prostate.

[a]Assessment of imprecision bias based on Guyatt et al. [6].

[b]GRADE Working Group grades of evidence:

High quality: we are very confident that the true effect lies close to that of the estimate of the effect.

Moderate quality: we are moderately confident in the effect estimate: the true effect is likely to be close to the estimate of the effect, but there is a possibility that it is substantially different.

Low quality: our confidence in the effect estimate is limited: the true effect might be substantially different from the estimate of the effect.

Very low quality: we have little confidence in the effect estimate: the true effect is likely to be substantially different from the estimate of effect.

[c]The GOLIATH Study [1–3] hypothesized non-inferiority of GL-XPS compared with TURP using a non-inferiority margin of three points for the outcome IPSS; the overall scores of IPSS range from 0 (asymptomatic) to 35 (severely symptomatic). According to this, non-inferiority was demonstrated for each follow-up period.

[d]GOLIATH Study [1–3].

[e]Jovanović et al. [4].

[f]GOLIATH Study [1–3]: serious concern regarding RoB: this was an open trial (performance bias possible for subjective outcomes); detection bias (nonblinded assessment of outcomes) suspected for subjective outcomes.

[g]Jovanović et al. [4] trial: serious concern about RoB: selection bias suspected (methods used to generate the random sequence and to conceal allocation not reported); open trial (performance bias possible for subjective outcomes); detection bias (nonblinded assessment of outcomes) suspected for subjective outcomes; selective reporting because it did not adequately report the results at given follow-up times.

[h]GOLIATH Study [1–3]: small sample size.

[i]Jovanović et al. [4] trial: very small sample size; CI not reported.

[j]GOLIATH Study [1–3] hypothesized non-inferiority of GL-XPS compared with TURP using a non-inferiority margin of –5 ml/s for the outcome Qmax; according to this, non-inferiority was demonstrated for each follow-up period.

[k]CI of pooled estimate is very wide.

[l]Very wide CI.

[m]Wide CI and no events in either group.

[n]GOLIATH Study [1–3]: serious concern regarding RoB: this was an open trial (performance bias possible for subjective outcomes).

Table 2. Summary of findings table of LBO laser for PVP in the treatment of BPH: comparison of GL-XPS versus HoLEP, indicating critically important outcomes

Certainty assessment							No. of patients		Effect		Certainty[b]	Importance
**No. of studies**	**Study design**	**Risk of bias**	**Inconsistency**	**Indirectness**	**Imprecision[a]**	**Other considerations**	**GL-XPS**	**HoLEP**	**Relative (95% CI)**	**Absolute (95% CI)**		
**EFFECTIVENESS**												
**Reduction of symptoms (IPSS score[c]) at 12 months**												
1[d]	Randomised trial	Serious[e]	Not serious	Not serious	Very serious[f]	None	53	50	—	MD 1 higher (0.96 lower to 2.96 higher)	⨁◯◯◯ VERY LOW	CRITICAL
**Improvement in Qmax (ml/s) at 12 months**												
1[d]	Randomised trial	Serious[g]	Not serious	Not serious	Very serious[e]	None	53	50	—	MD 17.1 lower (22.56 lower to 11.64 lower)	⨁◯◯◯ VERY LOW	CRITICAL
**Improvement in PVR volume (ml) at 12 months**												
1[d]	Randomised trial	Serious[f]	Not serious	Not serious	Very serious[e]	None	53	50	—	MD 27 higher (0.72 lower to 54.72 higher)	⨁◯◯◯ VERY LOW	CRITICAL

Certainty assessment							No. of patients		Effect		Certainty[b]	Importance
No. of studies	Study design	Risk of bias	Inconsistency	Indirectness	Imprecision[a]	Other considerations	GL-XPS	HoLEP	Relative (95% CI)	Absolute (95% CI)		
**Dysuria (postoperative)**												
1[d]	Randomised trial	Serious[c]	Not serious	Not serious	Very serious[e]	None	2/53 (3.8%)	0/50 (0.0%)	RR 4.72 (0.23 to 96.01)	38 more per 1000 (from 25 fewer to 100 more)	⊕◯◯◯ VERY LOW	CRITICAL
**Dysuria visual analogue scale at 1 month**												
1[d]	Randomised trial	Serious[c]	Not serious	Not serious	Very serious[e]	None	53	50	—	MD 0.5 lower (1.26 lower to 0.26 higher)	⊕◯◯◯ VERY LOW	CRITICAL
**Reduction of symptoms using the IPSS-QoL score at 12 months**												
1[d]	Randomised trial	Serious[c]**Errore. Il segnalibro non è definito.**	Not serious	Not serious	Very serious[e]	None	53	50	—	MD 0.1 higher (0.4 lower to 0.6 higher)	⊕◯◯◯ VERY LOW	CRITICAL
**SAFETY**												
**Rate of re-intervention 0–12 months**												
1[d]	Randomised trial	Serious[f]	Not serious	Not serious	Very serious[e]	None	3/53 (5.7%)	2/50 (4.0%)	RR 1.42 (0.25 to 8.12)	17 more per 1000 (from 30 fewer to 285 more)	⊕◯◯◯ VERY LOW	CRITICAL

Certainty assessment							No. of patients		Effect		Certainty[b]	Importance
No. of studies	Study design	Risk of bias	Inconsistency	Indirectness	Imprecision[a]	Other considerations	GL-XPS	HoLEP	Relative (95% CI)	Absolute (95% CI)		
**Urinary incontinence at 0–12 months**												
1[d]	Randomised trial	Serious[c]	Not serious	Not serious	Very serious[e]	None	9/53 (17.0%)	12/50 (24.0%)	RR 0.71 (0.33 to 1.53)	70 fewer per 1000 (from 226 fewer to 86 more)	⊕◯◯◯ VERY LOW	CRITICAL
**Strictures**												
1[d]	Randomised trial	Serious[f]	Not serious	Not serious	Very serious[e]	none	0/53 (0.0%)	1/50 (2.0%)	RR 0.31 (0.01 to 7.55)	20 fewer per 1000 (from 73 fewer to 33 more)	⊕◯◯◯ VERY LOW	CRITICAL
**Urinary tract infection 0–12 months**												
1[d]	Randomised trial	Serious[f]	Not serious	Not serious	Very serious[e]	None	1/53 (1.89%)	0/50 (0.0%)	RR 2.83 (0.12, 67.97)	19 fewer per 1000 (from 33 fewer to 70 more)	⊕◯◯◯ VERY LOW	CRITICAL
**Urinary retention 0–12 months**												
1[d]	Randomised trial	Serious[f]	Not serious	Not serious	Very serious[e]	None	6/53 (11.3%)	2/50 (4.0%)	RR 2.83 (0.60 to 13.37)	73 more per 1000 (from 16 fewer to 495 more)	⊕◯◯◯ VERY LOW	CRITICAL

Abbreviations: BPH=benign prostatic hyperplasia; CI=confidence interval; GL-XPS=GreenLight XP; HoLEP=Holmium laser enucleation of the prostate; IPSS=International Prostate Symptom Score; IPSS-QoL=International Prostate Symptom Score-Quality of Life; LBO=lithium triborate; MD=mean difference; PVP=photoselective vaporisation of the prostate; PVR=post-void residual urine volume; Qmax=maximum urine flow rate; RR=risk ratio.

[a]Assessment of imprecision bias based on Guyatt et al. [6].

[b]GRADE Working Group grades of evidence:

High quality: we are very confident that the true effect lies close to that of the estimate of the effect.

Moderate quality: we are moderately confident in the effect estimate: the true effect is likely to be close to the estimate of the effect, but there is a possibility that it is substantially different.

Low quality: our confidence in the effect estimate is limited: the true effect might be substantially different from the estimate of the effect.

Very low quality: we have little confidence in the effect estimate: the true effect is likely to be substantially different from the estimate of effect.

[c]The Elshal trial [5] hypothesized non-inferiority of GL-XPS compared with HoLEP using a non-inferiority margin of three points for the outcome IPSS; the overall score of IPSS ranges from 0 (asymptomatic) to 35 (severely symptomatic). According to this, non-inferiority was demonstrated for IPSS at 12 months.

[d]Elshal et al. [5].

[e]Elshal et al. [5] did not report the method used to conceal allocation clearly and whether the outcome assessor was blinded; this study was considered at risk of performance bias for subjective outcomes; attrition bias is possible because of the exclusion from analyses of 5/55 patients in the HoLEP group; selective reporting was detected for the outcome erectile function (International Index of Erectile Function-5; IIEF-15).

[f]Very small sample size; very wide CI.

[g]Elshal et al. [5] did not report clearly the method used to conceal allocation and whether the outcome assessor was blinded; attrition bias is possible because of the exclusion from analyses of 5/55 patients in the HoLEP group; selective reporting was detected for the outcome erectile function (IIEF-15).

Discussion

We identified comparative studies only reporting on two of the comparisons we were interested in: GL-XPS versus TURP and GL-XPS versus HoLEP. Follow-up was up to 24 months for the comparison GL-XPS versus TURP and 12 months for the comparison GL-XPS versus HoLEP. Although the first can be considered a minimum timeframe for the assessment of some of the selected outcomes, the latter might be too short.

GL-XPS versus TURP

Evidence on two of the critical outcomes (Qmax and PVR improvement at 24 months) was rated as moderate quality according to GRADE. The remaining evidence was rated low or very low. The first serious concern was the RoB. Both available trials were considered at risk of performance bias for outcomes likely to be influenced by behaviour, whereas, for outcomes not likely to be influenced by behaviour, we assumed that the risk of performance bias was unlikely. One of the two studies did not report the methods used to generate the random sequence and to conceal treatment allocation, or whether the outcome assessor (for any critical outcome) was blinded; these circumstances could favour selection bias and detection bias (for subjective outcomes), respectively; in addition, for several outcomes, the trial did not report sufficient data to allow a meta-analysis to be performed. The second reason for a further downgrading was because of imprecision resulting from a small sample size and wide confidence intervals (CIs). The GOLIATH Study had a non-inferiority design but the sample size calculation did not provide sufficient elements to assess its adequacy to demonstrate non-inferiority. In addition, the proportion of patients (dichotomous data) within the non-inferiority margins (3 points for IPSS score, and –5 ml/s for Qmax) was not reported. In Jovanović et al. [4], wide CIs and/or very few events influenced the quality, which was downgraded by at least two levels.

The GOLIATH Study reported that, for primary outcomes (IPSS and Qmax improvement at 6, 12, and 24 months), GL-XPS was non-inferior to TURP (GRADE evidence for IPSS = low; GRADE evidence for Qmax at 24 months = moderate).

Frequency of completion as a day-case, considered a critical outcome, was not reported by any trial, whereas urinary incontinence and urethral and bladder neck strictures were the only critical safety outcomes reported by all three included trials.

Other outcomes, rated as important but not critical, showed some benefits in favour of GL-XPS compared with TURP, that is, the length of catheterisation and the length of hospital stay were both shorter in the GL-XPS group (these outcomes are correlated and, therefore, collinearity might be present).

GL-XPS versus HoLEP

The body of evidence regarding GL-XPS versus HoLEP was limited only to a small, non-inferiority study. The quality of evidence was downgraded by one level because of serious concerns in terms of RoB and by one or more level because of imprecision. In particular, there were important uncertainties around the conclusion of non-inferiority between the two interventions in most of the effectiveness outcomes. Similarly, no differences between the two groups were observed in most of safety outcomes reported, whereas significant differences in favour of HoLEP were observed in terms of bleeding (postoperative haematuria) and rate of conversion to TURP, although the quality of evidence was very low.

Conclusion

Our systematic review did not find evidence of good-quality data supporting most of the claimed benefits of GL-XPS versus its comparators.

For the comparison of GL-XPS versus TURP, the quality of evidence was judged as moderate according to GRADE for the outcomes Qmax and PVR improvement and suggests that, for these two outcomes, GL-XPS is non-inferior to TURP. Given the quality of evidence from low to very low of the remaining effectiveness and safety critical, our confidence in their effect estimates is limited.

For the comparison of GL-XPS versus HoLEP, given the very low quality of available evidence, we have little confidence in the effect estimates.

Therefore, further research is needed because the body of available comparative evidence does not cover the four groups defined within the scope of the present assessment. Follow-up exceeding 24 months should be considered for proper assessment of some of the outcomes (especially re-intervention rate).

1 SCOPE

Description	Project scope
**Population**	Male diagnosed with voiding obstruction because of BPH causing moderate-to-severe LUTS in whom surgical intervention is indicated (i.e., with absolute indications for surgery or nonresponders to medical treatment or those who do not want medical treatment but request surgical treatment). In particular:<br>i) Men with prostate volume <30 ml;<br>ii) Men with prostate volume between 30 and 80 ml;<br>iii) Men with prostate volume >80 ml;<br>iv) Men at risk of bleeding sequelae who cannot stop anticoagulation therapy.<br><br>International Classification of Disease (ICD)-10-CM Diagnosis Code: N40.1<br>ICD-9-CM Diagnosis Code: 600.01<br><br>Medical Subject Headings (MeSH) terms: Lower Urinary Tract Symptoms [C23.888.942.343], Prostatism [C23.888.942.343.600], Prostatic Hyperplasia [C12.294.565.500], Urinary Bladder Neck Obstruction [C12.777.767.700.962].<br><br>LBO laser PVP is intended for the treatment of the condition.
**Intervention**	PVP using LBO laser<br><br>Product name: GreenLight (GL)-XPS (Boston Scientific)<br><br>MeSH terms: Laser Therapy [E02.594, E04.014.520]
**Comparison**	The following comparators will be considered:<br>i) TUIP in men with prostate volume <30 ml;<br>ii) TURP in men with prostate volume between 30 and 80 ml;<br>iii) OP, HoLEP or bipolar enucleation in men with prostate volume >80 ml;<br>iv) ThuVAP, diode laser vaporisation or laser enucleation in men at risk of bleeding sequelae who cannot stop anticoagulation therapy.<br><br>MeSH terms: Transurethral Resection of Prostate [E04.950.774.860.625.750]<br><br>Rationale: Comparisons have been defined according to the latest European Association of Urology (EAU) Guidelines[1] considering those interventions indicated as current standard or first choice for the specific patient groups.
**Outcomes**	**<u>Critical outcomes</u>**<br>**Effectiveness**<br>o Reduction of symptoms using the IPSS score<br>o Improvement of HRQoL using the IPSS-QoL score<br>o Improvement in Qmax<br>o Improvement in PVR volume<br>o Rate of dysuria (pain)<br>o Patient-reported outcomes (sexual function, nondisease-specific quality of life)

[1] Gravas S, Cornu JN, Drake MJ, et al. [Internet]. 2018 EAU Guidelines on Management of Non-Neurogenic Male Lower Urinary Tract Symptoms (LUTS), incl. Benign Prostatic Obstruction (BPO) [cited 2019 Mar 8]. Available from https://uroweb.org/wp-content/uploads/EAU-Guidelines-on-the-Management-of-Non-neurogenic-Male-LUTS-2018-large-text.pdf.

	**Safety**<br>o Rate of re-intervention (at any time)<br>o Established urinary incontinence<br>o Irritative symptoms<br>o Any procedure or device-related adverse events (e.g., incontinence, erectile dysfunction, urethral and bladder neck strictures)<br><br>**<u>Important outcomes</u>**<br>**Effectiveness**<br>o Duration of catheterisation<br>o Length of hospital stay<br>o Frequency of completion as a day-case<br>**Safety**<br>o Mortality<br>o Procedural blood loss and blood transfusion need<br>o Rate of TURP syndrome<br>o Rate of capsular perforation<br><br>Rationale: Outcomes were identified according to clinical guidelines[2] and EUnetHTA guidelines[3, 4, 5]. The rating of the relative importance of outcomes was performed at the start of the assessment phase by authors, co-authors, dedicated reviewers and clinical experts according to the GRADE approach. Each outcome was rated as 'critical', 'important but not critical' or 'of limited importance'.
**Study design**	RCTs and comparative prospective nonrandomised studies.

[2] Gravas S, Cornu JN, Drake MJ, et al. [Internet]. 2018 EAU Guidelines on Management of Non-Neurogenic Male Lower Urinary Tract Symptoms (LUTS), incl. Benign Prostatic Obstruction (BPO) [cited 2019 Mar 8]. Available from https://uroweb.org/wp-content/uploads/EAU-Guidelines-on-the-Management-of-Non-neurogenic-Male-LUTS-2018-large-text.pdf.

[3] EUnetHTA. Guideline - Endpoints used for Relative Effectiveness Assessment Clinical Endpoints. 2015.

[4] EUnetHTA. Guideline - Endpoints used in Relative Effectiveness Assessment - SAFETY. 2015.

[5] EUnetHTA. Guideline - Endpoints used for Relative Effectiveness Assessment Health related quality of life and utility measures. 2015.

2 METHODS AND EVIDENCE INCLUDED

2.1 Assessment team

As author, AGENAS:

- Coordinated and managed the whole project, from the scoping phase to the assessment production.
- Coordinated and participated in the GRADE process for the rating of the importance of outcomes.
- Prepared the EUnetHTA project plan.
- Performed the literature search and study selection.
- Carried out the assessment (extraction, analysis, summary and interpretation of findings).
- Shared the first draft assessment with the co-author and implemented changes after discussion.
- Shared the first draft assessment with dedicated reviewers, provided replies to dedicated reviewers' comments, and amended the text accordingly.
- Shared the second draft assessment with external experts, provided replies to experts' comments, and amended the text accordingly.
- Shared the second draft assessment with manufacturers for fact checking, provided replies to manufacturers' comments, and amended the text accordingly.
- Prepared the final assessment and wrote a final summary of the assessment.

As co-author, RER:

- Contributed to the preparation of the EUnetHTA Project Plan.
- Participated in the GRADE process for the rating of the importance of outcomes.
- Checked and approved all steps (e.g., search strategy, literature selection, data extraction, assessment of RoB, and GRADE) and provided methodological support.
- Reviewed the first and second draft assessments, proposed amendments where necessary (performed additional hand searches when needed) and provided written feedback.
- Assisted in the analysis of comments from dedicated reviewers, clinical experts, and manufacturers.
- Contributed to the elaboration of conclusions.

As dedicated reviewers, AETSA and SNHTA:

- Participated in the GRADE process for the rating of the importance of outcomes.
- Guaranteed quality assurance by thoroughly reviewing the project plan and the assessment drafts.
- Reviewed methods, results and conclusions based on the original studies included.
- Provided constructive comments in all project phases.
- The Assessment team, in addition, received the contribution from external experts, who:

- Participated in the GRADE process for the rating of the importance of outcomes.
- Reviewed and discussed the EUnetHTA project plan.
- Reviewed and discussed the second draft assessment.

Definition of the importance of outcomes

During the preparation of the EUnetHTA project plan, authors, co-authors, dedicated reviewers and external clinical experts agreed to adopt the GRADE approach to rate the importance of each outcome. The preliminary list of outcomes defined during the scoping phase was circulated among eight panellists (representing the assessment team and external experts). Panellists were asked to rate the importance of each outcome, according to a 1–9 point scale (‘1’ indicating the lowest importance and ‘9’ indicating the highest importance). After completion of the rating round, the median of the votes was computed and each outcome was assigned a rate of importance: ‘critical’ (median between 7 and 9); ‘important’ (median from 4 to 6) and ‘not important’ (median from 1 to 3). In Table 2.1, ratings of importance are reported for each outcome. Further discussions among the assessment team led to amendments to reduce redundancy and overlaps between outcomes. A final list of outcomes was then defined, following validation by the clinical experts, and is presented within the Scope. It was agreed that Summary of Findings (SoF) tables would be completed only for outcomes rated as ‘critical’, whereas, for outcomes rated as ‘important’, results would be reported and commented on in the main text. A brief description of the outcomes is presented in Table 2.2.

Table 2.1. Ratings of importance for each outcome, as rated by all the panellists (*n* = 8)

	Not important			Important			Critical			Median	Min	Max
**Answer options**	**1**	**2**	**3**	**4**	**5**	**6**	**7**	**8**	**9**			
Reduction of symptoms using IPSS and IPSS-QOL scores	0	0	0	0	0	1	3	0	4	**8**	6	9
Improvement in Qmax and PVR volume	0	0	0	0	2	1	4	1	0	**7**	5	8
Duration of catheterisation	1	0	2	1	0	1	2	0	1	5	1	9
Rate of dysuria (pain)	0	0	1	0	0	2	2	3	0	**7**	3	8
Length of hospital stay	0	0	0	2	2	2	1	0	1	5.5	4	9
Frequency of completion as a day-case	0	0	0	1	2	3	2	0	0	6	4	7
Patient-reported outcomes (sexual function, nondisease-specific quality of life)	0	0	0	0	0	3	1	1	3	**7.5**	6	9
Mortality	0	0	3	2	0	1	0	0	2	4	3	9
Rate of re-intervention (at any time)	0	0	0	1	2	1	0	0	4	**7.5**	4	9
Procedural blood loss and blood transfusion need	0	0	1	2	1	0	0	4	0	6.5	3	8
Bladder outlet obstruction	0	0	0	1	1	2	2	2	0	6.5	4	8
Rate of TURP syndrome	0	0	0	2	2	0	2	2	0	6	4	8
Rate of capsular perforation	0	0	0	1	3	2	0	2	0	5.5	4	8
Established urinary incontinence	0	0	0	1	0	2	2	0	3	**7**	4	9
Irritative and obstructive symptoms	0	0	0	0	2	0	2	1	3	**7.5**	5	9
Any procedure or device-related adverse events (e.g., incontinence, erectile dysfunction, or urethral and bladder neck strictures)	0	0	1	1	0	1	0	2	3	**8**	3	9

Table 2.2. Description of the outcomes

Outcome	Description
Reduction of symptoms using the IPSS score	IPSS score is an eight-question written screening tool used to screen for, rapidly diagnose, track the symptoms of, and suggest management of the symptoms of BPH
Improvement of HRQoL using the IPSS-QoL score	The IPSS-QoL score is the eighth and final question of the IPSS score, relating to the patient's perceived quality of life
Improvement in Qmax	Qmax is the volumetric flow rate of urine during urination
Improvement in PVR volume	PVR volume is the amount of urine remaining in the bladder at the completion of micturition
Rate of dysuria (pain)	Rate of dysuria (pain): dysuria refers to painful urination
Patient reported outcomes	Sexual function, nondisease-specific quality of life
Rate of re-intervention (at any time)	Re-intervention refers to subsequent intervention with the same patients to resolve the same symptoms
Established urinary incontinence	Any uncontrolled leakage of urine that persists after the procedure
Irritative symptoms	Groups of symptoms related to LUTS, including urgency (feeling an urgent need to urinate), frequency (a short time between needing to urinate), nocturia (waking from sleep to pass urine two or more times during the night), urge incontinence (a sudden, intense urge to urinate followed by an uncontrolled loss of urine)
Any procedure or device-related adverse events	Incontinence, erectile dysfunction, urethral and bladder neck strictures
Duration of catheterisation	Number of days in which the patient had a urinary catheter in place
Length of hospital stay	Number of days in which the patient was hospitalised
Frequency of completion as a day-case	Number of procedures performed as a same-day surgery
Mortality	Occurrence of deaths among the patients in the population of study
Procedural blood loss and blood transfusion need	Haemorrhage occurring during the procedure and subsequent need for blood transfusion
Rate of TURP syndrome	TURP syndrome is fluid overload and iso-osmolar hyponatraemia during a TURP procedure from large volumes of irrigation fluid being absorbed through venous sinuses
Rate of capsular perforation	Capsular perforation comprises damage to the prostatic capsula because of deep vaporisation

2.2 Source of assessment elements

The selection of assessment elements is based on the HTA Core Model Application for Rapid Relative Effectiveness Assessment (REA) (4.2). Additionally, assessment elements from other HTA Core Model Applications (for medical and surgical interventions, diagnostic technologies or screening) have been screened and included and/or merged with the existing questions if deemed relevant. The selected issues (generic questions) were translated into actual research questions (answerable questions). Please note that some research questions were answered together; these questions are listed below each other, followed by the answer.

2.3 Search

A manufacturer questionnaire was structured by the authors and used to inform the scoping phase and to gather information on the following: the health condition addressed by the technology; standard of care for the condition; technical characteristics of the technology; current use of the technology; regulatory aspects; published/ongoing clinical studies; registries; costs data; and economic evaluations performed. The process followed is described in the Project Plan. The manufacturer's website, technical reports, instructions for use (IFU) documents, and regulatory body databases were also used as a source to answer assessment elements (AEs) belonging to TEC domain.

The AEs belonging to the CUR domain were answered referring to the latest published clinical guidelines on the management of the condition of interest. Ad hoc literature searches were performed to identify the latest and most relevant epidemiological studies. Current use of the technology across EUnetHTA partners was described by using data collected through a survey among WP4 members. When necessary, information and data presented were integrated with those provided by the manufacturer. Input from clinical experts was considered in particular for the description of the condition, current treatment options and outcome selection.

AEs belonging to the domains EFF and SAF were answered through systematic review of the evidence. To identify primary studies fulfilling the inclusion criteria outlined in the Scope of the present assessment, a systematic literature search was performed. Only articles published from 1st January 2009 to 13th November 2018 were considered. This timeframe was set because the technology of interest, GL-XPS, received approval from FDA in 2009 (CE mark was gained in 2010). We also checked whether further studies were published before 2009 and did not find any of relevance to the scope of the present assessment.

Restrictions and detailed tables on search strategy are included in Appendix 1.

The following sources of information were used in the search:

- Embase;
- Medline;
- The Cochrane Library;
- Manual search (of the reference list of relevant studies identified).

In addition, the following clinical trial databases were searched to identify ongoing studies on GL-XPS:

- Clinicaltrials.gov;
- UK Clinical Trials Gateway;
- ISRCTN Registry;
- EU Clinical Trials Register;
- ICTRP.

2.4 *Study selection*

The literature-screening process was performed independently by two authors (IA and MO). Divergences were solved through discussion with a third author (AM). The flow chart of the selection process is presented in Figure 2.1.

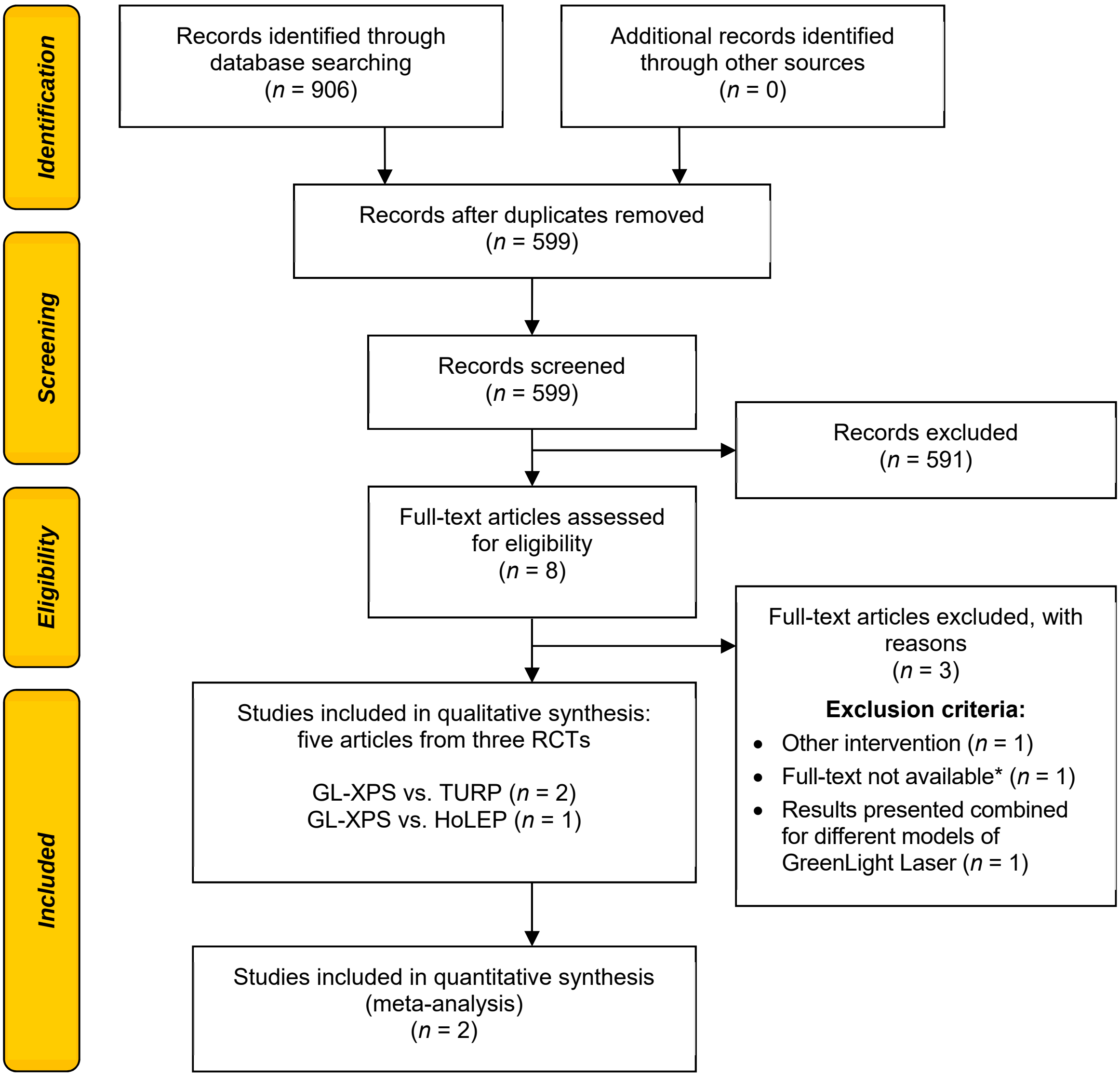

Figure 2.1. PRISMA flow chart of the systematic literature search

Abbreviations: GL-XPS=GreenLight XPS; HoLEP=holmium laser enucleation of the prostate; RCT=randomised controlled trial; TURP=transurethral resection of the prostate.

*The corresponding author of the study was contacted to get the full-text article, but no reply was received (Appendix 4).

2.5 *Data extraction and analyses*

Relevant data from the selected studies were extracted into a predefined data extraction table. The single-data extraction method with verification of another researcher was used: one reviewer (MO) extracted the data and another reviewer (IA) controlled the extracted data. The following characteristics of the included studies were extracted: study ID; objectives; study design; country/setting; patients' characteristics at baseline; effectiveness and safety outcomes; length of

follow-up; and funding. Where possible, data from single studies were pooled using the RevMan 5.3 software. Data were pooled using the random-effects model.

As a result of unreported data for each follow-up period and/or missing standard deviations for reported means in two of the included studies, we contacted the authors to obtain these missing data, but we did not get any reply (Appendix 4). We were able to pool data only in the comparison of GL-XPS versus TURP for the outcomes dysuria and strictures at 12 months, and for duration of catheterisation and length of hospital stay.

2.6 Quality rating

For the TEC and CUR domains, no quality assessment tool was used; instead, multiple sources were used to validate individual, possibly biased, sources. Descriptive analysis of different information sources was performed.

For the EFF and the SAF domains, two authors (IA and MO) independently assessed the quality of evidence of included studies. The RoB assessment of the included studies was conducted by one reviewer (IA) and checked by another reviewer (MO) with the Cochrane Risk of Bias (RoB) Tool using the RevMan 5.3 software. RoB tables at study level and outcome level are presented in Appendix 1. The following domains for the RoB at study level were considered: (i) random sequence generation (selection bias); (ii) allocation concealment (selection bias); (iii) blinding of patients and personnel (performance bias); (iv) blinding of outcome assessment (detection bias); (v) incomplete outcome data (attrition bias); and (vi) selective reporting (reporting bias). At the outcome level, we considered: (i) blinding of outcome assessment (detection bias); (ii) incomplete outcome data (attrition bias); (iii) selective outcome reporting (reporting bias); (iv) other source of bias; and (v) overall RoB at outcome level.

In addition, we assessed the quality of the body of evidence using GRADE (Appendix 1). The process was checked by the co-authors.

2.7 Patient involvement

During the scoping phase of the project, both the authoring team and the WP4 Co-Lead Partner attempted to identify relevant patient associations to involve in the definition of Population, Intervention, Comparison, Outcome (PICO; in particular, outcomes that are supposed to be relevant from the point of view of the patient). Internet searches and enquires to the clinical experts involved in the project did not give any useful result. One potentially relevant association was finally contacted but communication was discontinued after the project presentation and description of tasks were requested. No further attempts at patient involvement were made.

2.8 Description of the evidence used

We included three RCTs in the EFF and SAF analyses: the GOLIATH Study [1–3] and Jovanović et al. [4] for the GL-XPS versus TURP comparison, and Elshal et al. [5] for the GL-XPS versus HoLEP comparison. No further studies fulfilling our inclusion criteria were identified. Results in terms of non-inferiority were reported for the outcomes for which a non-inferiority design and a non-inferiority margin were anticipated, namely IPSS and Qmax for the GOLIATH Study, and IPSS for the Elshal et al. trial. The results for the remaining outcomes were considered in terms of superiority analysis and we used terms such as ‘no difference was found’ or ‘the results were similar between the groups’.

The main characteristics of the included studies are presented in Table 2.3. Detailed information about the studies can be found in Appendix 1, Table A1.

Table 2.3. Main characteristics of included studies

Study ID	Study design (country/setting)	Participants: Intervention group	Participants: Control group	Outcomes	Follow up (months)
		**GL-XPS**	**TURP**		
**GOLIATH – NCT01218672 [1–3]**	Open-label, multicentre, randomised, non-inferiority trial (29 centres in nine European countries)	Patient candidates for the surgical relief of BPO, with IPSS scores of ≥12 and prostate sizes ≤100 g<br>• Number: 136<br>• Age (years): 65.9±6.8<br>• MPV (TRUS, ml): 48.6±19.2<br>• PVR: 110.1±88.5<br>• PSA (ng/ml): 2.7±2.1<br>• IPSS score: 21.2±5.9<br>• Qmax (ml/s): 9.5±3.0<br>• IPSS-QoL: 4.6±1.1<br>• Anticoagulant use: 5 (3.7%)<br>• OABq-SF symptoms: 44.2±20.5<br>• OABq-SF health: 59.0±21.9<br>• ICIQ-UI SF: 3.9±4.7<br>• IIEF-5: 13.2±7.6	• Number: 133<br>• Age (years): 65.4±6.6<br>• MPV (TRUS, ml): 46.2±19.1<br>• PVR: 109.8±103.9<br>• PSA (ng/ml): 2.6±2.1<br>• IPSS score: 21.7±6.4<br>• Qmax (ml/s): 9.9±3.5<br>• IPSS-QoL: 4.5±1.4<br>• Anticoagulant use: 9 (6.8%)<br>• OABq-SF symptoms: 42.9±20.8<br>• OABq-SF health: 62.6±21.7<br>• ICIQ-UI SF: 4.4±4.6<br>• IIEF-5: 13.7±7.5	***Primary eff.:***<br>- IPSS score<br>- Qmax (ml/s)<br>- Complication free<br>- Surgical retreatments<br>***Secondary eff.:***<br>- Prostate volume (TRUS; ml)<br>- PVR (ml)<br>- PSA (ng/ml)<br>- IPSS-QoL<br>QoL: OABq-SF symptoms, OABq-SF health, ICIQ-UI-SF, IIEF-5, EQ-5D, SF-36 Mental Health, SF-36 Physical Health.<br>***Safety:***<br>- Bleeding<br>- Urinary tract infection<br>- Irritative symptoms<br>- Stricture<br>- Urinary incontinence<br>- Urinary retention<br>- Other	6<br>12<br>24
		**GL-XPS**	**TURP**		
**Jovanović 2014 [4]**	RCT (Clinic of Urology, Clinical Center of Serbia, Belgrade)	Patients with moderate or severe LUTS (IPSS >16), failure of previous medical treatment with a washout period of at least 2 weeks, Qmax <15 ml/s, PVR urine >100 ml, prostate volume (TRUS) <100 ml<br>• Number: 31<br>• Age (years)[a]: 66.3 (9.4)<br>• MPV (TRUS, ml): 61.8±22<br>• PVR: 106.2±25<br>• PSA (ng/ml): 2.6±1.8<br>• IPSS score: 27.2±2.3<br>• Qmax (ml/s): 6.9±2.2<br>• Patients preoperatively catheterised: 6 (19%)	• Number: 31<br>• Age (years)[a]: 67.1 (8.0)<br>• MPV (TRUS, ml): 60.3±20<br>• PVR: 114±21<br>• PSA (ng/ml): 2.8±1.4<br>• IPSS score: 27.9±2.7<br>• Qmax (ml/s): 6.4±2.0<br>• Patients preoperatively catheterised: 5 (16%)	***Primary eff.:***<br>- IPSS score<br>- Qmax (ml/s)<br>- PVR<br>***Secondary eff.:***<br>- Operative time<br>- Haemoglobin levels (preoperative/intraoperative)<br>- Duration of catheterisation<br>- Length of hospital stay<br>***Safety:***<br>- Blood transfusion<br>- Capsule perforation<br>- TURP syndrome<br>- Clot retention<br>- Dysuria/urge<br>- Bladder neck contracture<br>- Urethral stricture<br>- Urinary incontinence	1<br>3<br>6<br>12
		**GL-XPS**	**HoLEP**		

**Elshal 2015 [5] – NCT01494337**	Randomised noninferiority trial (Royal Victoria Hospital, Montreal, Quebec, Canada)	Patients >50 years, refractory LUTS secondary to BPH, IPSS >15, QOL score ≥3, Qmax <15 ml/s or patients with acute urinary retention secondary to BPH in whom trial of voiding failed, and prostate size on preoperative TRUS of 40–150 ml.		***Primary eff.:***<br>- IPSS score<br>***Secondary eff.:***<br>- Prostate volume (TRUS; ml)<br>- PVR<br>- PSA<br>- Qmax<br>- IPSS-QOL<br>- IIEF-15<br>- Dysuria<br>- Duration of catheterisation<br>- Length of hospital stay<br>***Safety:***<br>Perioperative and postoperative complications:<br>- Postoperative dysuria<br>- Postoperative pyrexia<br>- Operative prostate capsule violation<br>- Operative bladder wall injury<br>- Inability to void (retention)<br>- Postoperative haematuria (grade 2 early/grade 3a late)<br>- Anaemia requiring transfusion<br>- Epididymo-orchitis<br>- Urosepsis<br>- Recurrent urinary tract infection<br>- Postoperative urge urinary incontinence<br>- Postoperative stress urinary incontinence<br>- Residual prostate adenoma<br>- Bladder neck contracture<br>- Urethral stricture<br>- Prostatic urethral stone and encrusts	1<br>4<br>12
		• Number: 50[b]<br>• Age (years): 74.1±8.8<br>• MPV (TRUS, ml): 83.3±27.8<br>• PVR: 172±137<br>• PSA (ng/ml): 5.3±12.6<br>• IPSS score: 23.0±4.8<br>• Qmax (ml/s): 8.0±3.0<br>• IPSS-QoL: 4.9±1.1<br>• Anticoagulant use:<br>- aspirin: 11 (20.7%)<br>- bridging by LMWH: 15 (24.3%)<br>• Indwelling catheter: 23 (43.4%)<br>• IIEF-15: 45.8±17	• Number: 53<br>• Age (years): 71±9.3<br>• MPV (TRUS, ml): 87.1±28.1<br>• PVR: 146±105<br>• PSA (ng/ml): 5.6±4.4<br>• IPSS score: 22.4±5.6<br>• Qmax (ml/s): 7.5±1.3<br>• IPSS-QoL: 3.8±1.2<br>• Anticoagulant use:<br>- aspirin: 6 (12%)<br>- bridging by LMWH: 12 (24%)<br>• Indwelling catheter: 23 (46%)<br>• IIEF-15: 55.6±15.4		

Abbreviations: BPH=benign prostatic hyperplasia; BPO=benign prostatic obstruction; EQ-5D=EuroQol-5D; GL-XPS=GreenLight XPS; HoLEP=holmium laser enucleation of the prostate; ICIQ-UI SF=International Consultation on Incontinence Questionnaire Short Form; IIEF-5=International Index of Erectile Function-5; IIEF-15=International Index of Erectile Function-15; IPSS=International Prostate Symptom Score; IPSS-QoL=International Prostate Symptom Score-Quality of Life; LMWH=low-molecular-weight heparin; LUTS=lower urinary tract symptoms; MPV=mean prostate volume; OABq-SF=Overactive Bladder Questionnaire Short Form; PSA=prostate specific antigen; PVR=post-void residual urine volume; Qmax=maximum urine flow rate; SF-36=Short Form (36) Health Survey; TRUS=transrectal ultrasound; TURP=transurethral resection of the prostate.

[a]Median (interquartile range).

[b]Originally, 55 patients were allocated to the intervention group; one patient did not receive the allocated intervention, and four other patients were excluded from the final analysis for other reasons.

2.9 Deviations from project plan

According to the project plan, we were supposed to use the Cochrane RoB 2.0 to assess the RoB of the included studies. However, we decided to use the previous version of the Cochrane RoB, because it was available in the RevMan software used for the systematic review.

3 DESCRIPTION AND TECHNICAL CHARACTERISTICS OF TECHNOLOGY (TEC)

3.1 *Research questions*

Element ID	Research question
B0001	What is LBO laser PVP? What are TUIP, TURP, OP, bipolar enucleation, HoLEP, ThuVAP, diode laser vaporisation and laser enucleation?
A0020	For which indications has LBO laser received CE marking?
B0002	What is the claimed benefit of LBO laser PVP in relation to its comparators?
B0003	What is the phase of development of LBO laser PVP and its comparators?
B0004	Who administers LBO laser PVP and its comparators and in what context and level of care are they provided?
B0009	What equipment and supplies are needed to perform LBO laser PVP?
A0021	What is the reimbursement status of LBO laser PVP?

3.2 *Results*

Features of the technology and comparators

[B0001] – What is LBO laser PVP? What are TUIP, TURP, OP, bipolar enucleation, HoLEP, ThuVAP, diode laser vaporisation and laser enucleation?

The technology

LBO laser PVP uses a high-power laser source that emits visible green light with a wavelength of 532 nm to ablate and coagulate obstructive prostatic tissue in a haemostatic fashion. Such specific wavelength light is absorbed by oxyhaemoglobin (present in blood and tissue) and vaporises the tissue without leaving fragments behind.

GL-XPS, manufactured by Boston Scientific, is the LBO laser system for PVP that has been assessed in the present document and is the latest version of the technology. The system mainly comprises a power generator and control unit (GreenLight XPS Console; risk class IIB) and a single-use proprietary laser fibre (GreenLight MoXy Optic Fibre; risk class IIA), which is actively cooled using a flow of saline solution. The PVP procedure involves passing the 70° side-firing laser fibre through a cystoscope to reach the enlarged area of the prostate. The laser energy is then released and vaporisation is performed, following a specific pattern of movement, from the prostatic urethra towards the prostatic capsule i.e., inside-out). The system can operate in either vaporisation or coagulation mode [7].

The comparators

According to the latest European guidelines [8], BPO causing LUTS can be managed with different procedures and technologies. Factors, such as risk of bleeding, possibility of having surgery under anaesthesia, suspension of antiplatelet therapy, and prostate volume, have a role in treatment choice.

Transurethral incision or resection of the prostate

TUIP involves incising the bladder outlet without tissue removal. A resectoscope is inserted through the urethra and used to make one or two small grooves in the bladder neck to open the urinary channel. This technique is the treatment of choice especially in patients with a prostate volume <30 ml without a middle lobe [8].

TURP involves removal of enlarged prostatic tissue. A resectoscope is inserted through the urethra and used to perform a localised resection to open the urinary channel. TURP can be monopolar or bipolar. In contrast to monopolar TURP, in bipolar TURP, the energy does not travel through the body to reach a skin pad. The circuitry is completed locally and the energy is confined between an active pole (resection loop) and a passive pole (situated on the resectoscope tip or on the sheath). Even if tissue removal is identical, bipolar TURP requires less energy and/or voltage because there is a smaller amount of interpolated tissue. Moreover, bipolar TURP uses isotonic saline as irrigation fluid (monopolar TURP typically uses glycine) and, hence, transurethral resection syndrome, which occurs with monopolar TURP, can be avoided in bipolar TURP. TURP is the treatment of choice for patients with a prostate volume of 30 ml–80 ml [8].

Open prostatectomy

OP, also known as simple prostatectomy, is the oldest and most invasive surgical approach for the treatment of LUTS secondary to BPO. It differs from radical prostatectomy, commonly performed for prostate cancer, because it only involves the enucleation of a hyperplastic prostatic adenoma, whereas the latter involves removal *en bloc* of the entire prostate, seminal vesicles and vas deferens. Usually, an incision is made through the lower abdomen or in the perineum. Obstructive adenomas are enucleated using the index finger, following different approaches (e.g., Freyer or Millin procedures). OP is one of the treatments of choice for patients with substantially enlarged prostates with volume >80 ml [8].

Bipolar enucleation of the prostate

Bipolar enucleation of the prostate, similar to all endoscopic enucleative approaches in general, was developed with the aim to perform an open prostatectomy endoscopically. The process involves completely resecting the transitional zone of the prostate using anatomical planes and morcellation of this tissue inside the bladder, to enable entirely endoscopic tissue extraction. Bipolar enucleation is known under different acronyms, such as BipolEP, TUEB and TuBE. It uses bipolar electrocautery provided through the tip of a resectoscope. A loop electrode is used in plasmakinetic enucleation of the prostate (PkEP), whereas bipolar plasma enucleation of the prostate (BPEP) is performed by a button electrode [9]. Bipolar enucleation is one of the treatments of choice for patients with substantially enlarged prostates with volume >80 ml [8].

Laser enucleation or vaporisation of the prostate

Several laser systems have been developed to perform both enucleation or vaporisation procedures, and other hybrid approaches, such as vaporesection, exist. HoLEP aims to perform endoscopic enucleation using the characteristics of a holmium:yttrium-aluminium garnet (Ho:YAG) laser that, with a wavelength of 2140 nm, is strongly absorbed by water and, thus, by the saline solution used during the endoscopic procedure as well as by the prostate tissue, given its high water content. The same laser can be also used to treat urinary tract stones, which are often encountered in patients with BPH [10]. HoLEP is one of the treatments of choice for patients with substantially enlarged prostates with volume >80 ml [8]. ThuVAP is performed by using a thulium:yttrium-aluminium-garnet laser (Tm:YAG) with a wavelength between 1940 and 2013 nm emitted in continuous wave mode and primarily used in front-fire applications. The same laser is used for other procedures ranging from vaporesection (thulium vaporesection of the prostate; ThuVaRP) to enucleation (thulium vapoenucleation of the prostate (ThuVEP)/ThuLEP). Diode lasers with a wavelength of 940, 980, 1318 or 1470 nm (depending on the semiconductor used) are marketed for procedures of vaporisation and enucleation of the prostate.

[A0020] – For which indications has LBO laser received CE marking?

As per the indication for use (IFU) document, GL-XPS is intended for the surgical incision and/or excision, vaporisation, ablation, haemostasis and coagulation of soft tissue. All soft tissue is included, such as skin, cutaneous tissue, subcutaneous tissue, striated and smooth tissue, muscle, cartilage meniscus, mucous membrane, lymph vessels and nodes, organs and glands (Table A6). The manufacturer states that, even if many procedures are possible within many specialties, GL-PS has only been used for urological applications (manufacturer questionnaire – see par. 2.3).

GL-XPS and GreenLight MoXy Optic Fibre received the CE mark April 2010 and June 2010, respectively. Approval from FDA for GL-XPS and GreenLight MoXy Optic Fibre was achieved in November 2009 and June 2010, respectively. In June 2012, the Joule limit of GreenLight MoXy Optic Fibre was extended from 400 kJ to 650 kJ (manufacturer questionnaire – see par. 2.3).

[B0002] – What is the claimed benefit of LBO laser PVP in relation to its comparators?

According to the manufacturer, GL-XPS would lead to shorter hospital length of stay (because the procedure can be done as a day-case procedure), shorter duration of catheterisation, quicker return to normal activity following treatment, reduction in pain leading to improved quality of life, can be used in patients taking anticoagulants and those with larger prostates, reduction in hospital readmissions, and reduced risk from capsular perforation, bleeding, and TURP syndrome (manufacturer questionnaire – see par. 2.3).

[B0003] – What is the phase of development of LBO laser PVP and its comparators?

The GreenLight laser platform evolved from the 80 W potassium-titanylphosphate (KTP) laser to the 120 W LBO laser (GreenLight HPS) and the current 180 W LBO laser system (GL-XPS) involving a MoXy liquid-cooled side-firing fibre [11].

Among the comparators, TURP (either monopolar or bipolar), TUIP and OP are the most well established, whereas endoscopic enucleation techniques are newer and require experience and relevant endoscopic skills [8].

[B0004] – Who administers LBO laser PVP and its comparators and in what context and level of care are they provided?

Either GL-XPS or its comparator procedures must be performed by trained urologists. The manufacturer states that, to perform a PVP procedure using GL- XPS, one urologist and one nurse are necessary. Training in the field for those units is provided by the manufacturer at no extra cost for the healthcare centre. Based on the manufacturer's experience, it takes from 20 to 25 cases (with the support of the product specialist) to be able to perform the procedure independently (manufacturer questionnaire – see par. 2.3). This information was also reported by the National Institute for Health and Care Excellence (NICE) in its Guidance [7]. However, a more recent study involving three operators from three different centres performing PVP using GL-XPS on 365 patients concluded that >100 procedures were required to reach an intraoperative parameter plateau regardless of surgeon expertise and institutional background. Both surgeon background and expertise appear to influence perioperative outcomes during the GL-XPS PVP learning curve [12].

The GL-XPS PVP procedure is performed within the urology department in a day-surgery (day-case procedure) or inpatient setting (manufacturer questionnaire – see par. 2.3).

[B0009] – What equipment and supplies are needed to perform LBO laser PVP?

Other than the GL-XPS Console and the single-use GreenLight MoXy Optic Fibre, a third element is necessary for the system: the fibre-stabilizing guide, also known as the laser bridge or working element. This device is specific for the GreenLight system and connects the MoXy Optic Fibre to the console, but it is not provided by the manufacturer, who recommends to use products provided by third parties (e.g., Karl Storz, Wolf and Olympus) (manufacturer questionnaire – see par. 2.3). Protective goggles are also required because GL-XPS uses a class IV laser.

[A0021] – What is the reimbursement status of LBO laser PVP?

We performed a survey across EUnetHTA WP4 partners and received replies from six partners only [Zorginstituut Nederland (ZIN), Institut für Qualität und Wirtschaftlichkeit im Gesundheitswesen

(IQWiG), Hauptverband der Österreichischen Sozialversicherungsträger (HVB), NICE, RER and Agencja Oceny Technologii Medycznych i Taryfikacji (AOTMiT)], one of which (ZIN) stated that they were unable to provide this information. We merged these replies with the information about the reimbursement status of the technology across European countries as provided by the manufacturer. Overall, we collected reimbursement information for Austria, England, France, Germany, Italy and Poland. Specific reimbursement codes have been issued only in Austria and Germany. In the other countries, the procedure is reimbursed under an umbrella code for transurethral prostatectomy, irrespective of the technology used.

4 HEALTH PROBLEM AND CURRENT USE OF THE TECHNOLOGY (CUR)

4.1 Research questions

Element ID	Research question
A0002	What is BPH?
A0003	What are the known risk factors for BPH?
A0004	What is the natural course of BPH?
A0005	What are the symptoms and the burden of BPH for the patient?
A0006	What are the consequences of BHP for society?
A0024	How is BPH currently diagnosed according to published guidelines?
A0025	How is BPH currently managed according to published guidelines?
A0007	What is the target population in this assessment?
A0023	How many people belong to the target population?
A0011	How much are LBO laser PVP and its comparators used?

4.2 Results

Overview of the disease or health condition

[A0002] – What is BPH?

BPH is a benign (noncancerous) condition with an adverse impact on the lower urinary tract function because of the hyperplasia and enlargement of the central transitional zone of the prostate. It is also called benign prostate enlargement (BPE). This can be caused by an increased rate of cell proliferation, reduced rate of apoptosis (cell death), or both [13].

[A0003] – What are the known risk factors for BPH?

Observational studies from Europe, USA and Asia have demonstrated older age to be a risk factor for clinical BPH onset and progression [14]. Given that the prostate volume increases with age (2.0%–2.5% per year in older men), continued prostate growth is a risk factor for LUTS progression, and larger prostates are associated with benign prostatic enlargement, increased risks of urinary retention and need for prostate surgery [15].

[A0004] – What is the natural course of BPH?

BPH becomes clinically significant when it starts contributing to bothersome LUTS [14]. BPH is a progressive disease and, left untreated, can lead to increased prostate volume, reduction in maximum urinary flow rate, and an increase in the risk of AUR, which is a medical emergency [16].

[A0005] – What are the symptoms and the burden of BPH for the patient?

BPH can lead to BPE, which obstructs the bladder outlet and is the most common cause of LUTS in men. These are best categorised into voiding, storage or postmicturition symptoms. Voiding symptoms include weak or intermittent urinary stream, straining, hesitancy, terminal dribbling and incomplete emptying. Storage symptoms include urgency, frequency, urgency incontinence and

nocturia. The major postmicturition symptom is postmicturition dribbling, which is common and bothersome. Although LUTS does not usually cause severe illness, it can considerably reduce men's quality of life, and can point to serious pathology of the urogenital tract. For these reasons, LUTS is a major burden for the ageing male population. Bothersome LUTS can occur in up to 30% of men older than 65 years. This is a large group potentially requiring treatment [17].

Effects of the disease or health condition

[A0006] – What are the consequences of BPH for the society?

BPH can impact the patient, their partner and also society. Estimates of disability-adjusted life year (DALY) for BPH have been calculated. DALY refers to the equivalent years of healthy life lost because of poor health or disability, with 1 DALY equating to 1 lost year of healthy life. According to the latest World Health Organization (WHO) estimates referring to the European region (data from 2016), BPH was responsible for 0.25% (~751,000) of the total DALYs caused by all conditions. By contrast, the proportion of DALYs attributable to other conditions, such as prostate cancer and hypertensive heart disease, is 0.71% and 0.87%, respectively [18]. In terms of economics, the impact of BPH is relevant considering the prevalence across the population, its specific diagnostic evaluation pathway, and subsequent medical and surgical management. Costs related to the management of complications related to BPH also need to be added [19]. A recent Spanish study of 610 patients reported estimates of median annual cost of €1070 per patient, including diagnostic tests and/or monitoring (54.6%), medical visits (20.5%) and treatment (29.6%), highlighting that the overall cost was higher in patients with a higher symptom score (€1127 versus €920; $P<0.001$) [20].

Current clinical management of the disease or health condition

[A0024] – How is BPH currently diagnosed according to published guidelines?

The latest European guidelines relevant to the present assessment are those published in 2017 by the EAU: *Management of Non-Neurogenic Male Lower Urinary Tract Symptoms (LUTS), incl. Benign Prostatic Obstruction (BPO)* [8] in which references used to provide recommendations are assessed according to their level of evidence, and grade of recommendation is expressed according to a classification system modified from the Oxford Centre for Evidence-Based Medicine Levels of Evidence [21]. The Guidelines focus on LUTS because they have traditionally been related to bladder outlet obstruction (BOO), which is often caused by BPE resulting from the histological condition of BPH [22,23]. Primary diagnostic evaluation of patients with LUTS involves medical history, symptom score questionnaires, urinalysis, physical examination and measurement of prostate-specific antigen (PSA) and PVR urine. In case of bothersome symptoms, the assessment includes also frequency volume charts (FVC) and bladder diaries, together with ultrasound assessment and uroflowmetry. This diagnostic evaluation pathway is synthesised in Table 4.1. For a more detailed and comprehensive presentation, please refer to the original source, which also provides a flow diagram [8].

Table 4.1. Diagnostic evaluation pathway of patients with bothersome LUTS[a]

Intervention	Recommendation	LE	GR
Medical history: to identify the potential causes and relevant comorbidities, including medical and neurological diseases, current medication, lifestyle habits, emotional and psychological factors	Take a complete medical history from men with LUTS	4	A
Symptom score questionnaires: to quantify LUTS and identify which type of symptoms are predominant (e.g., IPSS, ICIQ-MLUTS, DAN-PSS)	Use a validated symptom score questionnaire, including quality of life assessment, during the assessment of	3	B

Intervention	Recommendation	LE	GR
	male LUTS and for re-evaluation during and/or after treatment		
Urinalysis: to identify conditions such as urinary tract infections, microhaematuria and diabetes mellitus	Use urinalysis (by dipstick or urinary sediment) in assessment of male LUTS	3	A
Physical examination: to seek potential influences on LUTS, particularly focusing on the suprapubic area, external genitalia, perineum and lower limbs. Urethral discharge, meatal stenosis, phimosis and penile cancer must be excluded	Perform physical examination, including digital rectal examination, in the assessment of male LUTS	3	B
Prostate-specific antigen (PSA): to be used as predictor of prostate growth	Measure PSA if a diagnosis of prostate cancer will change management<br><br>Measure PSA if it assists in the treatment and/or decision-making process	1b<br><br>1b	A<br><br>A
Post-void residual (PVR) urine: to identify patients at risk of acute urinary retention	Measure postvoid residual in the assessment of male LUTS	3	B
Frequency volume charts (FVC) and bladder diaries: to derive day and night-time voiding frequency, total voided volume, nocturnal polyuria index and volume of individual voids	Use a bladder diary to assess male LUTS with a prominent storage component or nocturia<br><br>Tell the patient to complete a bladder diary for the duration of at least three days	3<br><br>2b	B<br><br>B
Ultrasound assessment: to perform simultaneous evaluation of bladder, PVR and prostate.	Perform ultrasound of upper urinary tract in men with LUTS and a large postvoid residual, haematuria or history of urolithiasis	3	B
Uroflowmetry: to determine Qmax and flow pattern	Uroflowmetry in initial assessment of male LUTS can be performed and should be performed before any treatment	2b	B

Abbreviations: DAN-PSS=Danish Prostate Symptom Score; GR=grade of recommendations; ICIQ-MLUTS=International Consultation on Incontinence Questionnaire; IPSS=International Prostate Symptom Score; LE=level of evidence.

[a]Based on Gravas et al. [8].

[A0025] – How is BPH currently managed according to published guidelines?

According to the latest European guidelines [8], the choice of treatment depends on the assessed findings of patient evaluation, the ability of the treatment to change the findings, the treatment preferences of the individual patient, and the expectations to be met in terms of speed of onset, efficacy, side effects, HRQoL and disease progression. Conservative or medical treatments are usually the first choice of therapy. Surgical treatment is usually required when patients have experienced recurrent or refractory urinary retention, overflow incontinence, recurrent UTIs, bladder stones or diverticula, treatment-resistant macroscopic haematuria because of BPH and/or BPE, or dilatation of the upper urinary tract because of BPO, with or without renal insufficiency. These represent absolute operation indications. Additionally, surgery is usually needed when patients have not obtained adequate relief from LUTS or PVR using conservative or medical treatments (relative operation indications). The choice of surgical technique depends on prostate size, comorbidities of the patient, ability to have anaesthesia, patient preferences, willingness to accept surgery-associated specific side-effects, availability of the surgical armamentarium and experience of the surgeon with these surgical techniques. The recommendations on the use of LBO laser for PVP are presented in Table 4.2. For recommendations on all the treatment options for those patients with LUTS having absolute indications for surgery or nonresponders to medical treatment or those not willing to undergo medical treatment but requesting active treatment, please refer to the original source [8] [24], which also provides a flow diagram. Many of the studies on which the recommendations are based were performed using the previous generations of the current LBO laser system (in particular, the 80 W KTP

laser and the 120 W LBO laser GreenLight HPS) and results should be interpreted accordingly. At the time of writing, the 180 W GL-XPS should be regarded as the reference [8].

Guidelines published by the American Urological Association (AUA) [25] and the Canadian Urological Association (CUA) [26] are essentially based on the same trial, the GOLIATH Study. Although the strength of recommendations from CUA are in line with those from EAU (rated as 'strong'), those from AUA are rated as 'moderate'.

Table 4.2. Recommendations for the use of LBO laser for PVP

Final recommendations[a]	Strength rating[b]
Offer 80-W 532-nm[c] KTP laser vaporisation of the prostate to men with moderate-to-severe LUTS as an alternative to TURP	Strong
Offer 120-W 532-nm[c] LBO laser vaporisation of the prostate to men with moderate-to-severe LUTS as an alternative to TURP	Strong
Offer 180-W 532-nm[c] LBO laser vaporisation of the prostate to men with moderate-to-severe LUTS as an alternative to TURP	Strong
Offer laser vaporisation of the prostate using 80-W KTP, 120- or 180-W LBO lasers for the treatment of patients receiving antiplatelet or anticoagulant therapy with a prostate volume <80 ml	Weak

Abbreviations: KTP=kalium-titanyl-phosphate; LBO=lithium triborate; LUTS=low urinary tract symptoms; PVP=photoselective vaporisation of the prostate; TURP=transurethral resection of the prostate.

[a]Based on Gravas et al. [24].

[b]The strength of each recommendation is represented by the words 'strong' or 'weak'. The strength of each recommendation is determined by the balance between desirable and undesirable consequences of alternative management strategies, the quality of the evidence (including certainty of estimates), and the nature and variability of patient values and preferences [24].

[c]Within the source document, the term '532-nm laser' is used to refer to the GreenLight laser system.

Target population

[A0007] – What is the target population of this assessment?

For the present assessment, the target population was defined as specific groups of the general population of patients presenting with bothersome LUTS because of BPH and/or BPE having absolute indications for surgery or nonresponders to medical treatment or not willing to undergo medical treatment but requesting active treatment, according to the latest European guidelines [8] and taking in account the advice received by the external clinical experts involved in the assessment.

Within the present assessment, target populations were defined as men with indication for surgical treatment of BPH and:

- prostate volume <30 ml;
- prostate volume between 30 and 80 ml;
- prostate volume >80 ml;
- at risk of bleeding sequelae who cannot stop anticoagulation therapy.

Recommended treatment options differ among these four groups, and the different comparators are detailed in the PICO. Nevertheless, the technology under assessment (LBO laser PVP performed using GL-XPS) has been acknowledged as a potential treatment option for each of the four groups.

[A0023] – How many people belong to the target population?

Accurate prevalence estimates for the four groups mentioned earlier (i.e., those for which LBO laser PVP performed using GL-XPS has been acknowledged as a potential treatment option) have not been identified. Global prevalence of BPH was estimated by a recent meta-analysis [27] that included 30 epidemiological studies from 25 countries. The number of participants per study varied considerably (from 288 to 26,446). Even if a high level of heterogeneity was observed, the authors of the meta-analysis calculated a lifetime prevalence of BPH of 26.2% (16,437/76,246 individuals; 95% CI: 22.8%–29.8%). No statistically significant difference in prevalence estimates were noted when data were stratified between rural, urban or mixed populations. Of the total 30 studies, only 25 studies reported age-specific stratified data. Pooled prevalence estimates increased with age from 14.8% in the age group 40–49 years, 20% in the age group 50–59 years, 29.1% in the age group 60–69 years, 36.8% in the age group 70–79 years, and 38.4% in the age group 80 years and above. However, the level of heterogeneity was high. The authors concluded that some heterogeneity was probably because of methodological differences across the different studies and different definitions of BPH.

[A0011] – How much are the LBO laser PVP and its comparators used?

The manufacturer states that, in 2017, ~754 centres were active across Europe (~2750 around the world). A total of 30,372 procedures were performed in Europe during 2017 (182,382 worldwide). In the timespan 2014–2017, 368,229 procedures were performed worldwide (manufacturer questionnaire – see par. 2.3). Accurate estimates of the usage of the different comparators were not identified. TURP has been the undisputed reference standard for the management of BPO over the decades. During the past two decades, its role has been increasingly challenged by the development of less invasive options, such as laser procedures [28].

5 CLINICAL EFFECTIVENESS (EFF)

5.1 *Research questions*

Element ID	Research question
D0005	How does LBO laser PVP affect:
D0006	How does LBO laser PVP affect progression of BPH?
D0011	What is the effect of LBO laser PVP on patient bodily functions (urination and sexual function)?
D0016	How does LBO laser PVP affect activities of daily living?
D0012	What is the effect of LBO laser PVP on generic health-related quality of life?
D0013	What is the effect of LBO laser PVP on disease-specific quality of life?
D0017	Were patients satisfied with LBO laser PVP?

5.2 *Results*

Included studies

We identified five publications from three randomised trials enrolling 434 participants [1–5].

Two trials compared GL-XPS with TURP, the GOLIATH Study [1–3] and Jovanović et al. [4]. The GOLIATH Study [1–3] had a non-inferiority design and was performed in 29 centres in nine European countries, whereas the study by Jovanović et al. [4] was performed in one centre in Belgrade. The mean age in both studies ranged between 66 and 67 years. Both studies had the following inclusion criteria: medical record documentation of Qmax <15 ml/s and prostate volume <100 ml on TRUS. However, whereas the GOLIATH Study [1–3] required an IPSS ≥12, Jovanović et al. [4] required an IPSS >16. In addition, Jovanović et al. [4] excluded patients who were taking permanent oral anticoagulation treatment, whereas the GOLIATH Study [1–3] did not have this exclusion criterion; however, only 5% of the participants were taking anticoagulation treatment. The basic characteristics of participants in both studies are shown in Table 2 and Table A1.

In the GOLIATH Study [1–3], the primary outcomes assessed with a non-inferiority approach were IPSS score and Qmax (ml/s), with pre-set non-inferiority margins of three points and –5 ml/s, respectively; in addition, the proportion of complication-free participants was evaluated, with a non-inferiority margin of –5%. However, the following critical outcomes for effectiveness were assessed by the GOLIATH Study [1–3]: improvement of QoL using the IPSS-QoL score; improvement in PVR volume; rate of dysuria; and patient-reported outcomes (e.g., sexual function, disease-specific quality of life, etc.). Safety outcomes were rate of re-intervention, urinary incontinence, irritative symptoms, erectile dysfunction, and urethral and bladder neck strictures.

Jovanović et al. [4], in addition to IPSS and Qmax, evaluated the following critical effectiveness outcomes: PVR volume (data not showed) and rate of dysuria; safety critical outcomes were urinary incontinence and strictures. Follow-up periods were 6, 12 and 24 months for the GOLIATH Study [1–3], and 1, 3, 6 and 12 months for Jovanović et al. [4].

The third trial [5] had a non-inferiority design and compared vapoenucleation of the prostate with the GL-XPS with HoLEP in 103 participants with BPH. The trial was conducted in a single centre in Canada. Inclusion criteria were patient age >50 years, refractory LUTS secondary to BPH, IPSS >15, QoL score 3 or more, Qmax <15 ml/s, or patients with acute urinary retention secondary to BPH in whom trial of voiding failed, and prostate size on preoperative TRUS of 40 ml–150 ml. Maximum follow-up was 12 months. The only primary outcome was IPSS score, and the pre-set non-inferiority margin was three points. The basic characteristics of participants and the secondary and safety outcomes considered are illustrated in Table 2 and Table A1.

Both the GOLIATH and Elshal et al. studies used the same non-inferiority margin of three points for IPSS, referring to two papers by Barry et al. [29,30].

Risk of bias assessment

All included trials were randomised. An appropriate randomisation process was performed in the GOLIATH Study [1–3] and the study by Elshal et al. [5], however, the allocation concealment was properly performed only in the GOLIATH Study [1–3] because the patients were assigned to treatments by a permuted-block randomisation schedule with mixed-block sizes of two and four and the assignment was performed using sequentially numbered and sealed envelopes that contained the random treatment assignment; in the Elshal study [5], the methods used to conceal the allocation were not described. Jovanović et al. [4] did not report any description regarding the method used to generate the random sequence or the methods used to conceal allocation.

Given technological and procedural differences between GL-XPS and its comparators, blinding of participants and personnel was not performed in any of the included studies. Thus, all the studies were considered at risk of performance bias for subjective outcomes, whereas, for objective outcomes, we assumed that the risk of performance bias was unlikely. In the GOLIATH Study [1–3], adverse events were adjudicated by an independent external clinical events committee of three board-certified academic urologists, and this could have mitigated the performance bias for these safety outcomes.

Similarly, subjective outcomes are at higher risk of detection bias compared with objective outcomes; none of the studies reported sufficient detail for judgement.

The study by Elshal et al. [5] was considered at risk of attrition bias because of the exclusion from analyses of 5/55 patients in the HoLEP group, compared with none excluded in the GL-XPS group; intention-to-treat analysis was not performed. No concerns were reported in terms of attrition bias in the other two trials.

In terms of selective outcome reporting, Jovanović et al. [4] was considered at high RoB because they did not report the results for all prespecified time points or the standard deviations for several continuous data. The corresponding author also failed to answer our request for information. We also judged Elshal et al. [5] to be at high risk of selective reporting bias because the authors did not report any follow-up data for the outcome ‘erectile function’. No concern for selective reporting was found in the GOLIATH Study [13] (Figure 5.1).

The GOLIATH Study was funded by AMS; we did not consider automatically this sponsorship as source of bias.

RoB tables for study level and outcome level are reported in Appendix 1, Table A2 and Table A3.

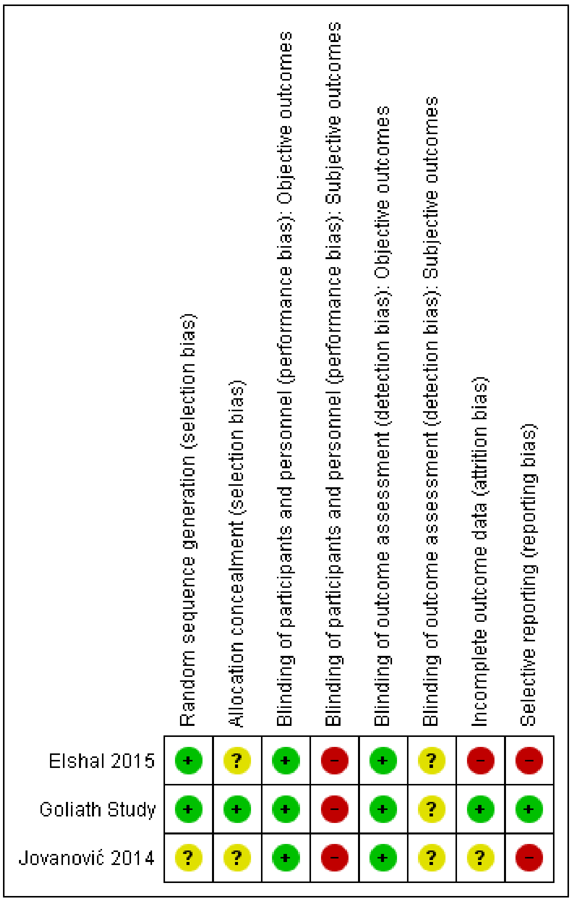

Figure 5.1. Assessment of risk of bias in included studies

Clinical effectiveness

[D0005] – How does the LBO laser PVP affect: reduction in BPH symptoms; change in maximum flow rate (Qmax), and postvoid residual volume (PVR); the rate of dysuria (pain); duration of catheterisation; and length of hospital stay?

GL-XPS versus TURP

Reduction of symptoms using the IPSS score

This outcome was evaluated by both the GOLIATH Study [1–3] and Jovanović et al. [4]. However, it was not possible to pool the data because one of the two trials [4] did not provide standard deviations for the reported mean values.

In the GOLIATH Study [1–3], non-inferiority of GL-XPS versus TURP in the reduction of symptoms was shown at 6-month (MD 1.2, 95% CI –0.0 to 2.4), 12-month (MD 1.2, 95% CI –0.2 to 2.6) and 24-month follow-up (MD 1.0, 95% CI –0.5 to 2.5).

In Jovanović et al. [4], we assumed that the data presented referred to the maximum follow-up time (12 months). At 12 months, there was no evidence of difference in IPSS score between the two groups (MD 0.4, CI not reported).

We downgraded the quality by two levels for this outcome owing to RoB (performance, detection and selection bias) and imprecision. Certainty on estimates provided by both studies was judged as low, meaning that further good-quality randomised studies could change both the size and direction of effect.

Improvement in Qmax

This outcome was evaluated by both the GOLIATH Study [1–3] and Jovanović et al. [4]. However, it was not possible to pool the data because Jovanović et al. [4] did not provide standard deviations for the reported mean values.

In the GOLIATH Study [1–3], non-inferiority of GL-XPS versus TURP in Qmax was shown at 6-month (MD –1.0, 95% CI –3.6 to 1.6), 12-month (MD –1.8, 95% CI –4.3 to 0.7) and 24-month follow-up (MD –1.3, 95% CI –3.8 to 1.2].

In Jovanović et al. [4], we assumed that the data presented referred to the maximum follow-up time (12 months). However, there was no evidence of difference between the two groups (MD 0.2, CI not reported).

We downgraded by one level the quality for this outcome at 24 months because of imprecision. We downgraded by two levels the quality for this outcome at 6 and 12 months because of RoB (selection and selective outcome reporting bias) and imprecision.

Hence, there is moderate certainty that GL-XPS is non-inferior to TURP at 24-month follow-up in improving Qmax (GRADE evidence: moderate), whereas certainty about the non-inferiority of GL-XPS is lower at 6- and 12-month follow-up (GRADE evidence: low), meaning that further good-quality randomised studies could change both the size and direction of effect.

Reduction in PVR volume

This outcome was evaluated by both the GOLIATH Study [1–3] and Jovanović et al. [4]. However, it was not possible to pool the data because Jovanović et al. [4] did not report the postoperative PVR values.

In the GOLIATH Study [1–3], the mean reduction in PVR volume was similar between GL-XPS and TURP at 6-month follow-up (MD 3.8, 95% CI –8.4 to 16.0), 12 months (MD 9.4, 95% CI –3.1 to 21.9) and 24-month follow-up (MD 10.7, 95% CI –3.5 to 24.9).

We downgraded by one level the quality for this outcome at 24 months because of imprecision. We downgraded by two levels the quality for this outcome at 6 and 12 months because of RoB (selection and selective outcome reporting bias) and imprecision.

Hence, there is moderate certainty that GL-XPS and TURP do not differ in reducing the PVR volume at 24-month follow-up (GRADE evidence: moderate), whereas, at 6- and 12-month follow-up, such a conclusion has low certainty (GRADE evidence: low). This means that further good-quality randomised studies could change the size (and in some cases direction) of effect.

Rate of dysuria

Rate of dysuria was reported by both the GOLIATH Study [1–3] and Jovanović et al. [4]. At 12-month follow-up, 9.6% of the patients in the GL-XPS group and 12.2% in the TURP group had dysuria. The proportion of this outcome was similar between the two groups at 12 months (RR 0.81, 95% CI 0.45 to 1.45; Figure 5.2). We downgraded by three levels the quality for this outcome because of serious concern regarding RoB (selection, performance, detection, and selective reporting bias) and very serious concern in terms of imprecision (GRADE evidence: very low). This means that the estimate of effect is very uncertain and confidence in the estimate is small.

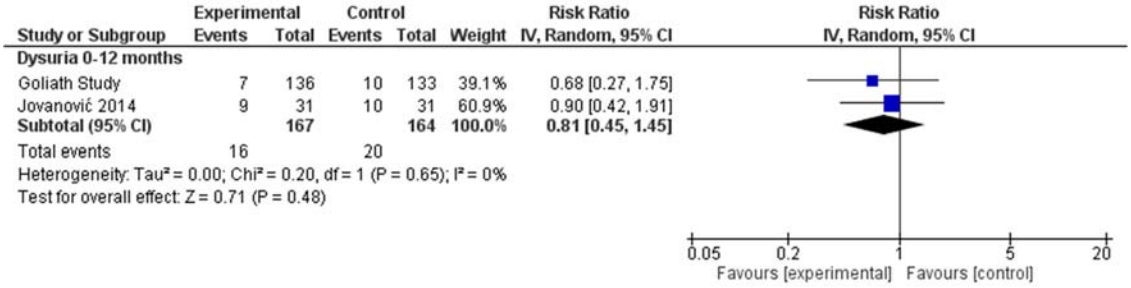

Figure 5.2. Rate of dysuria reported by the GOLIATH and Jovanović et al. [4] studies

Duration of catheterisation

This outcome was evaluated by both the GOLIATH Study [1–3] and Jovanović et al. [4]. In both studies, length of catheterisation was significantly shorter in the GL-XPS group than in the TURP group (MD –32 h, 95% CI –56 to –8; Figure 5.3). We downgraded the quality of this outcome because of serious concerns regarding RoB and imprecision (GRADE evidence: very low). This means that the estimate of effect is very uncertain and confidence in the estimate is small.

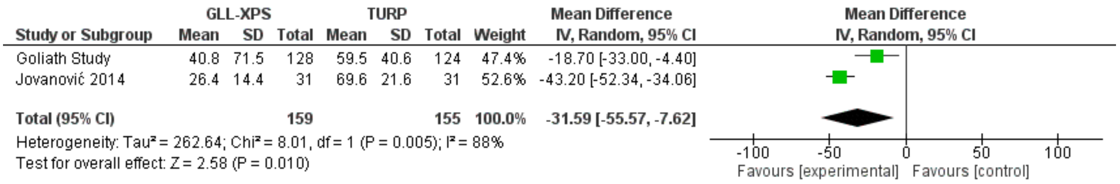

Figure 5.3. Duration of catheterisation reported by the GOLIATH and Jovanović et al. [4] studies

Length of hospital stay

This outcome was evaluated by both GOLIATH Study [1–3] and Jovanović et al. [4]. In both studies, length of hospital stay was significantly shorter in the GL-XPS group than in TURP group (MD –46 h, 95% CI –74 to –18; I^2 90%; Figure 5.4). We downgraded the quality of this outcome because of serious concerns regarding RoB and imprecision (GRADE evidence: very low). This means that the estimate of effect is very uncertain and confidence in the estimate is small.

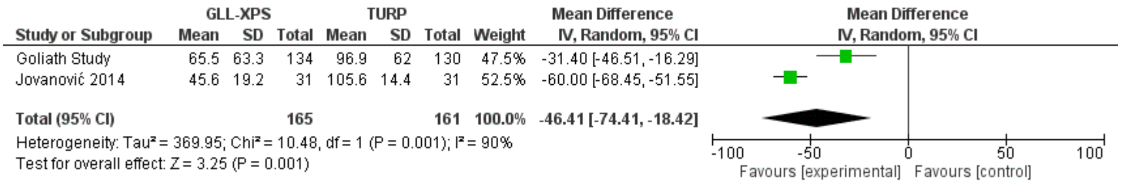

Figure 5.4 Length of hospital stay reported by the GOLIATH and Jovanović et al. [4] studies

GL-XPS versus HoLEP

The only trial included comparing vapoenucleation of the prostate with GL-XPS and HoLEP was that reported by Elshal et al. [5].

Reduction of symptoms using the IPSS score

In Elshal et al. [5], based on the IPSS score, GL-XPS was non-inferior to HoLEP in reducing symptoms at 12 months (MD 1.0, 95% CI –1.0 to 3.0) and at 1 month (MD –2.4, 95% CI –4.8 to –0.1), whereas, at 4 months, non-inferiority of GL-XPS versus HoLEP was not shown (MD 3.6, 95% CI 1.4 to 5.8).

We downgraded by three levels the quality for this outcome because of RoB (selection and detection bias suspected; performance and attrition bias) and very serious imprecision. Hence, there is very low certainty about the non-inferiority of GL-XPS compared with HoLEP in terms of the reduction in symptoms using IPSS score at 12-month follow-up (GRADE evidence: very low). This means that the estimate of effect is very uncertain and confidence in the estimate is small.

Improvement in Qmax

At 12-month follow-up, HoLEP improved Qmax significantly better than GL-XPS (MD 17.1 ml/s, 95% CI 11.6 to 22.6), and the same occurred at 4 months (MD 6.5 ml/s, 95% CI 1.8 to 11.2). At 1 month follow-up there was no evidence of difference between GL-XPS and HoLEP (MD -4.3 ml/s, 95% CI -8.6 to 0.0).

We downgraded by three levels the quality for this outcome because of RoB (possible selection bias; attrition bias) and very serious imprecision (GRADE evidence: very low). Very low-quality evidence means that any estimate of effect is very uncertain and confidence in the estimate is small.

Reduction of PVR volume

There was no evidence of difference in the reduction of PVR volume between GL-XPS and HoLEP at 1-month (MD 14.0, 95% CI –9.6 to 37.6), 4-month (MD 20.0, 95% CI –13.4 to 53.4) and 12-month follow-up (MD 27.0, 95% CI –0.7 to 54.7).

We downgraded by three levels the quality for this outcome because of RoB (possible selection bias; attrition bias) and very serious concern regarding imprecision (GRADE evidence: very low). Very low-quality evidence means that any estimate of effect is very uncertain and confidence in the estimate is small.

Rate of dysuria and dysuria visual analogue scale

Two (4%) patients had postoperative dysuria in the GL-XPS group versus no patients in the HoLEP group (RR=4.72, 95% CI 0.23 to 96.01; *P*=0.31). Elshal et al. [5] also evaluated dysuria using a visual analogue scale (VAS) at 1 month, showing no evidence of difference between the GL-XPS and HoLEP groups (MD –0.50, 95% CI –1.26 to 0.26).

We downgraded by three levels the quality for this outcome because of RoB (possible selection and detection bias; performance and attrition bias) and very serious imprecision (GRADE evidence: very low). Very low-quality evidence means that the estimate of effect is very uncertain and confidence in the estimate is small.

Duration of catheterisation

Length of catheterisation was significantly shorter in the HoLEP group than in the GL-XPS group (MD –26 h, 95% CI –52 to –1) (GRADE evidence: very low). However, very low-quality evidence means that the estimate of effect is very uncertain and confidence in the estimate is small.

Length of hospital stay

There was no evidence of difference between length of hospital stay in the GL-XPS group and HoLEP group (MD 9.6 h, 95% CI –0.0 to 19.2; *P*=0.056) (GRADE evidence: very low). Very low-quality evidence means that the estimate of effect is very uncertain and confidence in the estimate is small.

[D0006] – How does LBO laser PVP affect progression of BPH?

GL-XPS versus TURP and GL-XPS versus HoLEP

We did not find any evidence to answer this research question.

[D0011] – What is the effect of LBO laser PVP on patient bodily functions (urination and sexual function)?

GL-XPS versus TURP

Patient-reported outcomes

We downgraded by two levels the quality for all the patient-reported outcomes listed below because of RoB (performance bias and detection bias) and imprecision (GRADE evidence: low). Low-quality evidence means that further research is likely to change the size and direction of effects and confidence in the estimate is limited.

Erectile function (IIEF-5)

Erectile function assessed using the (IIEF-5 score was evaluated only by the GOLIATH Study [1–3]. There was no evidence of difference between GL-XPS and TURP at 12-month (MD –1.3, 95% CI –3.3 to 0.7) and 24-month follow-up (MD –1.0, 95% CI –3.0 to 1.0).

Overactive bladder symptoms and health-related quality of life

These outcomes were assessed only by the GOLIATH Study [1–3] using the OABq-SF Symptom, OABq-SF Health, and the ICIQ-UI SF).

TURP was better than GL-XPS in terms of OABq-SF Symptom score at 6 months (MD 5.1, 95% CI 1.5 to 8.7), whereas no difference was observed at 12 (MD 4.0, 95% CI 0.0 to 8.0) and 24 months (MD 3.4, 95% CI –0.4 to 7.2).

TURP was better than GL-XPS in terms of OABq-SF Health score at 6 months (MD 3.6, 95% CI 0.0 to 7.2) and 12 months (MD 4.3, 95% CI 0.5 to 8.1), whereas no difference was observed at 24 months (MD 2.6, 95% CI –1.1 to 6.3).

Incontinence-related symptoms

In the GOLIATH Study [1–3], TURP was superior to GL-XPS in self-reported incontinence (ICIQ-UI SF) at 6 months (MD –1.3, 95% CI –2.2 to –0.5) and at 12 months (MD –1.2, 95% CI –2.2 to –0.2), whereas, at 24 months, there was no difference (MD -0.8, 95% CI –1.7 to 0.1).

Others

Three other questionnaires were evaluated [EuroQol-5D (EQ-5D), Short Form (36) Health Survey (SF-36) Mental Health and SF-36 Physical Health) in the GOLIATH Study [1–3] but showed no evidence of difference between GL-XPS and TURP groups in the three follow-up periods.

GL-XPS versus HoLEP

Patient-reported outcomes: erectile function (IIEF-15)

Erectile function measured by the IIEF-15 score was an outcome declared in the methods by Elshal et al. [5] but they did not report any parallel comparison in the results section.

Health-related quality of life

[D0016] – How does LBO laser PVP affect activities of daily living?

[D0012] – What is the effect of LBO laser PVP on the generic health-related quality of life?

[D0013] – What is the effect of LBO laser PVP on the disease-specific quality of life?

GL-XPS versus TURP

Improvement of HRQoL using the IPSS-QoL score

The improvement of HRQoL using the IPSS-QoL score was evaluated only by the GOLIATH Study [1–3]. There was no evidence of difference between GL-XPS and TURP in IPSS-QoL score at 6-month follow-up (MD 0.3, 95% CI –0.0 to 0.6), 12 months (MD 0.2, 95% CI –0.1 to 0.5) and 24-month follow-up (MD 0.1, 95% CI –0.2 to 0.4).

We downgraded by two levels the quality for this outcome because of RoB (performance bias and detection bias) and imprecision (GRADE evidence: low). Low-quality evidence means that further research is likely to change both the size and direction of effect, and confidence in the estimate is limited.

GL-XPS versus HoLEP

Improvement of QoL using the IPSS-QoL score

There was no evidence of a difference between GL-XPS and HoLEP in IPSS-QoL score at 1 month (MD –0.5, 95% CI –1.1 to 0.1), 4 months (MD 0.0, 95% CI –0.6 to 0.6) and 12-month follow-up (MD 0.1, 95% CI –0.4 to 0.6).

We downgraded by three levels the quality for this outcome because of RoB (selection and detection bias suspected; performance and attrition bias) and very serious imprecision (GRADE evidence: very low). Very low-quality evidence means that the estimate of effect is very uncertain and confidence in the estimate is small.

Patient satisfaction

[D0017] – Were the patients satisfied with LBO laser PVP?

GL-XPS versus TURP

In the GOLIATH Study [1–3], patient satisfaction with the procedures was assessed through surveys. At the end of a 2-year follow-up, patient satisfaction with their treatment was measured by willingness to undergo the therapy again (93% in the GL-XPS and 89% in the TURP group), and willingness to recommend their therapy (93% in the GL-XPS and 91% in the TURP group); the results were similar between the two techniques. These results were also comparable at 6-month and 12-month follow-up.

GL-XPS versus HoLEP

We did not find any evidence to answer this research question.

6 SAFETY (SAF)

6.1 *Research questions*

Element ID	Research question
C0008	How safe is LBO laser PVP compared with its comparators in terms of: rate of re-intervention; established urinary incontinence; irritative symptoms; mortality, procedural blood loss and blood transfusion need; rate of TURP syndrome; rate of capsular perforation; and any procedure or device-related adverse events?
C0002	How do the harms relate to dosage or frequency of applying LBO laser?
C0004	How does the frequency or severity of harms change over time or in different settings?
C0005	What are the susceptible patient groups that are more likely to be harmed through the use of LBO laser PVP?
C0007	Are LBO laser PVP and its comparators associated with user-dependent harms?

6.2 *Results*

Included studies

Studies included in the safety analysis are the same as those described in the EFF domain. The characteristics of the studies are shown in Table 2 and in more detail in Appendix 1, Table A1.

Patient safety

[C0008] – How safe is LBO laser PVP in relation to the comparators in terms of: rate of re-intervention; established urinary incontinence; irritative symptoms; mortality, procedural blood loss and blood transfusion need; rate of TURP syndrome; rate of capsular perforation; and any procedure or device-related adverse events?

GL-XPS versus TURP

Re-intervention rate

The rate of re-intervention for any cause was assessed in the GOLIATH Study [1–3] at 30-day, 6-month and 12-month follow-up. At 30 days, the patients in the GL-XPS group had a significantly lower rate of re-intervention compared with patients in TURP group (RR=0.30, 95% CI 0.10 to 0.90; *P*=0.03). However, this difference was not statistically significant at 6 months (RR=0.71, 95% CI 0.36 to 1.38; *P*=0.31) or 12 months (RR=0.78, 95% CI 0.42 to 1.44; *P*=0.43).

We downgraded by two levels the quality for this outcome because of serious imprecision because of a wide CI (GRADE evidence: low). Low-quality evidence means that further research is likely to change the size and direction of effect and confidence in the estimate is limited.

Surgical retreatments for obstruction

The rate of surgical retreatments for obstructions was assessed in the GOLIATH Study [1–3]. During the first 6 months, four (3%) patients in the GL-XPS group and seven (5%) patients in the TURP group were retreated (*P*=0.34). At the end of the first year from intervention, there were ten (7%) retreatments in the GL-XPS group and nine (7%) in the TURP group (p=0.85). At 24-month follow-up, 14 patients in the GL-XPS group versus ten patients in the TURP group (RR 1.37, 95% CI 0.63 to 2.97; *P*=0.42) had had another surgical intervention. The most common reasons for re-intervention were bladder neck contracture (48%), urethral stricture (33%) and prostate tissue regrowth (17%).

We downgraded by two levels the quality for this outcome because of serious imprecision resulting from a wide CI (GRADE evidence: low). Low-quality evidence means that further research is likely to change both the size and direction of effect and that confidence in the estimate is limited.

Urinary incontinence

This outcome was assessed by both the GOLIATH Study [1–3] and Jovanović et al. [4]. It was not possible to pool data because Jovanović et al. [4] only stated that no urinary incontinence episodes occurred post operatively, without referring to a specific follow-up.

In the GOLIATH Study [1–3], the adverse events were classified using the Clavien-Dindo scale (Grade I–V). In the first 6 months, overall, 14 (10%) patients in the GL-XPS group and six (5%) patients in the TURP group experienced urinary incontinence (*P*=0.08). Between the 7th and 12th months, only two (1.5%) patients in the GL-XPS group had urinary incontinence versus no patients in the TURP group (*P*=0.3). Finally, no incontinence episodes were registered during the second year of follow-up.

We downgraded by two levels the quality for this outcome because of serious imprecision resulting from a wide CI (GRADE evidence: low). Low-quality evidence means that further research is likely to change the size and direction of effect.

Irritative symptoms

Irritative symptoms, including pain and discomfort, were assessed only in the GOLIATH Study [1–3]. During the first 6 months, overall 27 (20%) patients in the GL-XPS group and 29 (22%) patients in the TURP group experienced irritative symptoms (*P*=0.69). Between the 7th and 12th months, three (2.2%) patients in the GL-XPS group had irritative symptoms versus one patient in the TURP group (*P*=0.35). During the second year of follow-up, only one patient in the GL-XPS group reported irritative symptoms, compared with no patients in the TURP group (*P*=0.51).

We downgraded by two levels the quality for this outcome because of serious imprecision resulting from a wide CI (GRADE evidence: low). Low-quality evidence means that further research is likely to change both the size and direction of effect.

Strictures

This outcome was assessed by both the GOLIATH Study [1–3] and Jovanović et al. [4].

In the GOLIATH Study [1–3], strictures included meatal, urethral, and bladder neck strictures. During the first 6 months, overall, four (3%) patients in the GL-XPS group and six (5%) patients in the TURP group experienced strictures (*P*=0.5). Between the 7th and 12th month, four (3%) patients in the GL-XPS group had strictures versus three (2%) patients in the TURP group (P=0.7). During the second year of follow-up, only one patient in the GL-XPS group reported strictures, compared with no patients in the TURP group (*P*=0.5).

Jovanović et al. [4] stated that, during the follow-up period (0–12 months), one patient (3%) in the GL-XPS group and four patients (13%) in the TURP group developed bladder neck contracture (*P*=0.2), whereas no patients in either group had urethral stricture.

We pooled data for the outcome strictures at 12-month follow-up: there was no evidence of difference between GL-XPS and TURP (RR 0.69, 95% CI 0.26 to 1.79; *P*=0.44; Figure 6.1). We downgraded by three levels the quality for this outcome because of RoB (selection bias suspected; selective outcome reporting) and very serious imprecision resulting from a wide CI (GRADE evidence: very low). Very low-quality evidence means that the estimate of effect is very uncertain and confidence in the estimate is small.

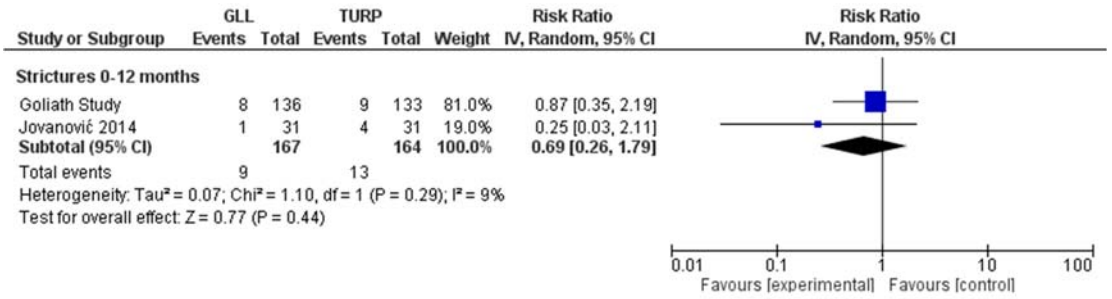

Figure 6.1. Strictures experienced in the 0–12-month timeframe reported by the GOLIATH and Jovanović et al. [4] studies

Urinary tract infection

UTI was assessed only in the GOLIATH Study [1–3]. During the first 6 months, overall, 24 (18%) patients in the GL-XPS group and 14 (11%) patients in the TURP group experienced UTI (*P*=0.1). During the 7–12-months and 13–24-month follow-ups, two (1.5%) patients in the GL-XPS group had UTIs versus no patients in the TURP group (*P*=0.3) during each period. Overall, at the 24-month follow-up, the risk of UTI was statistically significant higher in the GL-XPS group than in the TURP group (RR 1.96, 95% CI 1.08 to 3.55).

We downgraded by two levels the quality for this outcome because of serious imprecision resulting from a wide CI (GRADE evidence: low). Low-quality evidence means that further research is likely to change both the size and direction of effect and confidence in the estimate is limited.

Urinary retention

Urinary retention was assessed only in the GOLIATH Study [1–3]. During the first 6 months, overall, 15 (11%) patients in the GL-XPS group and 13 (10%) patients in the TURP group experienced urinary retention (*P*=0.7). During the 7–12-month follow-up, two (1.5%) patients in the GL-XPS group had urinary retention versus no patients in the TURP group (*P*=0.3). During the second year of follow-up, only one patient in both the GL-XPS and TURP groups had urinary retention (*P*=0.99). Overall, at the 24-month follow-up, there was no evidence of a difference between GL-XPS and TURP (RR 1.26, 95% CI 0.65 to 2.42).

We downgraded by two levels the quality for this outcome because of serious imprecision resulting from a wide CI (GRADE evidence: low). Low-quality evidence means that further research is likely to change both the size and direction of effect and confidence in the estimate is limited.

Erectile dysfunction

Erectile dysfunction was reported as an adverse event only in the GOLIATH Study [1–3]. During the first 12 months no episodes occurred in either group, whereas, during the second year of follow-up, one patient in the TURP group had worsening of erectile function (Grade I on the Clavien-Dindo scale) versus no patients in the GL-XPS group (p=0.5).

We downgraded by three levels the quality for this outcome because of RoB (performance bias) and serious imprecision resulting from a wide CI (GRADE evidence: very low). Very low-quality evidence means that the estimate of effect is very uncertain and confidence in the estimate is small.

Procedural transfusions

Procedural transfusions were reported by both the GOLIATH Study [1–3] and Jovanović et al. [4]. Patients in the GL-XPS group had a lower risk to be transfused during surgical procedure compared with TURP patients (RR=0.12, 95% CI 0.02 to 0.97).

Bleeding (procedural and post operative)

Procedural and postoperative bleeding was evaluated only in the GOLIATH Study [1–3]. During the first 6 months, there were 15 (11%) bleeding episodes in the GL-XPS group and 22 (17%) in TURP group (*P*=0.19). Between 7th and 12th months, one patient had bleeding in the GL-XPS group versus no patients in TURP group (*P*=0.5), whereas no episodes occurred in either group during the second-year follow-up.

TURP syndrome

TURP syndrome was reported only by Jovanović et al. [4], who described one (3%) episode in the TURP group versus none in the GL-XPS group *(P*=0.5).

Rate of capsular perforation

The rate of capsular perforation was reported only by Jovanović et al. [4], who described five (16%) episodes in the TURP group versus none in the GL-XPS group (*P*=0.1).

GL-XPS versus HoLEP

Elshal et al. [5] (the only study included for this comparison) described the following safety outcomes:

Re-intervention rate

Post operatively, three (6%) patients in the GL-XPS group and two (4%) patients in the HoLEP group were retreated (RR 1.42, 95% CI 0.25 to 8.12; *P*=0.7). The reasons for re-intervention in the GL-XPS group were postoperative haematuria (one patient) and residual prostatic adenoma (two patients) while in the HoLEP group were postoperative haematuria (one patient) and bladder neck contracture (one patient).

We downgraded by three levels the quality for this outcome because of RoB (selection bias and detection bias suspected; attrition bias) and very serious imprecision (GRADE evidence: very low). Very low-quality evidence means that the estimate of effect is very uncertain and confidence in the estimate is small.

Urinary incontinence

Urinary incontinence occurred at 1 month in eight (15%) patients in the GL-XPS group and in seven (14%) patients in the HoLEP group (*P*=0.9), and at 3 months in one (2%) patient in the GL-XPS group and in five patients (10%) in the HoLEP group (*P*=0.12). No urinary incontinence episodes occurred at 12-month follow-up. Overall, at 12-month follow-up, there was no evidence of difference between the two treatments (RR 0.71, 95% CI 0.33 to 1.53).

We downgraded by three levels the quality for this outcome because of RoB (selection bias and detection bias suspected; performance and attrition bias) and very serious imprecision (GRADE evidence: very low). Very low-quality evidence means that the estimate of effect is very uncertain and confidence in the estimate is small.

Strictures

Bladder neck contracture occurred post operatively in one (2%) patient in the HoLEP group versus no stricture episodes in the GL-XPS group (RR 0.31, 95% CI 0.01 to 7.55; *P*=0.5).

We downgraded by three levels the quality for this outcome because of RoB (selection bias and detection bias suspected; attrition bias) and very serious imprecision (GRADE evidence: very low). Very low-quality evidence means that the estimate of effect is very uncertain and confidence in the estimate is small.

Urinary tract infection

One (2%) patient in the GL-XPS group experienced UTI during follow-up versus no patients in the HoLEP group (RR 2.83, 95% CI 0.12 to 67.97; *P*=0.5).

We downgraded by three levels the quality for this outcome because of RoB (selection bias and detection bias suspected; attrition bias) and very serious imprecision (GRADE evidence: very low). Very low-quality evidence means that the estimate of effect is very uncertain and confidence in the estimate is small.

Urinary retention

Six (11%) patients in the GL-XPS group and 2 (4%) patients in the HoLEP group had urinary retention (RR 2.83, 95% CI 0.60 to 13.37; *P*=0.2). In the GL-XPS group, three patients failed first trial of void, two had clot retention, and one had early retention (within the first month post operatively) while, in the HoLEP group, one patient failed first trial of void and one had clot retention.

We downgraded by three levels the quality for this outcome because of RoB (selection bias and detection bias suspected; attrition bias) and very serious imprecision (GRADE evidence: very low). Very low-quality evidence means that the estimate of effect is very uncertain and confidence in the estimate is small.

Procedural transfusions

There was no evidence that procedural transfusions occurred at a different rate in the GL-XPS group (two blood transfusions in one patient) and HoLEP group (no transfusions) (*P*=0.5).

Bleeding (procedural and post operative)

The postoperative complications table reported that three (6%) patients in the GL-XPS group and two (4%) in the HoLEP group had postoperative haematuria (*P*=0.7). The authors reported that 12 (23%) patients in the GL-XPS group versus three (6%) patients in the HoLEP group required hospitalisation for more than 1 day because of postoperative haematuria (RR 3.8, 95% CI 1.1 to 12.6; *P*=0.03).

Conversion to TURP

This outcome reflected the rate of conversion to monopolar TURP for haemostasis and for residual prostate tissue. In the GL-XPS group, 13 (25%) patients were converted to monopolar TURP for haemostasis versus two (4%) patients in the HoLEP group (RR 6.1, 95% CI 1.5 to 25.8; *P*=0.013). The number of patients requiring conversion to monopolar TURP because of residual prostate tissue was higher in the GL-XPS group, eight (15%) patients, than in the HoLEP group (no patients), although this difference was not significant (*P*=0.054).

Capsular perforation

Capsular perforation occurred in three (6%) patients in the GL-XPS group and in one (2%) patient in the HoLEP group (*P*=0.36).

Other procedure-related adverse events

Operative bladder wall injury occurred in one (2%) patient in the GL-XPS group versus four (8%) patients in the HoLEP group (*P*=0.19). Epididymo-orchitis occurred in two (4%) patients in the GL-XPS group versus no patients in the HoLEP group (*P*=0.3). Urosepsis occurred in one (2%) patient in the GL-XPS group versus no patients in the HoLEP group (*P*=0.5). Residual prostate adenoma occurred in two (4%) patients in the GL-XPS group versus no patients in the HoLEP group (*P*=0.3). Prostatic urethral stone + encrusts occurred in one (2%) patient in both the GL-XPS and HoLEP groups (*P*=1.0).

[C0002] – How do the harms relate to dosage or frequency of applying LBO laser?

[C0004] – How does the frequency or severity of harms change over time or in different settings?

[C0005] – What are the susceptible patient groups that are more likely to be harmed through the use of LBO laser PVP?

We did not find any evidence to answer these research questions.

[C0007] – Are LBO laser PVP and the comparators associated with user-dependent harms?

GL-XPS versus TURP

Long learning curves and procedural volume in the operating centre or the specific user could be important factors that might influence clinical outcomes. The GOLIATH Study [1–3] reported that all surgeons were licensed urologists trained and experienced with TURP; however, prior surgical experience with XPS varied widely among surgeons, from <10 cases to >500 cases. The surgical technique for GL-XPS was standardised according to previously published recommendations [31] and updated to the specification for using the XPS laser device, including MoXy fibre [32]. Surgeon were evaluated for adherence to the standard technique before being allowed to randomise patients.

In the 2015 study by Bachmann et al. [2], possible correlations between surgeon experience (proportion of time spent lasering) and patient-reported outcomes (OABq-SF) were assessed, but the authors reported that no statistically significant correlation was found.

GL-XPS versus HoLEP

In the Elshal et al. trial [5], it was stated that all procedures were performed by a single surgeon experienced in both techniques (they had performed >1200 HoLEP and 400 GreenLight procedures).

7 POTENTIAL ETHICAL, ORGANISATIONAL, PATIENT AND SOCIAL, AND LEGAL ASPECTS (ETH, ORG, SOC, LEG)

7.1 *Research questions*

**2. Organisational**	
2.2. Does comparing the new technology to the defined, existing comparator(s) point to any differences that might be organisationally relevant?	Yes
A shorter length of hospital stay after LBO laser PVP would allow better management of hospitalisations, with consequent savings for the healthcare system.	

Element ID	Research question
G0001	How does LBO laser PVP affect the current work processes in terms of frequency of completion as a day-case?

7.2 *Results*

Two further research questions related to organisational aspects were developed: length of hospital stay and frequency of completion as a day-case. In the comparison GL-XPS versus TURP, length of hospital stay was assessed by both the GOLIATH Study [1–3] and Jovanović et al. [4] and was significantly shorter in the GL-XPS group than in the TURP group (pooled estimate: MD –46 h, 95% CI –74 to –18; *P*=0.001) (see D0005). This aspect should be considered when performing economic analyses because its impact on costs can be relevant. By contrast, in the comparison GL-XPS versus HoLEP, length of hospital stay did not differ significantly between the two groups (MD 9.6 h, 95% CI –0.0 to 19.2; *P*=0.056).

[G0001] – How does LBO laser PVP affect the current work processes in terms of frequency of completion as a day-case?

We did not find evidence in the included studies to answer this research question. The GOLIATH Study, currently the largest comparative study on the use of GL-XPS, reported a mean length of hospital stay in the GL-XPS group of 65.5 h, without reporting how many procedures were performed as a day-case [1]. By contrast, an economic study considered for the development of a national assessment of this technology [33], reported that most procedures (93%) using GL-XPS were performed as day-surgery, in contrast to ~6% of TURP and none of the bipolar TURP procedures. However, the sample size of this study was very small, counting only 56 patients undergoing the GL-XPS procedure. To reach clear conclusions on this aspect, a specific literature search and analysis need to be performed.

8 DISCUSSION

GL-XPS is the LBO laser system for PVP that was assessed in the present document. It represents the latest evolution of the technology and has been available on the market since 2010. Although several studies assessing the previous generations of the system have been published, we decided to focus the present assessment on GL-XPS because it is the only system currently available on the market. The system is intended for surgical incision and/or excision, vaporisation, ablation, haemostasis and coagulation of soft tissue. Even if this theoretically allows its use across many specialties, currently only urological applications are known. The procedure is performed within the urology department in day-surgery or inpatient settings. Across European countries, the procedure is reimbursed under an umbrella code for transurethral prostatectomy despite the technology used, whereas only Austria and Germany have issued a specific reimbursement code. In 2017, the technology was in use within 754 centres across Europe, with a total of 30,372 procedures performed in the same year.

Information on the learning curve appears conflicting: whereas the manufacturer reports that up to 25 procedures are sufficient to be able to acquire independence, the authors of a study involving three operators from three different centres performing the procedure on 365 patients, concluded that >100 procedures were required [12].

In the latest guidelines from the EAU [24], PVP with GL-XPS is recommended as an alternative to TURP, with the recommendation rated as 'strong'. However, for the treatment of specific groups of patient receiving antiplatelet or anticoagulant therapy with a prostate volume <80 ml, the recommendation is rated as 'weak' because of the low level of evidence available. In 2018, during the finalisation of the present assessment, guidelines from the AUA and CUA were published. Although all guidelines are essentially based on the same trial (the GOLIATH Study), the strength of recommendation is rated as 'strong' by EAU and CUA, whereas is only rated as 'moderate' by AUA. However, the guideline recommendations were developed using the whole body of evidence available for all the three generations of the system, whereas the present assessment focused on the latest generation (180 W GL-XPS), for which the body of evidence is more limited despite the system having been in clinical use for almost a decade. The three generations mainly differ in terms of the laser source and power, which could affect the generalisability of study results.

In the treatment of BPH, the claimed benefits of GL-XPS include: shorter hospital length of stay (because the procedure can be done as a day-case procedure); shorter duration of catheterisation; quicker return to normal activity; reduction in pain; reduction in hospital readmissions; and reduced risk from capsular perforation, bleeding and TURP syndrome. Moreover, it can be used in patients taking anticoagulants and those with larger prostates.

The PICO of the present assessment was structured to reflect the variety of alternative options to LBO laser PVP, available for the treatment of BPH across the different population groups and to verify whether the claimed benefits of GL-XPS were supported by evidence from clinical studies. Outcomes were rated by the authoring team according to their importance as 'critically important' and 'important but not critical'.

We identified only comparative studies reporting on two of the comparisons that we were interested in: GL-XPS versus TURP and GL-XPS versus HoLEP. No comparative evidence fulfilling the defined inclusion criteria was identified for the other comparisons, such as studies assessing GL-XPS versus TUIP, OP, bipolar enucleation, ThuVAP, diode laser vaporisation or laser enucleation. Follow-up reached 24 months for the GL-XPS versus TURP comparison and 12 months for the GL-XPS versus HoLEP comparison. Although the first can be considered a minimum timeframe for the assessment of some of the selected outcomes, the latter might be too short. Only one of the included trials was multicentric. Given that the procedure can be highly operator dependent, only a multicentric and multioperator design can guarantee the generalisability of the results.

GL-XPS versus TURP

The body of evidence on GL-XPS versus TURP comprised two trials, the GOLIATH Study [1–3], a sponsored non-inferiority trial, and that by Jovanović et al. [4].

All the critical outcomes were assessed by at least one trial and a SoF table was provided for each outcome (Table 1). Only two of the effectiveness outcomes (i.e., mortality and frequency of completion as a day-case) were not reported in either of the included trials. Although mortality is a very relevant outcome *per se*, it might be unlikely to occur given the characteristics of the procedure and the patients. However, the frequency of completion as a day-case should have been reported because it is one of the claimed benefits of the technology. Instead, only length of hospital stay was reported. Among the critically important safety outcomes, urinary incontinence and urethral and bladder neck strictures were the only outcomes reported by both the included trials. Subjective outcomes measures were clearly defined only in the GOLIATH Study [1–3].

We used the GRADE approach for rating the quality of evidence. Two of the critical outcomes (Qmax and PVR improvement at 24 months) were rated as moderate quality according to GRADE. The remaining evidence regarding GL-XPS versus TURP was rated from low to very low. The first serious concern was regarding the RoB. Both the GOLIATH study [1–3] and Jovanović et al. [4] were considered at risk of performance bias for outcomes likely to be influenced by behaviour, whereas, for outcomes not likely to be influenced by behaviour, we assumed that the risk of performance bias was unlikely. One of the two studies [4] did not report the methods used to generate the random sequence and to conceal treatment allocation, and whether the outcome assessor (for any critical outcome) was blinded; thus, selection bias and detection bias (for subjective outcomes) were both suspected; in addition, for several outcomes, the trial did not report sufficient data to allow us to perform a meta-analysis. The second reason for a further downgrading was because of imprecision resulting from a small sample size and wide CIs. In addition, we had concerns about sample size calculations. The GOLIATH Study [1–3] had a non-inferiority design but the sample size calculation did not provide sufficient elements to assess its adequacy to demonstrate non-inferiority. In addition, the proportion of patients (dichotomous data) within the non-inferiority margins (3 points for IPSS score, and –5 ml/s for Qmax) was not reported. In Jovanović et al. [4], there was no sample size calculation at all. Some outcomes with wide CIs and/or very few events were considered to have very serious imprecision and were downgraded by two levels.

The GOLIATH Study [1–3] reported that, for the primary outcomes (IPSS and Qmax improvement at 6, 12 and 24 months), GL-XPS was non-inferior to TURP (GRADE evidence for IPSS = low; GRADE evidence for Qmax at 24 months = moderate).

For the other critical outcomes, the following showed no difference between GL-XPS and TURP: PVR volume, rate of dysuria (pooled), IIEF-5, IPSS-QoL, patient satisfaction, surgical retreatment, urinary incontinence, irritative symptoms, strictures (pooled), urinary retention and erectile dysfunction. The quality of evidence for these outcomes was low to very low, except for PVR at 24 months (GRADE: moderate). GL-XPS was superior to TURP in terms of a lower rate of re-intervention at 30 days and need for transfusions (GRADE: low). Conversely, TURP was superior to GL-XPS in self-reported overactive bladder symptoms and incontinence (GRADE: low).

Other outcomes rated as important but not critical showed some benefits in favour of GL-XPS with to TURP: both length of catheterisation and length of hospital stay were shorter in the GL-XPS group (these outcomes are correlated and, thus, collinearity might be present). Reductions in the length of catheterisation and of hospital stay could be relevant both clinically and for the use of resources; however, the evidence was judged very low and we have little confidence in the effect estimates.

Even if retrograde ejaculation was not included among the outcomes of interest of the present assessment, we acknowledged its relevance and looked at it within the studies. Retrograde ejaculation was reported only for the GOLIATH Study [1] and was similar between the two groups (88 patients from the GL-XPS group and 84 from the TURP group). No further analyses or comments were made by the authors of the study.

GL-XPS versus HoLEP

The body of evidence on GL-XPS versus HoLEP comprised only one study [5], with a non-inferiority design. A SoF table was provided for each outcome (Table 2). The primary outcome was IPSS at 12 months and the authors concluded that non-inferiority of GL-XPS versus HoLEP was demonstrated. No differences between the two groups were observed in most of the effectiveness outcomes reported, whereas significant differences in favour of HoLEP were observed in terms of

improvement in Qmax (at 4 and 12 months) and duration of catheterisation. Similarly, no differences between the two groups were observed in most of the safety outcomes reported, whereas significant differences in favour of HoLEP were observed in terms of bleeding (postoperative haematuria) and rate of conversion to TURP. However, the quality of the study was rated as very low for several issues. The methods used to conceal allocation and blinding of the outcome assessor were not clearly described. Consequently, the quality of the evidence was downgraded by one level because of serious concerns over RoB. Moreover, the study had a non-inferiority design, but the sample size calculation did not provide sufficient elements to assess its adequacy to demonstrate non-inferiority and, thus, we conclude that the sample size was too small to provide sufficient evidence for non-inferiority between the two interventions. In addition, the proportion of patients (dichotomous data) within the non-inferiority margins (3 points for IPSS score) was not reported. We also downgraded the study quality by other two levels for imprecision because of a small sample size and wide CIs.

9 CONCLUSION

We highlight that our systematic review did not find evidence of good quality supporting most of the claimed benefits of GL-XPS versus its comparators.

When comparing GL-XPS with TURP, the quality of evidence was judged as moderate according to GRADE for the outcomes Qmax and PVR improvement. This suggests that, for these two outcomes, GL-XPS is non-inferior to TURP. Given that the quality of evidence was from low to very low for the remaining effectiveness and safety critical outcomes (e.g., IPSS and IPSS-QoL scores, dysuria, overactive bladder and incontinence symptoms, re-intervention rate, urinary incontinence, strictures and irritative symptoms), our confidence in the effect estimates is limited.

Given the very low-quality evidence related to the GL-XPS versus HoLEP comparison, we have low confidence in the effect estimate to conclude for non-inferiority between the two interventions in terms of the following critical outcomes considered in the present assessment: IPSS and IPSS-QoL scores, improvement in Qmax and PVR volume, dysuria, erectile function, re-intervention rate, urinary incontinence, strictures, UTI and urinary retention.

Therefore, we suggest that further research is needed because the body of available comparative evidence does not cover the four groups defined within the scope of the present assessment. In particular, although evidence for men with a prostate volume between 30 ml and 80 ml and men with prostate volume >80 ml was available, no studies reporting specifically on men with prostate volume <30 ml and men at risk of bleeding sequelae who cannot stop anticoagulation therapy were identified. Moreover, the body of evidence refers only to two of the comparators defined within the scope of the present assessment, TURP and HoLEP. Follow-up exceeding 24 months should be considered for a proper assessment of some of the outcomes (especially re-intervention rate) and, thus, multicentric multioperator study designs with appropriate sample sizes are awaited.

10 REFERENCES

1. Bachmann A, Tubaro A, Barber N, et al. 180-W XPS GreenLight laser vaporisation versus transurethral resection of the prostate for the treatment of benign prostatic obstruction: 6-month safety and efficacy results of a European Multicentre Randomised Trial--the GOLIATH study. Eur Urol. 2014;65(5):931–42.
2. Bachmann A, Tubaro A, Barber N, et al. A European multicenter randomized noninferiority trial comparing 180 W GreenLight XPS laser vaporization and transurethral resection of the prostate for the treatment of benign prostatic obstruction: 12-month results of the GOLIATH study. J Urol. 2015;193(2):570–8.
3. Thomas JA, Tubaro A, Barber N, et al. A multicenter randomized noninferiority trial comparing GreenLight-XPS laser vaporization of the prostate and transurethral resection of the prostate for the treatment of benign prostatic obstruction: two-yr outcomes of the GOLIATH Study. Eur Urol. 2016;69(1):94–102.
4. Jovanović M, Džamić Z, Aćimović M, Kajmaković B, Pejčić T. Usage of GreenLight HPS 180-W laser vaporisation for treatment of benign prostatic hyperplasia. Acta Chir Iugosl. 2014;61(1):57–61.
5. Elshal AM, Elkoushy MA, El-Nahas AR, et al. GreenLight laser (XPS) photoselective vapo-enucleation versus holmium laser enucleation of the prostate for the treatment of symptomatic benign prostatic hyperplasia: a randomized controlled study. J Urol. 2015;193(3):927–34.
6. Guyatt GH, Oxman AD, Kunz R, Brozek J, Alonso-Coello P, Rind D, et al. GRADE guidelines 6. Rating the quality of evidence--imprecision. J Clin Epidemiol. 2011 Dec;64(12):1283–93.
7. NICE [Internet]. Guidance MTG29. GreenLight XPS for treating benign prostatic hyperplasia. June 2016 [cited 2018 Nov 6]. Available from https://www.nice.org.uk/guidance/mtg29.
8. Gravas S, Cornu JN, Drake MJ, et al. [Internet]. 2017 EAU Guidelines on Management of Non-Neurogenic Male Lower Urinary Tract Symptoms (LUTS), incl. Benign Prostatic Obstruction (BPO) [cited 2018 Nov 6]. Available from http://uroweb.org/wp-content/uploads/13-Non-Neurogenic-Male-LUTS_2017_web.pdf.
9. Tracey JM, Warner JN. Transurethral bipolar enucleation of the prostate is an effective treatment option for men with urinary retention. Urology. 2016 Jan;87:166–71.
10. Bach T, Muschter R, Sroka R, Gravas S, Skolarikos A, Herrmann TRW, et al. Laser treatment of prostatic obstruction: basic and physical differences. Eur Urol. 2012;61:317–25.
11. Gomez Sancha F, Rivera VC, Georgiev G, Botsevski A, Kotsev J, Herrmann T. Common trend: move to enucleation-Is there a case for GreenLight enucleation? Development and description of the technique. World J Urol. 2015 Apr;33(4):539-47.
12. Bastard C, Zorn K, Peyronnet B, Hueber PA, Pradère B, Rouprêt M, et al. Assessment of learning curves for 180-W GreenLight XPS photoselective vaporisation of the prostate: a multicentre study. Eur Urol Focus. 2017 Sep 23. pii: S2405-4569(17)30215-8.
13. Roehrborn, CG. Male lower urinary tract symptoms (LUTS) and benign prostatic hyperplasia (BPH). Med Clin North Am. 2011;9587–100.
14. Lim KB. Epidemiology of clinical benign prostatic hyperplasia. Asian J Urol. 2017;4(3):148–51.
15. Bosch JL, Bangma CH, Groeneveld FP, Bohnen AM. The long-term relationship between a real change in prostate volume and a significant change in lower urinary tract symptom severity in population-based men: the Krimpen study. Eur Urol. 2008;53:819–27.
16. Emberton M, Fitzpatrick JM, Garcia-Losa M, Qizilbash N, Djavan B. Progression of benign prostatic hyperplasia: systematic review of the placebo arms of clinical trials. BJU Int. 2008;102:981–6.
17. NICE [Internet]. Clinical guideline CG97. Lower urinary tract symptoms in men: management. June 2015 [cited 2018 Nov 28]. Available from https://www.nice.org.uk/guidance/cg97.
18. WHO [Internet]. Health statistics and information systems. Disease burden and mortality estimates [cited 2018 Nov 28]. Available from http://www.who.int/healthinfo/global_burden_disease/estimates/en/index1.html.

19. Kirby RS, Kirby M, Fitzpatrick JM. Benign prostatic hyperplasia: counting the cost of its management. BJU Int. 2010;105:901–2.
20. Errando-Smet C, Müller-Arteaga C, Hernández M, Lenero E, Roset M. Healthcare resource utilization and cost among males with lower urinary tract symptoms with a predominant storage component in Spain: The epidemiological, cross-sectional MERCURY study. Neurourol Urodyn. 2018 Jan;37(1):307–15.
21. Phillips B, et al. [Internet]. Oxford Centre for Evidence-based Medicine Levels of Evidence. Updated by Jeremy Howick March 2009. 1998 [cited 2018 Nov 27]. Available from http://www.cebm.net/oxford-centre-evidence-based-medicine-levels-evidence-march-2009/.
22. Abrams P, et al. The standardisation of terminology of lower urinary tract function: report from the Standardisation Sub-committee of the International Continence Society. Neurourol Urodyn. 2002;21:167.
23. Kupelian V, et al. Prevalence of lower urinary tract symptoms and effect on quality of life in a racially and ethnically diverse random sample: the Boston Area Community Health (BACH) Survey. Arch Intern Med. 2006;166:2381.
24. Gravas S, Cornu JN, Drake MJ, et al. [Internet]. 2018 EAU Guidelines on Management of Non-Neurogenic Male Lower Urinary Tract Symptoms (LUTS), incl. Benign Prostatic Obstruction (BPO) [cited 2019 Mar 8]. Available from https://uroweb.org/wp-content/uploads/EAU-Guidelines-on-the-Management-of-Non-neurogenic-Male-LUTS-2018-large-text.pdf.
25. AUA [Internet]. Benign Prostatic Hyperplasia: Surgical Management of Benign Prostatic Hyperplasia/Lower Urinary Tract Symptoms (2018) [cited 2019 Mar 8]. Available from https://www.auanet.org/guidelines/benign-prostatic-hyperplasia/lower-urinary-tract-symptoms-(2018).
26. CUA [Internet]. Canadian Urological Association guideline on male lower urinary tract symptoms/benign prostatic hyperplasia (MLUTS/BPH): 2018 update [cited 2019 Mar 8]. Available from https://www.cua.org/themes/web/assets/files/canadian_urological_association_guideline_on_male_lower_urinary_tract5616__3_.pdf.
27. Lee SWH, Chan EMC, Lai YK. The global burden of lower urinary tract symptoms suggestive of benign prostatic hyperplasia: A systematic review and meta-analysis. Sci Rep. 2017 Aug 11;7(1):7984.
28. Marszalek M, Ponholzer A, Pusman M, Berger I, Madersbacher S [Internet].Transurethral resection of the prostate. European Urology Supplements 8 (2009) 504–512 [cited 2018 Nov 21]. Available from http://eu-acme.org/europeanurology/upload_articles/Marszalek.pdf.
29. Barry MJ, Williford WO, Chang Y, et al. Benign prostatic hyperplasia specific health status measures in clinical research: how much change in the American Urological Association symptom index and the benign prostatic hyperplasia impact index is perceptible to patients? J Urol 1995;154:1770–4.
30. Barry MJ. Evaluation of symptoms and quality of life in men with benign prostatic hyperplasia. Urology 2001;58:25.
31. Muir G, Gomez Sancha F, Bachmann A, et al. Techniques and training with GreenLight HPS 120-W laser therapy of the prostate: position paper. Eur Urol. Suppl 2008;7:370–7.
32. Bachmann A, Muir GH, Collins EJ, et al. 180-WXPS GreenLight laser therapy for benign prostate hyperplasia: early safety, efficacy, and perioperative outcome after 201 procedures. Eur Urol. 2012;61:600–7.
33. Masucci L, Erman A, Krahn MD, Elterman D. Cost analysis of Greenlight photoselective vaporization of the prostate compared to transurethral resection of the prostate for benign prostatic hyperplasia. Can Urol Assoc J. 2018 Jun 19. doi: 10.5489/cuaj.5267.

APPENDIX 1: METHODS AND DESCRIPTION OF THE EVIDENCE USED

DOCUMENTATION OF THE SEARCH STRATEGIES

Date of searches: 13th November 2018

Timespan: from 1st January 2009 to 13th November 2018.

Language: English.

Limits: secondary studies, conference abstracts, note, comments, editorials have been excluded.

MEDLINE

#1	((prostatic OR prostate) AND hyperplasia)	26,668
#2	((prostatic OR prostate) AND hypertrophy)	4093
#3	(prostatic OR prostate) AND obstruction	5378
#4	((prostatic OR prostate) AND adenoma)	2411
#5	‘Prostatic Hyperplasia’[Mesh]	20,706
#6	‘Lower Urinary Tract Symptoms’[Mesh:NoExp]	2167
#7	‘Lower Urinary Tract Symptoms’	8110
#8	‘Prostatism’[Mesh]	531
#9	prostatism	1049
#10	‘Urinary Bladder Neck Obstruction’[Mesh]	4233
#11	‘Benign prostatic’	15,833
#12	#1 OR #2 OR #3 OR #4 OR #5 OR #6 OR #7 OR #8 OR #9 OR #10 OR #11	34,159
#13	Greenlight	338
#14	Green-light	2529
#15	‘Green light’	2529
#16	(lithium and triborate and laser) [Title/Abstract]	104
#17	(lithium and ‘tris borate’ and laser) [Title/Abstract]	0
#18	(LBO AND laser)	132
#19	(Photoselective AND vaporisation)[Title/Abstract]	384
#20	(Photoselective AND vaporization)[Title/Abstract]	49
#21	(Photoselective AND vaporesection)[Title/Abstract]	8
#22	(Photo-selective AND vaporization) [Title/Abstract]	12
#23	(Photo-selective AND vaporisation)[Title/Abstract]	3
#24	(Photo-selective AND vaporesection)[Title/Abstract]	0
#25	(Photo AND selective AND vaporisation)[Title/Abstract]	5
#26	(Photo AND selective AND vaporization)[Title/Abstract]	14
#27	(Photo AND selective AND vaporesection)[Title/Abstract]	0
#28	(Laser AND vaporisation)[Title/Abstract]	210
#29	(Laser AND vaporization)[Title/Abstract]	2213
#30	Photovaporization [Title/abstract]	81
#31	Photovaporisation[Title/abstract]	19
#32	#13 OR #14 OR #15 OR #16 OR #17 OR #18 OR #19 OR #20 OR #21 OR #22 OR #23 OR #24 OR #25 OR #26 OR #27 OR #28 OR #29 OR #30 OR #31	30,990
#33	#12 AND #32 Limits (English[lang] AND ((‘2009/01/01’[PDat] : ‘3000/12/31’[PDat]) NOT ((‘systematic review*’ OR (review and literature)) OR editorial OR note OR comment OR guideline))	**422**

Number of hits: 422

EMBASE

#1	'prostate hypertrophy'/exp	35,341
#2	'prostatism'/exp	819
#3	'prostatic obstruction'/exp	11
#4	'lower urinary tract symptom'/exp	12,637
#5	'bladder obstruction'/exp	3870

#6	('prostatic hypertrophy':ti,ab,kw OR 'prostatic obstruction':ti,ab,kw OR 'prostatic adenoma':ti,ab,kw)	5611
#7	#1 OR #2 OR #3 OR #4 OR #5 OR #6	47,183
#8	'greenlight':ti,ab,kw	748
#9	'green-light':ti,ab,kw	2622
#10	'urological laser system'	99
#11	('lithium':ti,ab,kw AND 'triborate':ti,ab,kw AND 'laser':ti,ab,kw)	87
#12	('lbo':ti,ab,kw AND 'laser':ti,ab,kw)	109
#13	('photoselective':ti,ab,kw AND '(vaporisation':ti,ab,kw)	120
#14	('photo-selective':ti,ab,kw AND '(vaporisation':ti,ab,kw)	9
#15	(photo' AND 'selective):ti,ab,kw AND '(vaporisation' OR 'vaporization' OR 'vaporisection))`':ti,ab,kw	69
#16	'laser vaporisation':ti,ab,kw	169
#17	'laser vaporization':ti,ab,kw	1143
#18	photovaporization:ti,ab,kw	152
#19	'green light'	2622
#20	#8 OR #9 OR #10 OR #11 OR #12 OR #13 OR #14 OR #15 OR #16 OR #17 OR #18 OR #19	4646
#21	#7 AND #20 ([article]/lim OR [article in press]/lim) AND [english]/lim	334

Number of hits: 334

COCHRANE LIBRARY

#1	'Green light':ti,ab,kw	133
#2	Greenlight:ti,ab,kw	89
#3	Green-light:ti,ab,kw	133
#4	'Lithium triborate laser':ti,ab,kw	2
#5	'Lithium tris borate laser':ti,ab,kw	0
#6	(LBO AND laser):ti,ab,kw	7
#7	(photoselective AND (vaporisation OR vaporization OR vaporesection)):ti,ab,kw	85
#8	(photo-selective AND (vaporisation OR vaporization OR vaporesection)):ti,ab,kw	4
#9	(laser AND (vaporisation OR vaporization OR vaporesection)):ti,ab,kw	248
#10	#1 OR #2 OR #3 OR #4 OR #5 OR #6 OR #7 OR #8 OR #9	368
#11	('prostatic hypertrophy'):ti,ab,kw OR ('prostatic obstruction'):ti,ab,kw OR ('prostatic adenoma'):ti,ab,kw	1281
#12	MeSH descriptor: [Prostatic Hyperplasia] this term only	1596
#13	MeSH descriptor: [Prostatitis] this term only	309
#14	MeSH descriptor: [Lower Urinary Tract Symptoms] this term only	263
#15	MeSH descriptor: [Urinary Bladder Neck Obstruction] this term only	168
#16	'prostatic hyperplasia'	2450
#17	#11 OR #12 OR #13 OR #14 OR #15 OR #16	3221
#18	#10 AND #17	150

Number of hits: 150 (149 trials and 1 review)

Total number of hits: 906

Final number of hits after duplicates removal: 599

DESCRIPTION OF THE EVIDENCE USED

Evidence tables of individual studies included for clinical effectiveness and safety

Table A1. Characteristics of included studies

Study ID	Objective	Study design (country/setting)	Participants		Outcomes			Follow up	Funding
			Intervention group	Control group	Primary	Secondary	Safety endpoints		
			GL-XPS	TURP					
**GOLIATH – NCT01218672** [1–3]	To compare transurethral resection of prostate (TURP) to photoselective vaporisation with the GreenLight XPS Laser System (GL-XPS) for the treatment of benign prostatic obstruction (BPO).	Open-label, multicentre, randomised, noninferiority trial<br><br>(29 centres in nine European countries)	Patients candidates for the surgical relief of BPO, with IPSS scores of ≥ 12 and prostate sizes ≤ 100 g<br>• Number: 136<br>• Age (years): 67.2±6.8<br>• MPV (TRUS, ml): 48.6±19.2<br>• PVR: 110.1±88.5<br>• PSA (ng/ml): 2.7±2.1<br>• IPSS score: 21.2±5.9<br>• Qmax (ml/s): 9.5±3.0<br>• IPSS-QoL: 4.6±1.1<br>• Anticoagulant use: 5 (3.7%)<br>• OABq-SF symptoms: 44.2±20.5<br>• OABq-SF health: 59.0±21.9<br>• ICIQ-UI SF: 3.9±4.7<br>• IIEF-5: 13.2±7.6	 • Number: 133<br>• Age (years): 66.7±6.6<br>• MPV (TRUS, ml): 46.2±19.1<br>• PVR: 109.8±103.9<br>• PSA (ng/ml): 2.6±2.1<br>• IPSS score: 21.7±6.4<br>• Qmax (ml/s): 9.9±3.5<br>• IPSS-QoL: 4.5±1.4<br>• Anticoagulant use: 9 (6.8%)<br>• OABq-SF symptoms: 42.9±20.8<br>• OABq-SF health: 62.6±21.7<br>• ICIQ-UI SF: 4.4±4.6<br>• IIEF-5: 13.7±7.5	- IPSS score<br>- Qmax (ml/s)<br>- Complication-free<br>- Surgical re-treatments	– Prostate volume (TRUS; ml)<br>– PVR (ml)<br>– PSA (ng/ml)<br>– IPSS-QoL<br>– QoL: OABq-SF symptoms, OABq-SF health, ICIQ-UI-SF, IIEF-5, EQ-5D, SF-36 Mental Health, SF-36 Physical Health.	- Bleeding<br>- Urinary tract infection<br>- Irritative symptoms[1]<br>- Stricture[2]<br>- Urinary incontinence<br>- Urinary retention<br>- Other	- 6 months<br>- 12 months<br>- 24 months	American Medical Systems
			GL-XPS	TURP					

[1] Irritative symptoms include pain and discomfort.

[2] Stricture includes meatal, urethral, and bladder neck stricture.

**Jovanović 2014 [4]**	To compare results of GreenLight XPS laser vaporisation of the prostate and transurethral re-section of the pros-tate (TURP) for treatment of BPH.	Randomised con-trolled trial<br><br>(Clinic of Urology, Clinical Center of Serbia, Bel-grade)	Patients with moderate or severe LUTS (IPSS > 16), failure of previous medical treatment with a washout period of at least 2 weeks, Qmax <15ml/s, PVR urine >100ml, prostate volume (TRUS) <100ml.		- IPSS score<br>- Qmax (ml/s)<br>- PVR	- Operative time<br>- Hemoglobin levels (preoper-ative / intraoper-ative)<br>- Duration of catheterisation<br>- Length of hos-pital stay	- Blood transfusion<br>- Capsule perfora-tion<br>- TUR syndrome<br>- Clot retention<br>- Dysuria/urge<br>- Bladder neck contracture<br>- Urethral stricture<br>- Urinary inconti-nence	- 1 month<br>- 3 months<br>- 6 months<br>- 12 months	Not reported
			• Number: 31<br>• Age (years)[3]: 66.3 (9.4)<br>• MPV (TRUS, ml): 61.8±22<br>• PVR: 106.2±25<br>• PSA (ng/ml): 2.6±1.8<br>• IPSS score: 27.2±2.3<br>• Qmax (ml/s): 6.9±2.2<br>• Patients preoperatively catheterized: 6 (19%)	• Number: 31<br>• Age (years)[3]: 67.1 (8.0)<br>• MPV (TRUS, ml): 60.3±20<br>• PVR: 114±21<br>• PSA (ng/ml): 2.8±1.4<br>• IPSS score: 27.9±2.7<br>• Qmax (ml/s): 6.4±2.0<br>• Patients preoperatively catheterized: 5 (16%)					
			**GL-XPS**	**HoLEP**					
**Elshal 2015 [5] - NCT01494337**	To assess the non-inferiority of the GreenLight XPS vapo-enucleation of the prostate ver-	Randomised noninferiority trial	Patients >50 years, refractory LUTS secondary to BPH, IPSS >15, QOL score ≥3, Qmax <15 ml/s or patients with acute urinary retention secondary to BPH in whom trial of voiding failed, and prostate size on preoperative TRUS of 40 to 150 ml.		- IPSS score	- Prostate vo-lume (TRUS; ml)<br>- PVR<br>- PSA<br>- Qmax<br>- IPSS-QOL	Perioperative and postoperative complications:<br>- Postop dysuria<br>- Postop pyrexia	- 1 month<br>- 4 months<br>- 12 months	Royal Victo-ria Hospital, Canada

[3] Median (IQR)

	sus HoLEP in reduction of LUTS secondary to BPH.	(Royal Victoria Hospital, Montreal, Quebec, Canada)	• Number: 50[4]<br>• Age (years): 74.1±8.8<br>• MPV (TRUS, ml): 83.3±27.8<br>• PVR: 172±137<br>• PSA (ng/ml): 5.3±12.6<br>• IPSS score: 23.0±4.8<br>• Qmax (ml/s): 8.0±3.0<br>• IPSS-QoL: 4.9±1.1<br>• Anticoagulant use:<br>- Aspirin: 11 (20.7%)<br>- Bridging by LMWH: 15 (24.3%)<br>• Indwelling catheter: 23 (43.4%)<br>IIEF-15: 45.8±17	• Number: 53<br>• Age (years): 71±9.3<br>• MPV (TRUS, ml): 87.1±28.1<br>• PVR: 146±105<br>• PSA (ng/ml): 5.6±4.4<br>• IPSS score: 22.4±5.6<br>• Qmax (ml/s): 7.5±1.3<br>• IPSS-QoL: 3.8±1.2<br>• Anticoagulant use:<br>- Aspirin: 6 (12%)<br>- Bridging by LMWH: 12 (24%)<br>• Indwelling catheter: 23 (46%)<br>IIEF-15: 55.6±15.4		- IIEF-15<br>- Dysuria<br>- Duration of catheterisation<br>- Length of hospital stay	- Operative prostate capsule violation<br>- Operative bladder wall injury<br>- Inability to void (retention)<br>- Postop haematuria (grade 2 early / grade 3a late)<br>- Anaemia requiring transfusion<br>- Epididymo-orchitis<br>- Urosepsis<br>- Recurrent urinary tract infection<br>- Postop urge urinary incontinence<br>- Postop stress urinary incontinence<br>- Residual prostate adenoma<br>- Bladder neck contracture<br>- Urethral stricture<br>- Prostatic urethral stone + encrusts		

Key: GL-XPS, GreenLight XPS; **TURP**, transurethral resection of prostate; **BPO**, benign prostatic obstruction; **IPSS**, International Prostate Symptom Score; **MPV**, mean prostate volume; **ml**, millilitre; **TRUS**, transrectal ultrasound; **PVR**, post-void residual urine volume; **PSA**, prostate specific antigen; **ng/ml**, nanogram/millilitre; **Qmax**, maximum urine flow rate; **ml/s**, millilitre/second; **IPSS-QoL**, International Prostate Symptom Score-Quality of Life; **OABq-SF**, Overactive Bladder Questionnaire Short Form; **ICIQ-UI SF**, International Consultation on Incontinence Questionnaire Short Form; **IIEF-5**, International Index of Erectile Function-5; **EQ-5D**, EuroQol-5D; **SF-36**, Short Form (36) Health Survey; **BPH**, benign prostatic hyperplasia; **LUTS**, lower urinary tract symptoms; **HoLEP**, holmium laser enucleation of prostate; **LMWH**, Low-molecular-weight heparin; **IIEF-15**, International Index of Erectile Function-15.

[4] Originally, 55 patients were allocated to the intervention group; one patient did not receive the allocated intervention, and 4 other patients were excluded from final analysis for other reasons.

Risk of bias tables

Table A2. Risk of bias – study level (RCTs)

Trial	Random sequence generation (selection bias)	Allocation concealment (selection bias)	Blinding of participants and personnel (performance bias): objective outcomes	Blinding of participants and personnel (performance bias): subjective outcomes	Blinding of outcome assessment (detection bias): objective outcome	Blinding of outcome assessment (detection bias): subjective outcome	Incomplete outcome data (attrition bias)	Selective reporting (reporting bias)
GOLIATH	low	low	low	high[1]	low	unclear[2]	low	low
Jovanović	unclear[2]	unclear[2]	low	high[1]	low	unclear[2]	low	high[3]
Elshal	low	unclear[2]	low	high[1]	low	unclear[2]	high[4]	high[5]

[1] This was an open-label trial, the performance bias is deemed possible for subjective outcomes.
[2] No information reported.
[3] Point estimates at different time follow-up as well as standard deviations for several continuous data were not reported.
[4] Five patients in the HoLEP group versus no patients in the GL-XPS group were excluded from final analyses; intention-to-treat analysis was not performed.
[5] Means and standard deviations for the outcome erectile function at 4-months and at 12-months were not reported.

Table A3. Risk of bias – outcome level (RCTs)

Trial	Blinding of outcome assessment (detection bias)	Incomplete outcome data (attrition bias)	Selective outcome reporting (reporting bias)	Other source of bias	Overall risk of bias - outcome level
**Reduction of symptoms using the IPSS score**					
GOLIATH	unclear[1]	low	Low	low	unclear
Jovanović	unclear[1]	low	high[2]	low	high
Elshal	unclear[1]	high[3]	Low	low	high
**Improvement of QoL using the IPSS-QoL score**					
GOLIATH	unclear[1]	low	low	low	unclear
Elshal	unclear[1]	high[3]	low	low	high
**Improvement in Qmax (ml/s)**					
GOLIATH	Low	low	low	low	low
Jovanović	unclear[1]	low	high[2]	low	high
Elshal	unclear[1]	high[3]	low	low	high
**Improvement in PVR volume (ml)**					
GOLIATH	Low	low	low	low	low
Jovanović	unclear[1]	low	high[2]	low	high

Elshal	unclear[1]	high[3]	low	low	high
**Dysuria**					
GOLIATH	Low	low	low	low	low
Jovanović	unclear[1]	low	high[2]	low	high
Elshal	unclear[1]	high[3]	low	low	high
**Dysuria (VAS)**					
Elshal	Low	high[3]	low	low	high
**Patient reported outcomes: Erectile function (IIEF-5)**					
GOLIATH	unclear[1]	low	low	low	unclear
**Patient reported outcomes: Erectile function (IIEF-15)**					
Elshal	unclear[1]	high[3]	high[4]	low	high
**Patient reported outcomes: OABq-SF Symptoms**					
GOLIATH	unclear[1]	low	low	low	unclear
**Patient reported outcomes: OABq-SF Health**					
GOLIATH	unclear[1]	low	low	low	unclear
**Patient reported outcomes: ICIQ-UI SF**					
GOLIATH	unclear[1]	low	low	low	unclear
**Rate of re-intervention at 30 days**					
GOLIATH	Low	low	low	low	low
Elshal	unclear[1]	high[3]	low	low	high
**Rate of re-intervention at 6 months**					
GOLIATH	Low	low	low	low	low
Elshal	unclear[1]	high[3]	low	low	high
**Rate of re-intervention at 12 months**					
GOLIATH	Low	low	low	low	low
Elshal	unclear[1]	high[3]	low	low	high
**Rate of surgical re-treatment for obstruction 0-24 months**					
GOLIATH	Low	low	low	low	low
**Urinary incontinence**					
GOLIATH	low	low	low	low	low
Jovanović	unclear[1]	low	high[2]	low	high
Elshal	unclear[1]	high[3]	low	low	high

**Irritative symptoms**					
GOLIATH	low	low	low	low	low
**Strictures**					
GOLIATH	low	low	low	low	low
Jovanović	unclear[1]	low	high[2]	low	high
Elshal	unclear[1]	high[3]	low	low	high
**Urinary tract infection**					
GOLIATH	low	low	low	low	low
Elshal	unclear[1]	high[3]	low	low	high
**Urinary retention**					
GOLIATH	low	low	low	low	low
Elshal	unclear[1]	high[3]	low	low	high
**Erectile dysfunction**					
GOLIATH	low	low	low	low	low

[1] No information reported.
[2] Point estimates at different time follow-up as well as standard deviations were not reported.
[3] Five patients in the HoLEP group versus no patients in the GL-XPS group were excluded from final analyses; intention-to-treat analysis was not performed.
[4] Means and standard deviations for the outcome erectile function at 4-months and at 12-months were not reported.

List of ongoing and planned studies

Table A4. List of ongoing studies on GreenLight XPS (180 W). Only studies with estimated completion date from 2018 are reported

Study Identifier [Status]	Estimated completion date	Study type	Number of patients	Intervention	Comparator	Patient population	Endpoints
NCT03736512<br>[Not yet recruiting]	May-2021	Observational (Patient Registry)	300	GreenLight XPS™ 532 nm Laser System with MoXy™ laser fiber	Not applicable.	Men diagnosed with BPH for whom GreenLight Laser therapy is recommended by their physician and eligible for inclusion.	Not provided.
NCT03318991<br>[Enrolling by invitation]	Jun-2019	Interventional;<br>Randomised;<br>Parallel Assignment.	100	***Active comparator***<br>GreenLight laser<br>180 W Greenlight laser is used for vaporesection of the prostate.<br>***Active comparator***<br>Thulium laser<br>200 W Thulium laser is used for enucleation of the prostate.	-	Patients with symptomatic BPO; patients' age greater than 50 years, Qmax <15 ml/second and an IPSS ≥10.	*Primary*<br>- IPSS at 1 year<br>*Secondary*<br>- Qmax at 1 year;<br>- QoL score at 1 year.
NCT03305861<br>[Active, not recruiting]	Dec-2018	Interventional;<br>Randomised;<br>Parallel Assignment.	150	***Experimental***<br>200 W Thulium laser enucleation of prostate (ThuLEP).<br>***Experimental***<br>180 W Greenlight laser enucleation of prostate (Green LEP).	***Active comparator***<br>Holmium laser enucleation of prostate (HoLEP).	Patients' age ≥40 years; LUTS secondary to BOO due to BPH who failed medical treatment; IPSS >15 and bother score (QOL) ≥3 (according to IPSS question 8); Qmax <15 ml/sec with at least 125 ml voided volume or patients with acute urine retention secondary to BPH who failed trial of voiding on medical treatment; ASA score ≤3; TRUS prostate size ≥80 ml.	*Primary*<br>- Voiding and storage symptoms improvement (at 6 months) using IPSS.<br>- Sexual function changes (at 6 months) using IIEF.<br>*Secondary*<br>- Operative time (in minutes)<br>- Postoperative complication (at 12 months) usinf the Clavien-Dindo classification for post-operative complications.

Study Identifier [Status]	Estimated completion date	Study type	Number of patients	Intervention	Comparator	Patient population	Endpoints
NCT03297281<br>[Recruiting]	May-2020	Interventional;<br>Randomised;<br>Parallel Assignment.	386	***Experimental***<br>Maintenance of OAC in surgery of BPH by PVP.	***Active comparator***<br>Discontinuation of OAC in surgery of BPH by PVP.	Prostate volume ≤80 gr; Micturition disorders resistant to medical treatment related to HBP and/or complications related to BPH (retention, lithiasis); Patient candidate for PVP; Patient under AVK treatment for more than 3 months with an objective of INR between 2 and 3 or patient under DOAC for more than 3 months; Unprotected major; Patient affiliated to a social security scheme or equivalent; Patient is willing and able to comply with all study requirements and to sign a study-specific informed consent form.	*Primary*<br>- Number of patients with at least one complication classified higher or equal to grade 2 according to the Clavien classification related to maintenance of OAC during the surgical resection of BPH by laser at 1 month.<br><br>*Secondary*<br>- Number of patients with at least one haemorrhagic complication related to maintenance of OAC during the surgical resection of BPH by laser at 1 month, 3 months and 6 months.<br>- Number of patients with at least one thrombotic complication related to maintenance of OAC during the surgical resection of BPH by laser at 1 month, 3 months and 6 months.<br>- Duration of hospitalisation related to perioperative management of anticoagulants at discharge.<br>- Prostatic residual volume at 1 month, 3 months, and 6 months.<br>- PSA level at 1 month, 3 months, and 6 months.<br>- IPSS at 1 month, 3 months and 6 months.<br>- ICS at 1 month, 3 months and 6 months.<br>- Volume of post-voiding residue at 1 month, 3 months and 6 months.

Study Identifier [Status]	Estimated completion date	Study type	Number of patients	Intervention	Comparator	Patient population	Endpoints
NCT02401581<br>[Recruiting]	Aug-2019	Interventional;<br>Single Group Assignment.	150	***Experimental***<br>Early removal of the catheter after a PVP procedure with GreenLight XPS 180 W	Not applicable.	Patients with (LUTS); IPSS ≥15 despite medical treatment >1 month if monotherapy or >3 months if bitherapie OR AUR non-medical after the 1st failure to remove the catheter OR acute prostatitis OR macroscopic haematuria of prostatic origin; Prostate volume >30 cc by TRUS ; IPSS Qol ≥3 has at inclusion; PSA ≤4 ng/ml; if PSA between 4 and 10 then PSA L/T ≥25% or negative PBP <6 months; Accommodation <50 km; Company available for the return at home and monitoring first post-operative night; Patient sign the informed consent; Patient covered by social security or other health insurance.	*Primary*<br>- Failure rate of a limited catheterisation duration of 3 hours post- operative.<br><br>*Secondary*<br>- Total dose of energy (during 24 hours hospitalisation).<br>- Duration of re-catheterisation (during 24 hours hospitalisation).
NCT02332538<br>[Active, not recruiting]	Dec-2018	Interventional;<br>Randomised;<br>Parallel Assignment.	182	***Active Comparator***<br>GreenLight PVEP using XPS 180W system.<br><br>***Active Comparator***<br>Holmium laser enucleation of prostate (HoLEP).<br><br>***Active Comparator***<br>Bipolar TURP in saline	-	Patients' age ≥50 years; LUTS secondary to BOO due to BPH who failed medical treatment; IPSS >15 and bother score (QOL) ≥3 (according to IPSS question 8); Qmax <15 ml/sec with at least 125 ml voided volume or Patients with acute urine retention secondary to BPH who failed trial of voiding on medical treatment; ASA score ≤3; TRUS prostate size (≥80 ml).	*Primary*<br>Re-treatment (at 2 years)<br><br>*Secondary*<br>- Change in symptoms score (at 2 years).<br>- Urine flow rate (in ml/sec; at 2 years).

Abbreviations: BPH, benign prostatic hyperplasia; BPO, benign prostate obstruction; Qmax, maximum flow rate; IPSS, International Prostate Symptom Score, LUTS; lower urinary tract symptoms; BOO, bladder outlet obstruction; ASA, American society of anaesthesiologists; TRUS, transrectal ultrasonography; IIEF, international index of erectile function questionnaire; OAC, oral-anticoagulant; PVP, photoselective vaporisation of prostate; AVK, anti vitamin K; INR, international normalised ratio; DOAC, direct oral anti-coagulants; ICS, international continence society; AUR, acute urinary retention; PVEP, photoselective vapo-enucleation of the prostate; PKVP, plasma kinetic vaporisation of the prostate;

Sources: www.clinicaltrials.gov Trials with status labelled as 'unknown' has been excluded. Searches performed on 15th November 2018.

Applicability tables

Table A5. Summary table characterising the applicability of a body of studies

Domain	Description of applicability of evidence
Population	The body of evidence does not cover the four groups defined within the scope of the present assessment. In particular, while evidence for men with prostate volume between 30 and 80 ml and men with prostate volume over 80 ml was available, no studies reporting specifically on men with prostate volume less than 30 ml and men at risk of bleeding sequelae who cannot stop anti-coagulation therapy were identified.
Intervention	The intervention was performed using the same technology among the included studies.
Comparators	The body of evidence refers only to two of the comparators defined within the scope of the present assessment, TURP and HoLEP. However, it needs to be highlighted that TURP represents the most performed procedure for the management of BPH and has been performed since decades in large volumes and HoLEP is considered the best treatment option for large prostates.
Outcomes	The outcomes reported in the included studies matched quite well the selection of outcomes performed within the scope of the present assessment. Only two of the effectiveness outcomes, i.e. mortality and frequency of completion as a day case, were not reported in any of the included trials. While mortality is surely a very relevant outcome *per se*, it may not be likely to occur given the characteristics of procedure and patients. On the other hand, the frequency of completion as a day case should have been reported since it is one of the claimed benefits of the technology. Instead, only length of hospital stay was reported. Among the critically important safety outcomes, urinary incontinence and urethral and bladder neck strictures were the only reported by all the included trials.<br>Follow-up reached 24 months for the comparison GL-XPS versus TURP and 12 months for the comparison GL-XPS versus HoLEP. While the first can be considered a minimum time frame for the assessment of some of the selected outcomes, the latter may be too short.
Setting	Only one of the included trials was multicentric. Given that the procedure can be highly operator-depended, only a multicentric and multioperator design can guaranty generalisability of results.

Abbreviations: BPH, benign prostatic hyperplasia; GL-XPS, GreenLight XPS; TURP, transurethral resection of prostate; HoLEP, Holmium laser enucleation of prostate.

APPENDIX 2: REGULATORY AND REIMBURSEMENT STATUS

Table A6. Regulatory status of the GreenLight XPS Laser System (Boston Scientific)

Country	Institution issuing approval	Authorisation status yes/no/ ongoing	Verbatim wording of the (anticipated) indication(s)	Specified contra-indications
Europe	CE mark<br>29/04/2010	Yes	*The GreenLight XPS Laser System is intended for the surgical incision/excision, vaporization, ablation, hemostasis and coagulation of soft tissue. All soft tissue is included, such as skin, cutaneous tissue, subcutaneous tissue, striated and smooth tissue, muscle, cartilage meniscus, mucous membrane, lymph vessels and nodes, organs and glands.*	*GreenLight 532 nm Laser System should only be used by a qualified and trained surgeon. The use of a GL 532 nm Laser System is contraindicated in patients:*<br>*• Whose general medical condition contraindicates surgical intervention*<br>*• Where appropriate anaesthesia is contraindicated by patient history*<br>*• Where tissue (especially tumours) has calcified*<br>*• For haemostasis of vessels over approximately two millimetres in diameter*<br>*• Where laser therapy is not considered the treatment of choice*<br>*• Uncontrolled bleeding disorders and coagulopathy*<br>*• Prostate cancer*<br>*• Acute urinary tract infection (UTI)*<br>*• Severe urethral stricture.*<br>*Use of the GreenLight 532 nm Laser Systems is contraindicated in the presence of severe urethral strictures; however, a system can be used in the treatment of urethral strictures with proper cautions. A severe stricture is any stricture with visible narrowing via urethrography or ultrasonography, with near total obstruction that makes passage of instruments difficult or dangerous. Care should be taken to avoid injury to urethral tissue.*
USA	FDA<br>11/09/2009	Yes	*Clearance for the GreenLight XPS™ Laser System for the surgical incision/ excision, vaporization, ablation and coagulation of soft tissue. All soft tissue is included, such as skin, cutaneous tissue, subcutaneous tissue, striated and smooth tissue, muscle, cartilage meniscus, mucous membrane, lymph vessels and nodes, organs and glands.*	*The laser system is contraindicated for patients who: are contraindicated for surgery, contraindicated where appropriate anesthesia is contraindicated by patient history, have calcified tissue, require hemostasis in >2mm vessels, have uncontrolled bleeding disorders, have prostate cancer, have acute urinary tract infection (UTI) or severe urethral stricture. Possible risks and complications include, but are not limited to, irritative symptoms (dysuria, urgency, frequency), retrograde ejaculation, urinary incontinence, erectile dysfunction, hematuria - gross, UTI, bladder neck contracture/outlet obstruct, urinary retention, perforation - prostate, urethral stricture.*

APPENDIX 3: CHECKLIST FOR POTENTIAL ETHICAL, ORGANISATIONAL, PATIENT AND SOCIAL AND LEGAL ASPECTS

**1**	**Ethical**	
1.1	Does the introduction of the new technology and its potential use/non-use instead of the defined, existing comparator(s) give rise to any new ethical issues?	No
1.2	Does comparing the new technology to the defined, existing comparators point to any differences that may be ethically relevant?	Yes
	A shorter length of hospital stay after LBO laser PVP would allow a better management of hospitalisations with consequent savings for the healthcare system.	
**2**	**Organisational**	
2.1	Does the introduction of the new technology and its potential use/non-use instead of the defined, existing comparator(s) require organisational changes?	No
2.2	Does comparing the new technology to the defined, existing comparator(s) point to any differences that may be organisationally relevant?	No
**3**	**Social**	
3.1	Does the introduction of the new technology and its potential use/non-use instead of the defined, existing comparator(s) give rise to any new social issues?	No
3.2	Does comparing the new technology to the defined, existing comparator(s) point to any differences that may be socially relevant?	No
**4**	**Legal**	
4.1	Does the introduction of the new technology and its potential use/non-use instead of the defined, existing comparator(s) give rise to any legal issues?	No
4.2	Does comparing the new technology to the defined, existing comparator(s) point to any differences that may be legally relevant?	No

APPENDIX 4: MISCELLANEOUS

Table A7. Documentation of queries to study authors in the assessment report

Study	Content of query	Reply received yes / no	Content of reply
Guibin 2010	▪ Full-text request	no	▪ No reply
Elshal 2015 [5]	▪ Follow-up outcome measures [mean ± SD or N (%)]	no	▪ No reply
Jovanović 2014 [4]	▪ Follow-up outcome measures [mean ± SD or N (%)]	no	▪ No reply

For the purpose of transparency, a separate document with comments on the 2nd draft assessment from external experts and the manufacturer(s) (fact check), as well as responses from the author, is available on the EUnetHTA website.
